# Genome-wide identification and expression analysis of glutathione S-transferase gene family in tomato: Gaining an insight to their physiological and stress-specific roles

**DOI:** 10.1371/journal.pone.0187504

**Published:** 2017-11-02

**Authors:** Shiful Islam, Iffat Ara Rahman, Tahmina Islam, Ajit Ghosh

**Affiliations:** 1 Department of Biochemistry and Molecular Biology, Shahjalal University of Science and Technology, Sylhet, Bangladesh; 2 Plant Breeding and Biotechnology Laboratory, Department of Botany, University of Dhaka, Dhaka, Bangladesh; National Botanical Research Institute CSIR, INDIA

## Abstract

Glutathione S-transferase (GST) refers to one of the major detoxifying enzymes that plays an important role in different abiotic and biotic stress modulation pathways of plant. The present study aimed to a comprehensive genome-wide functional characterization of GST genes and proteins in tomato (*Solanum lycopersicum* L.). The whole genome sequence analysis revealed the presence of 90 *GST* genes in tomato, the largest *GST* gene family reported till date. Eight segmental duplicated gene pairs might contribute significantly to the expansion of *SlGST* gene family. Based on phylogenetic analysis of tomato, rice, and *Arabidopsis* GST proteins, GST family members could be further divided into ten classes. Members of each orthologous class showed high conservancy among themselves. Tau and lambda are the major classes of tomato; while tau and phi are the major classes for rice and *Arabidopsis*. Chromosomal localization revealed highly uneven distribution of *SlGST* genes in 13 different chromosomes, where chromosome 9 possessed the highest number of genes. Based on publicly available microarray data, expression analysis of 30 available *SlGST* genes exhibited a differential pattern in all the analyzed tissues and developmental stages. Moreover, most of the members showed highly induced expression in response to multiple biotic and abiotic stress inducers that could be harmonized with the increase in total GST enzyme activity under several stress conditions. Activity of tomato GST could be enhanced further by using some positive modulators (safeners) that have been predicted through molecular docking of SlGSTU5 and ligands. Moreover, tomato GST proteins are predicted to interact with a lot of other glutathione synthesizing and utilizing enzymes such as glutathione peroxidase, glutathione reductase, glutathione synthetase and γ-glutamyltransferase. This comprehensive genome-wide analysis and expression profiling would provide a rational platform and possibility to explore the versatile role of *GST* genes in crop engineering.

## Introduction

Glutathione *S*-transferases (GSTs) are phase II metabolic isozymes, found mainly in the cytosol. GSTs catalyze the conjugation of tripeptide (γ-Glu-Cys-Gly) glutathione (GSH) to a variety of substrates such as endobiotic and xenobiotic compounds for the detoxification [1]. During this process, reduced glutathione (GSH) acts as a nucleophile that attacks electrophilic carbon, nitrogen or sulfur atom containing nonpolar toxic compounds [1]. Following conjugation and incorporation of the electrophilic groups into hydrophobic toxic chemicals, GST increases their solubility and promotes further metabolic process for the sequestration into vacuole or transferred to the apoplast [2]. GST could act on a wide range of substrates such as α,β-unsaturated carbonyls, arene oxides, halogen nitrobenzenes and quinones [1, 3, 4]. Besides GSH conjugation, several activities were found to be associated with GSTs in both plant and animal system such as high steroid isomerase activity, leukotriene biosynthesis, formation of oxylipins (precursor of jasmonic acid), double bond cis/trans isomerization, michael addition, mesotrione degradation, dehydroascorbate reduction, non-catalytic ligand binding and transport, signal transduction [5] and protection against ozone damages [6]. Due to its diverse cellular and metabolic role, it has been considered as one of the key members of plant stress modulation pathways [7].

In plants, GSTs exist as a multigene superfamily with three major subcellular localization patterns such as cytosolic, mitochondrial and microsomal. Amongst, cytosolic GST is the largest superfamily, while mitochondrial and microsomal GSTs are distinctive superfamilies. Cytosolic and mitochondrial GSTs comprise around 2% of total soluble plant proteins [3, 8]. Based on their genomic organization, sequence similarity and functions, plant GSTs could be categorized into several distinct classes, including tau (U), phi (F), theta (T), zeta (Z), lambda (L), dehydroascorbate reductase (DHAR), γ-subunit of the eukaryotic translation elongation factor 1B (EF1Bγ), tetrachlorohydroquinone dehalogenase (TCHQD), metaxin, Ure2p, hemerythrin (H), iota (I), microsomal prostaglandin E-synthase type 2 (mPGES-2) and glutathionyl-hydroquinone reductase (GHR) [9, 10]. Phi, tau, theta and zeta classes of GSTs are dimeric proteins possess a serine residue in their active sites; while TCHQD shares monomeric proteins with the presence of serine residue in the active site. However, DHAR, lambda, iota, hemerythrin, GHR, mPGES-2 and metaxin classes of GSTs have a catalytic cysteine in their active sites [6, 11]. The catalytic nature of rest of the classes, EF1Bγ and Ure2p is less known. Amongst the fourteen GST classes; phi, tau, DHAR, and lambda are highly specific for plant [12]. However, the most abundant plant GSTs are phi and tau [13].

*GST* genes have been identified from various plant species and found to be involved in different physiological, developmental and stress modulation pathways. Increased transcript level of *NbGSTU*1 and *NbGSTU*3 was observed in *Nicotiana benthamiana* during the infection of either *Colletotrichum destructivum* or *C*. *orbiculare* [14]. Transgenic tobacco plants overexpressing Nt107 (a GST) showed tolerance against different stresses [15]. Transgenic *Arabidopsis* plants overexpressing tomato *LeGSTU*2 showed enhanced resistance to salinity and drought stresses [16]. Similarly, ectopic expression of *GsGST* gene in transgenic tobacco plants showed enhanced tolerance towards drought and salt stresses [17]. Substrate affinity and catalytic activity of plant GSTs could be enhanced *in vivo* by the use of safeners and plant hormones such as auxins, abscisic acid, and ethylene. Safeners are agrochemicals known as herbicide antidotes which have the unique ability to elevate the expression of *GST* transcripts [18]. Safeners increase GST activity by utilizing an oxidized lipid-mediated or cyclopentenone-mediated signaling pathway to protect crop plants against applied thiocarbamate and chloroacetanilide herbicides [18].

Genome-wide analysis of *GST* genes have been conducted previously in various plant species, and identified 55 *GST* genes in *Arabidopsis* [19], 79 in rice [20], 84 in barley [8], 23 in sweet orange [21], 27 in Japanese larch [22], 59 in *G*. *raimondii* (cotton) and 49 in *G*. *arboreum* (cotton) [9], 49 in *C*. *rubella* [23]. Preliminary identification of tomato GST family members has been reported [24], but complete in-depth scrutiny of tomato GST family yet to perform. Tomato belongs to the genus of *Solanum* and considered as a crop of particular interest due to its natural fiber and nutritional importance. Tomato has relatively small genome size (950 Mb) and short life cycle (60–85 days) [25]. It also possesses a number of other useful characteristics such as seed production ability, the possibility of growing under different cultivation conditions, high self-fertility rate, ability of asexual propagation by grafting, easy way of controlling pollination and possibility to regenerate whole plants from different explants [25]. Thus, tomato is considered as an excellent model plant for both the basic and applied research programs.

In the present study, a genome-wide analysis of *GST* genes has been carried out in tomato and identified a total of 90 members. Each of these members was analyzed further to identify their chromosomal location, physiochemical characteristics, subcellular localization, conserved motifs, and domains. Further, transcript abundance of thirty tomato GST members was analyzed in different developmental, anatomical tissues and various abiotic and biotic stress conditions using publicly available microarray data. Among them, expression of fourteen transcripts has been analyzed by semi quantitative RT-PCR in response to salinity, dehydration and osmotic stresses. Moreover, total tomato GST activity has been measured towards these abiotic stress conditions, and the activity could be enhanced by applying various chemicals that have been predicted by the molecular docking study.

## Materials and methods

### Gene identification and nomenclature

A BLASTp search in the Sol Genomics Network (SGN) (http://www.solgenomics.net) [26] was performed to identify putative GST members in *S*. *lycopersicum*. Each class of GST protein sequence of rice and *Arabidopsis* was taken as a query in BLASTp search to find out all classes of GST members in *S*. *lycopersicum*. Tomato Genome protein sequences (ITAG release 2.40) was selected as a database in input parameters and maximum hits to show in advanced options was set 500 to conduct each BLASTp search. Rest of the other parameters persisted as default. The *Arabidopsis* Information Resource (TAIR release 10, https://www.arabidopsis.org) and the Rice Genome Annotation Project Database (RGAP release 7, http://rice.plantbiology.msu.edu/index.shtml) were used to download the published GST proteins of *Arabidopsis* and rice, respectively. BLASTp resulted members were categorized according to NCBI Conserved Domain Database search [27]. All the identified putative GST proteins were nomenclature as prefix “Sl” for *Solanum lycopersicum* followed class identifier (e.g., *SlGSTU*, *SlGSTF*, *SlGSTT*, *SlGSTZ*, *SlGSTL*, *SlTCHQD*, *SlDHAR*, *SlEF1Bγ*, *SlMGST* and *SlGHR* represents tau, phi, theta, zeta, lambda, TCHQD, DHAR, EF1Bγ, mPEGS-2 and GHR class, respectively) and a progressive number for each gene (e.g., *SlGSTU*1) according to the previously suggested system [28, 29]. Chromosomal location, strand position, CDS coordinate (5’ to 3’), length of gene, cDNA and CDS, exon number were retrieved from Sol Genomics Network database (http://www.solgenomics.net). Various physiochemical properties (such as molecular weight, polypeptide length, and pI) were calculated using ExPASy ProtParam software (http://web.expasy.org/protparam/). Prediction of subcellular localization was performed using CELLO v.2.5: sub-cellular localization predictor (http://cello.life.nctu.edu.tw/) [30] and pSORT prediction software (http://www.genscript.com/wolf-psort.html) [31]. Chloroplast localization was further confirmed by ChloroP (http://www.cbs.dtu.dk/services/ChloroP/) [32].

### Gene structure

The exon-intron structures of tomato *GST* genes were identified using online GSDS (Gene Structure Display Server, http://gsds1.cbi.pku.edu.cn/) [33] by comparing genomic and coding sequences. The result was exported from GSDS with the position of upstream-downstream, exon-intron and intron phase location. Intron phase was classified as 0, 1, 2 (Phase 0: between two consecutive codons; phase 1: splitting codons between the first and second nucleotides; Phase 2: between the second and third nucleotide of a codon).

### Chromosomal location

To map all *SlGST* genes, chromosome distribution diagram was drawn by IBS (Illustrator for Biological Sequences, http://ibs.biocuckoo.org/online.php), and Microsoft Office PowerPoint 2007 according to the information from Sol Genomics Network (http://www.solgenomics.net) and CDS coordinate information in Table 1.

**Table 1:** List of identified *GST* genes in tomato (*Solanum lycopersicum* L.) along with their detailed information and localization.

Sl no	Gene Name	Locus	Strand	CDS coordinate (5’ to 3’)	Length (bp)			Exons	Protein (aa)	MW (kDa)	pI	Localization
					Gene	cDNA	CDS					
1	*SlGSTU*1	Solyc01g081250.2	+	80511789–80512642	854	645	285	2	94	10.91	5.27	Cy^a^,Mt^b^
2	*SlGSTU*2	Solyc01g081260.1	+	80513804–80514687	884	381	381	2	126	15.09	7.87	Mt^a^,Cy^b^
3	*SlGSTU*3	Solyc01g081270.2	+	80516231–80517572	1342	1181	672	2	223	25.94	5.47	Cy^a^^,^^b^
4	*SlGSTU*4	Solyc01g081310.2	+	80541733–80543263	1531	1432	678	2	225	25.73	5.71	Cy^a^^,^^b^
5	*SlGSTU*5	Solyc01g086680.2	+	81642585–81644442	1858	949	675	2	224	25.72	6.36	Cy^a^^,^^b^
6	*SlGSTU*6	Solyc01g099590.2	-	89777069–89778325	1257	943	675	2	224	25.48	5.72	Cy^a^^,^^b^,Cp^a^,Pm^a^
7	*SlGSTU*7	Solyc02g081240.1	+	45268425–45269371	947	669	669	2	222	25.52	5.18	Cy^a^,Nu^b^
8	*SlGSTU*8	Solyc03g116120.1	-	65629271–65630010	740	675	675	2	224	25.88	5.93	Cy^a^^,^^b^
9	*SlGSTU*9	Solyc03g116130.1	-	65633586–65634464	879	702	702	2	233	26.65	5.39	Cy^a^^,^^b^
10	*SlGSTU*10	Solyc05g006730.2	+	1377080–1379013	1934	954	678	2	225	25.55	6.32	Cy^a^, Mt^b^
11	*SlGSTU*11	Solyc05g006740.2	+	1379095–1381005	1911	989	645	2	214	24.04	5.34	Cy^a^,Cp^a^,Nu^b^
12	*SlGSTU*12	Solyc05g006750.2	+	1381588–1382811	1224	979	690	2	229	26.31	5.10	Cy^a^^,^^b^
13	*SlGSTU*13	Solyc05g026210.1	-	40113650–40114827	1178	693	693	2	230	25.69	5.43	Cpa^a^,pm^b^
14	*SlGSTU*14	Solyc05g026220.1	-	40115985–40116607	623	483	483	2	160	18.22	5.75	Cy^a^,Cp^a^^,^^b^
15	*SlGSTU*15	Solyc06g069040.2	-	42840033–42844923	4891	4499	681	2	226	25.96	5.15	Cy^a^,Cp^b^
16	*SlGSTU*16	Solyc07g021460.1	-	18020257–18021808	1552	558	558	3	185	21.26	5.74	Cy^a^^,^^b^,Pm^a^
17	*SlGSTU*17	Solyc07g049330.1	-	59583071–59583410	340	300	300	2	99	11.13	5.36	Cy^a^^,^^b^
18	*SlGSTU*18	Solyc07g056420.2	+	64278679–64280483	1805	1012	663	2	220	25.39	5.37	Cy^a^^,^^b^
19	*SlGSTU*19	Solyc07g056430.2	+	64283887–64288015	4129	683	408	3	140	16.20	5.81	Cy^a^^,^^b^
20	*SlGSTU*20	Solyc07g056440.2	+	64287551–64289361	1811	1432	1075	2	222	25.71	5.60	Cy^a^^,^^b^
21	*SlGSTU*21	Solyc07g056450.2	+	64291231–64292940	1710	757	420	2	139	16.35	5.31	Cy^a^^,^^b^
22	*SlGSTU*22	Solyc07g056460.2	+	64293367–64295078	1712	969	663	2	220	25.76	6.36	Cy^a^^,^^b^
23	*SlGSTU*23	Solyc07g056470.2	+	64296528–64299942	3415	2719	663	2	220	25.51	5.76	Cy^a^^,^^b^
24	*SlGSTU*24	Solyc07g056480.2	+	64299666–64300689	1024	951	663	2	220	25.58	5.57	Cy^a^^,^^b^
25	*SlGSTU*25	Solyc07g056490.2	-	64300735–64302388	1654	1054	660	2	219	25.38	5.42	Cy^a^^,^^b^
26	*SlGSTU*26	Solyc07g056500.2	-	64305066–64306543	1478	567	439	3	220	25.54	7.61	Cy^a^^,^^b^
27	*SlGSTU*27	Solyc07g056510.2	-	64308885–64310376	1492	1030	660	2	219	25.41	6.10	Cy^a^^,^^b^
28	*SlGSTU*28	Solyc08g062570.1	+	51485839–51486255	417	417	417	1	138	16.37	6.29	Pm^a^,Cy^b^
29	*SlGSTU*29	Solyc09g011490.2	-	4809980–4811002	1023	882	660	2	219	24.96	5.75	Cy^a^^,^^b^
30	*SlGSTU*30	Solyc09g011500.2	-	4812615–4814198	1584	922	669	2	222	25.42	5.27	Cy^a^,Cp^b^
31	*SlGSTU*31	Solyc09g011510.2	-	4820455–4821500	1046	839	477	2	158	18.02	4.83	Cy^a^^,^^b^
32	*SlGSTU*32	Solyc09g011520.2	-	4826832–4827949	1118	1021	678	2	225	26.03	6.02	Cy^a^,Cp^a^
33	*SlGSTU*33	Solyc09g011530.1	-	4828798–4829088	340	340	300	2	96	11.22	5.23	Ec^a^,Cp^b^
34	*SlGSTU*34	Solyc09g011540.2	+	4835846–4836876	1031	907	669	2	222	25.45	5.05	Cy^a^,Nu^b^
35	*SlGSTU*35	Solyc09g011550.2	+	4837594–4838668	1075	864	660	2	219	25.36	5.45	Cy^a^^,^^b^
36	*SlGSTU*36	Solyc09g011560.2	+	4840014–4841512	1499	1043	654	2	217	25.16	5.70	Cy^a^^,^^b^
37	*SlGSTU*37	Solyc09g011570.2	+	4857466–4858799	1334	909	633	2	210	24.50	5.87	Cy^a^,Cp^b^
38	*SlGSTU*38	Solyc09g011580.2	+	4859444–4860733	1290	915	654	2	217	25.37	5.17	Cy^a^^,^^b^
39	*SlGSTU*39	Solyc09g011590.2	+	4866182–4867736	1555	903	654	2	217	25.11	5.30	Cy^a^^,^^b^
40	*SlGSTU*40	Solyc09g011600.2	+	4868723–4870339	1617	1033	654	2	217	25.27	5.90	Cy^a^,Nu^b^
41	*SlGSTU*41	Solyc09g011610.2	+	4873131–4873791	661	319	237	2	78	9.11	4.67	Cy^a^,Cp^b^
42	*SlGSTU*42	Solyc09g011620.1	+	4875106–4875916	811	648	648	2	215	24.76	5.39	Cy^a^,Cp^b^
43	*SlGSTU*43	Solyc09g011630.2	+	4888088–4889374	1287	917	657	2	218	24.86	5.53	Cy^a^^,^^b^
44	*SlGSTU*44	Solyc09g011640.2	+	4893328–4894820	1493	891	663	2	220	25.27	5.82	Cy^a^,Mt^b^
45	*SlGSTU*45	Solyc09g011650.2	+	4903272–4905768	2497	813	666	3	221	25.80	6.35	Cy^a^^,^^b^
46	*SlGSTU*46	Solyc09g063150.2	+	61196661–61198435	1775	889	663	2	220	25.89	6.19	Cy^a^^,^^b^,Cp^a^
47	*SlGSTU*47	Solyc09g091130.2	-	70484714–70485719	1006	684	675	2	224	26.40	7.70	Cy^a^^,^^b^,Cp^a^
48	*SlGSTU*48	Solyc09g091140.2	-	70486380–70488215	1836	715	672	2	223	26.04	4.95	Cy^a^^,^^b^
49	*SlGSTU*49	Solyc10g007620.1	-	1919334–1920575	1242	660	660	2	219	25.97	6.76	Cy^a^^,^^b^
50	*SlGSTU*50	Solyc10g007640.2	-	1923371–1924974	1604	1031	666	2	221	25.84	5.79	Cy^a^^,^^b^
51	*SlGSTU*51	Solyc10g084960.1	+	64311825–64313183	1359	666	666	2	221	25.28	5.31	Cy^a^^,^^b^
52	*SlGSTU*52	Solyc12g011300.1	+	4153362–4154489	1128	663	663	2	220	25.64	5.39	Cy^a^^,^^b^
53	*SlGSTU*53	Solyc12g011310.1	-	4159705–4160917	1213	660	660	2	219	25.62	6.91	Cy^a^,Nu^b^
54	*SlGSTU*54	Solyc12g011320.1	-	4162044–4163230	1187	660	660	2	219	25.45	5.99	Cy^a^^,^^b^
55	*SlGSTU*55	Solyc12g062730.1	-	34361197–34362753	1557	609	609	3	202	23.53	8.44	Cy^a^^,^^b^
56	*SlGSTU*56	Solyc12g097080.1	-	65724976–65726110	1135	666	666	2	221	25.85	6.92	Pm^a^,Cy^b^
57	*SlGSTU*57	Solyc12g036560.1	-	51590281–51590613	333	333	333	1	110	12.89	6.72	Mt^a^,Nu^b^
58	*SlGSTF*1	Solyc02g081340.2	+	45341113–45342471	1359	959	693	3	230	26.55	5.99	Cy^a^^,^^b^
59	*SlGSTF*2	Solyc06g009020.2	+	2965668–2967884	2217	1403	642	3	213	23.72	5.98	Cy^a^,Cp^b^
60	*SlGSTF*3	Solyc06g009040.2	+	2975638–2977590	1953	849	639	3	212	23.86	6.08	Cy^a^,Cp
61	*SlGSTF*4	Solyc09g074850.2	+	66668672–66671087	2416	1147	814	6	200	22.94	5.42	Cy^a^^,^^b^
62	*SlGSTF*5	Solyc12g094430.1	+	64641338–64643494	2156	666	666	3	221	25.07	6.85	Cy^a^,Nu^a^,Cp^b^
63	*SlGSTF*6	Solyc04g057890.2	-	55012081–55014935	2855	1125	807	3	268	31.53	9.26	Mt^a^^,^^b^,Nu^a^
64	*SlGSTT*1	Solyc02g081340.2	+	45341113–45342471	1359	959	693	3	230	26.55	5.99	Cy^a^^,^^b^
65	*SlGSTT*2	Solyc08g080900.2	-	64066712–64070723	4012	1198	753	7	250	28.58	9.25	Cy^a^^,^^b^
66	*SlGSTT*3	Solyc08g080910.2	-	64071456–64075162	3707	1369	753	7	250	28.69	9.21	Cy^a^^,^^b^,Nu^b^
67	*SlGSTT*4	Solyc12g056250.1	-	62208101–62210239	2138	711	711	7	236	26.55	6.06	Cy^a^,Mt^b^
68	*SlGSTL*1	Solyc04g009530.2	-	2943884–2949090	5207	1191	888	10	295	33.52	6.25	Cy^a^,Cp^b^
69	*SlGSTL*2	Solyc09g007150.2	+	775043–778675	3633	953	717	10	238	27.59	4.97	Cy^a^^,^^b^
70	*SlGSTL*3	Solyc10g084400.1	-	63957353–63959693	2340	708	708	10	235	27.18	5.06	Cyt^a^^,^^b^
71	*SlGSTL*4	Solyc12g044520.1	+	38363932–38367711	3780	720	720	10	239	27.84	5.52	Cy^a^,Nu^b^
72	*SlGSTL*5	Solyc12g044530.1	+	38333191–38336935	3744	789	789	9	262	30.36	6.84	Cy^a^,Mt^a^,Cp^b^
73	*SlGSTL*6	Solyc00g007030.1	-	6622946–6626691	3746	720	720	10	239	27.98	5.73	Cy^a^,Nu^b^
74	*SlGSTL*7	Solyc00g007040.1	-	6638888–6641862	2975	708	708	8	235	27.76	8.86	Cy^a^^,^^b^,Mt^a^
75	*SlGSTZ*1	Solyc01g091330.2	-	84976224–84982429	6206	1195	855	10	284	32.14	6.97	Cy^a^,Cp^b^
76	*SlGSTZ*2	Solyc01g102660.2	+	91405596–91410773	5178	1262	669	11	222	25.27	5.45	Cy^a^^,^^b^
77	*SlDHAR*1	Solyc05g013950.1	-	7428096–7430442	2346	426	426	4	141	15.85	8.55	Ec^a^,Pm^a^,Cp^b^
78	*SlDHAR*2	Solyc05g054760.2	-	64607306–64611729	4424	1162	633	6	210	23.55	6.32	Cy^a^^,^^b^
79	*SlDHAR*3	Solyc06g075520.2	+	46898478–46900198	1721	521	333	3	110	12.60	6.27	Ec^a^,Cy^b^
80	*SlDHAR*4	Solyc09g056180.2	-	47329986–47333574	3589	439	312	4	103	11.73	6.35	Ec^a^,Nu^a^,Cp^b^
81	*SlDHAR*5	Solyc11g011250.1	-	4291340–4296684	5344	807	807	6	268	29.85	8.59	Mt^a^,Cp^a^^,^^b^
82	*SlDHAR*6	Solyc11g039930.1	-	40944815–40945567	753	288	288	3	95	10.80	6.16	Ec^a^, Cp^a^^,^^b^
83	*SlEF1Bγ*1	Solyc06g011280.2	-	6257892–6260968	3077	1675	1242	8	413	47.05	5.66	Cp^a^^,^^b^
84	*SlEF1Bγ*2	Solyc11g028090.1	-	20143674–20144577	903	609	609	2	202	22.79	9.43	Nu^a^,Mt^a^,Cp^b^
85	*SlEF1Bγ*3	Solyc11g028100.1	+	20162515–20164605	2091	1245	1245	6	414	47.33	5.94	Cp^a^,Cy^b^
86	*SlTCHQD*	Solyc04g057890.2	-	55012081–55014935	2855	1125	807	3	268	31.53	9.26	Mt^a^^,^^b^,Nu^a^
87	*SlMGST*1	Solyc02g081430.2	+	45405011–45406793	1783	932	438	4	145	16.45	8.99	Pm^a^,Cp^a^
88	*SlMGST*2	Solyc04g081740.2	+	65667993–65672089	4097	1497	1158	6	386	43.48	9.14	Mt^a^, Cp^a^,Cy^b^
89	*SlGHR*1	Solyc02g068900.2	+	38779435–38783709	4275	1329	954	5	318	36.35	5.87	Mt^a^, Cy^a^,Nu^b^
90	*SlGHR*2	Solyc06g083770.2	-	49101798–49104641	2844	1543	1239	3	413	46.37	8.72	Mt^a^,Cp^a^^,^^b^^,^^c^,Nu^a^

Abbreviations: Chr, Chromosome; CDS, coding DNA Sequence; cDNA, complementary DNA; MW, Molecular Weight; pI, Isoelectric point; bp, base pair; aa, amino acid; kDa, kilodalton; Cp, Chloroplast; Ec, Extracellular; Cy, Cytoplasm; Mt, Mitochondria; Nu, Nucleus; Pm, Plasma-membrane.

^a^Localization prediction by CELLO v.2.5 (http://cello.life.nctu.edu.tw/)

^b^Localization prediction by pSORT (http://www.genscript.com/wolf-psort.html)

^c^Chloroplast localization signal confirmed by ChloroP (http://www.cbs.dtu.dk/services/ChloroP/)

### Gene duplication and Ka/Ks calculation

Gene duplication data of tomato was retrieved from plant genome duplication database (http://chibba.agtec.uga.edu/duplication/index/downloads) [34]. More than 90% sequence similarities among genes were considered as segmental duplication [35], while five or fewer genes in a 100kb region were set to separate tandem duplication. Ks (synonymous substitution rate) and Ka (nonsynonymous substitution rate) information were collected from the same database. Approximate date of the duplication event (*T* = Ks/2*λ*) was calculated for each gene pair considering a rate of 1.5×10^−8^ substitutions per site per year for dicot pants [36].

### Assessment of conserved domain and motif

The presence of conserved GST_N (PF02798.18) and GST_C (PF00043.23) -terminal domains were identified by Pfam (http://pfam.xfam.org/). Domain architecture was drawn using IBS software (Illustrator for Biological Sequences, http://ibs.biocuckoo.org/online.php) [37]. Conserved motifs were identified using the Meme program (http://meme-suite.org/index.html) [38] with statistical significance. The Meme program was run with default settings except for the maximum number of motifs were defined as 10 and the maximum width was set to 300. Functional annotation of the identified motifs was depicted using the ScanProsite program and NCBI conserved domain database [39].

### Multiple sequence alignment and phylogenetic analysis

All GST proteins from three plant species: *Solanum lycopersicum*, *Arabidopsis thaliana*, and *Oryza sativa* were retrieved from respective genome databases for phylogenetic analyses (S1 Text). Multiple sequence alignment was performed using ClustalW [40] alignment function of MEGA6 software [41]. The phylogenetic tree was constructed using Maximum-likelihood algorithm, partial deletion option and Jones-Taylor-Thornton (JTT) model with 500 bootstrap replicates to assess statistical reliability for each node.

### Expression analysis and heat map construction

Expression data of *SlGST* transcripts was retrieved from genevestigator (https://genevestigator.com/gv/) [42] at various anatomical tissues, developmental stages and in response to different biotic and abiotic stress conditions (S1 Data). Transcript abundance in microarray dataset of six developmental stages (main shoot growth, inflorescence visible, flowering, fruit formation, ripening, fruit ripening complete) and eleven anatomic tissues (root, root tip, leaf, stem, hypo-cotyledon, cotyledon, seedling, pericarp, fruit, flower and carpel) was retrieved for 30 *SlGST* genes and analyzed. Due to the absence of specific probe, transcript expression data was not available for rest of the 60 *SlGST* genes in genevestigator. Retrieved expression data was used to generate heat map using MeV 4.9 software package with hierarchical clustering method for developmental stages and anatomical tissues [43]. In case of perturbation, fold change in expression as compared to respective untreated/control sample was retrieved for each stress condition and used to generate heatmap.

### Plant stress treatment, RNA isolation and expression analysis by semiquantitative RT-PCR

Tomato seedlings were grown in a greenhouse condition with 14 h light/ 8 h dark at 26±2°C temperature. The 10 days old seedlings were exposed to various treatments (150 mM NaCl for salinity stress or 100 mM mannitol for osmotic stress or normal water for control). Seedlings were kept in normal tissue paper to depict dehydration stress. After 8 h of stress treatments, shoots were collected, weighed and total RNA was isolated using TRIzol^®^ Reagent (Thermo Fisher Scientific, USA). First-strand cDNA was synthesized using RevertAid First Strand cDNA Synthesis Kit (Thermo Fisher Scientific, USA) and semiquantitative RT-PCR was performed as described previously [44]. All gene-specific and house-keeping, *Ubiquitin* gene (*SlUBQ*, Solyc01g056940.1) primers were designed using Primer-Blast (http://www.ncbi.nlm.nih.gov/tools/primer-blast/) and synthesized from Macrogen (http://dna.macrogen.com/eng/) (S1 Table). PCR reaction was conducted in a thermal cycler (Applied Biosystem, USA) with the following program: initial denaturation of 95°C for 5 min; followed by 30 cycles of denaturation at 95°C for 30 sec, annealing at (55–60°C for 30 sec and extension at 72°C for 30 sec; and a final extension of 5 min at 72°C. Finally, amplified products were run on a 2.0% agarose gel and visualized in UV luminescence after ethidium bromide staining.

### Extraction of total protein, and measurement of GST enzyme activity

Total plant protein was extracted in native condition as described previously [45] and quantified using Bradford method [46]. The glutathione S-transferase (GST, EC 2.5.1.18) enzyme activity was measured spectrophotometrically using reduced glutathione and 1-chloro-2,4-dinitrobenzene (CDNB) substrates as published earlier [24]. The specific activity of GST (nmol/min/mg protein) was calculated and compared amongst the samples. This experiment was performed in triplicates and data was represent as the average value ± standard deviation (n = 3).

### Generation of 3D protein homology model

Homology-based model of one of the representative member, SlGSTU5 was built using I-TASSER (http://zhanglab.ccmb.med.umich.edu/I-TASSER/) [47]. I-TASSER selects template with the best identity from protein data bank hit and gives predicted model with active site residues. Discovery studio 2016 software [48] was used to visualize the predicted 3D model of SlGSTU5. Predicted 3D model was further validated with MolProbity Ramachandran analysis using PSVS (http://psvs-1_5-dev.nesg.org/) [49].

### Ligand preparation, docking grid generation and molecular docking

PDB formatted six safener structures (Fenclorim, Benoxacor, Flurazole, Dichlormid, Oxabetrinil, Fluxofenim) were generated using CambridgeSoft ChemBioOffice Ultra 2010. Docking of SlGSTU5 (receptor) with safeners (ligand) was carried out using autodock_vina_1_1_2_win32.msi [50] and MGLTools_win32_1.5.6 [51]. MGL Tools created PDBQT file of ligand and receptor was run for docking analysis to show affinity energy of ligand-receptor interaction. To visualize SlGSTU5-safener binding, discovery studio 2016 software [48] was used. Hydrogen and hydrophobic binding interactions of ligand-receptor was also visualized using this software.

### Prediction of protein–protein interaction network

Protein–protein interaction of tomato GSTs with other proteins was predicted using STRING program (http://string-db.org/) [52]. Minimum required interaction score was set to highest confidence (0.900) and max number of interactors was set not more than 50 interactors.

### Identification of putative *cis*-regulatory motifs in the promoter region

In order to analyze *cis*-acting regulatory elements in the promoter sequence of tomato *GST* genes, the 1000 bp 5’ upstream genomic DNA sequences were extracted from the Sol Genomics Network database (http://www.solgenomics.net). These sequences were subjected to the PlantCARE databases (http://bioinformatics.psb.ugent.be/webtools/plantcare/html/) [53] to find out the presence of cis-acting regulatory elements.

## Results

### Identification and characterization of *GST* gene family in *S*. *lycopersicum*

A total of 90 *GST* genes were identified in *S*. *lycopersicum* based on BLAST searches against the Sol Genomics Network (SGN) database. To classify GST members, all corresponding protein sequences were retrieved from SGN and analyzed through NCBI conserved domain database. This analysis classified 90 GST proteins into ten classes: tau (57 members), phi (6 members), theta (4 members), lambda (7 members), zeta (2 members), dehydroascorbate reductase (6 members), γ-subunit of translation elongation factor-1B (3 members), tetrachlorohydroquinone dehalogenase (1 member), microsomal GST (2 members) and glutathionyl-hydroquinone reductase (2 members). Alternative splicing is fictional as the number of *SlGST* genes is exactly equal to the number of proteins (Table 1). The length of gene and coding DNA sequence vary from 333 bp (*SlGSTU*57) to 6206 bp (*SlGSTZ*1) and 237 bp (*SlGSTU*41) to 1245 bp (*SlEF1Bγ*3), respectively. Consequently, SlEF1Bγ3 encodes the largest protein of the family with 414 amino acids in length and molecular weight of 47.33 kDa; while the smallest protein SlGSTU41 is 78 aa in length with 9.11 kDa in weight. Like CDS, protein sequence and molecular weight SlGST shows a wide variation in their isoelectric point (pI) ranging from 4.67 (SlGSTU4) to 9.43 (SlEF1Bγ2) where 77 are acidic and 13 are basic; ensures the presence of both positively and negatively charged proteins at a certain physiological condition. Most of the SlGST proteins are localized in cytoplasm followed by chloroplast, extracellular, mitochondria, nucleus, and plasma-membrane (Table 1).

### Chromosomal localization and gene duplication

**Fig 1:**
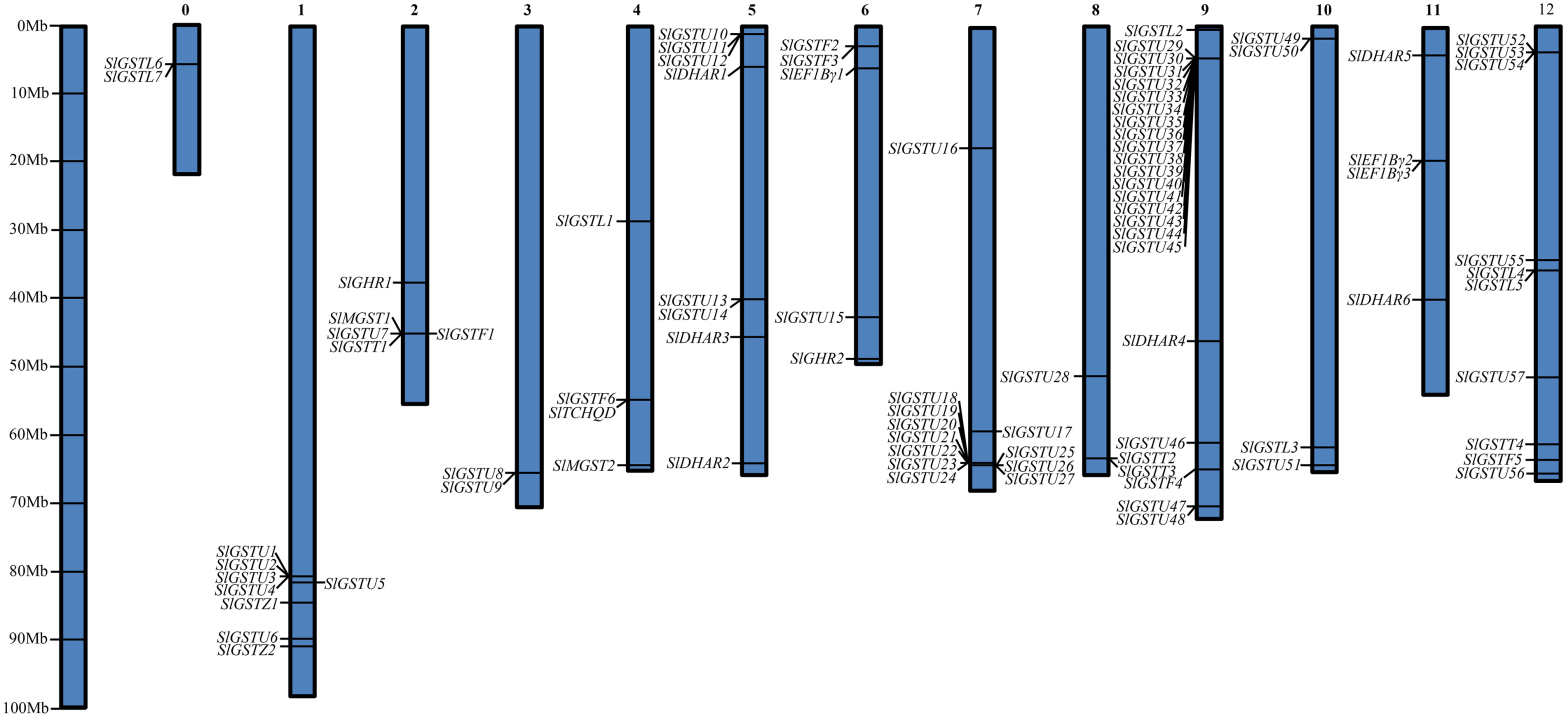
Chromosomal localization of 90 *GST* genes on 13 chromosomes of tomato. The chromosome numbers are indicated on top of chromosomes, and size of the chromosome is represented using a vertical scale (Mb). Chromosomal positions of the *SlGST* genes are indicated by exact name and could be inferred from the provided scale.

All the *SIGST* gene loci (90 in number) are found to be unevenly distributed across the 13 different chromosomes, ranging from 2 to 23 genes per chromosome. A maximum 23 genes is located on chromosome 9, followed by 12 genes on chromosome 7. In contrast, only two genes each is located on chromosome number 0 and 3 (Fig 1). Seventeen gene clusters are distributed on thirteen different chromosomes where 10 gene clusters formed by 45 genes of tau class alone and rests are formed from mixed classes. A total of 8 segmental duplication events- *SlGSTU*16/ *SlGSTU*55, *SlGSTU*18/ *SlGSTU*52, *SlGSTU*19/ *SlGSTU*53, *SlGSTU*19/ *SlGSTU*50, *SlGSTU*29/ *SlGSTU*51, *SlGSTU*46/ *SlGSTU*47, *SlGSTU*49/ *SlGSTU*52 and *SlGSTU*56/ *SlGSTU*47 are detected in *SlGST* family but lack of tandem duplication (Table 2). A maximum number of five duplicated *GST* genes were located on chromosome 12, four on chromosome 7, three each on chromosome 9 and 10 (Table 2). Ka and Ks ratio was used to investigate the selective constraints on duplicated GST genes where Ka/Ks ratio >1 implies positive selection, Ka/Ks = 1 implies neutral selection, while a ratio <1 indicates negative or purifying selection. All duplicated *GST* genes in tomato showed Ka/Ks ratio less than 1, which implies the influence of purifying selection in the evolution of these gene pairs. The segmental duplications of *GST* genes in tomato originated from 5.0 Mya (Ks = 0.15) to 50.9 Mya (Ks = 1.53), with the mean being 35.0 Mya (Ks = 1.05).

**Table 2:** Duplicated *GST* genes and the probable dates of duplication blocks in tomato.

Duplicated gene 1	Duplicated gene 2	Ka	Ks	Ka/Ks	Duplication time (Mya)	Purifying selection	Duplicate type
*SlGSTU*16	*SlGSTU*55	0.11	0.1518	0.724	5.0	Yes	Segmental
*SlGSTU*18	*SlGSTU*52	0.2484	1.4728	0.168	49.0	Yes	Segmental
*SlGSTU*19	*SlGSTU*53	0.1946	0.9335	0.208	31.1	Yes	Segmental
*SlGSTU*19	*SlGSTU*50	0.1626	1.5282	0.106	50.9	Yes	Segmental
*SlGSTU*29	*SlGSTU*51	0.2343	1.1915	0.196	39.7	Yes	Segmental
*SlGSTU*46	*SlGSTU*47	0.3776	1.1734	0.321	39.1	Yes	Segmental
*SlGSTU*49	*SlGSTU*52	0.3138	0.83	0.378	27.7	Yes	Segmental
*SlGSTU*56	*SlGSTU*47	0.3728	1.1389	0.327	38.0	Yes	Segmental

### Structure of *SlGST* transcripts

**Fig 2:**
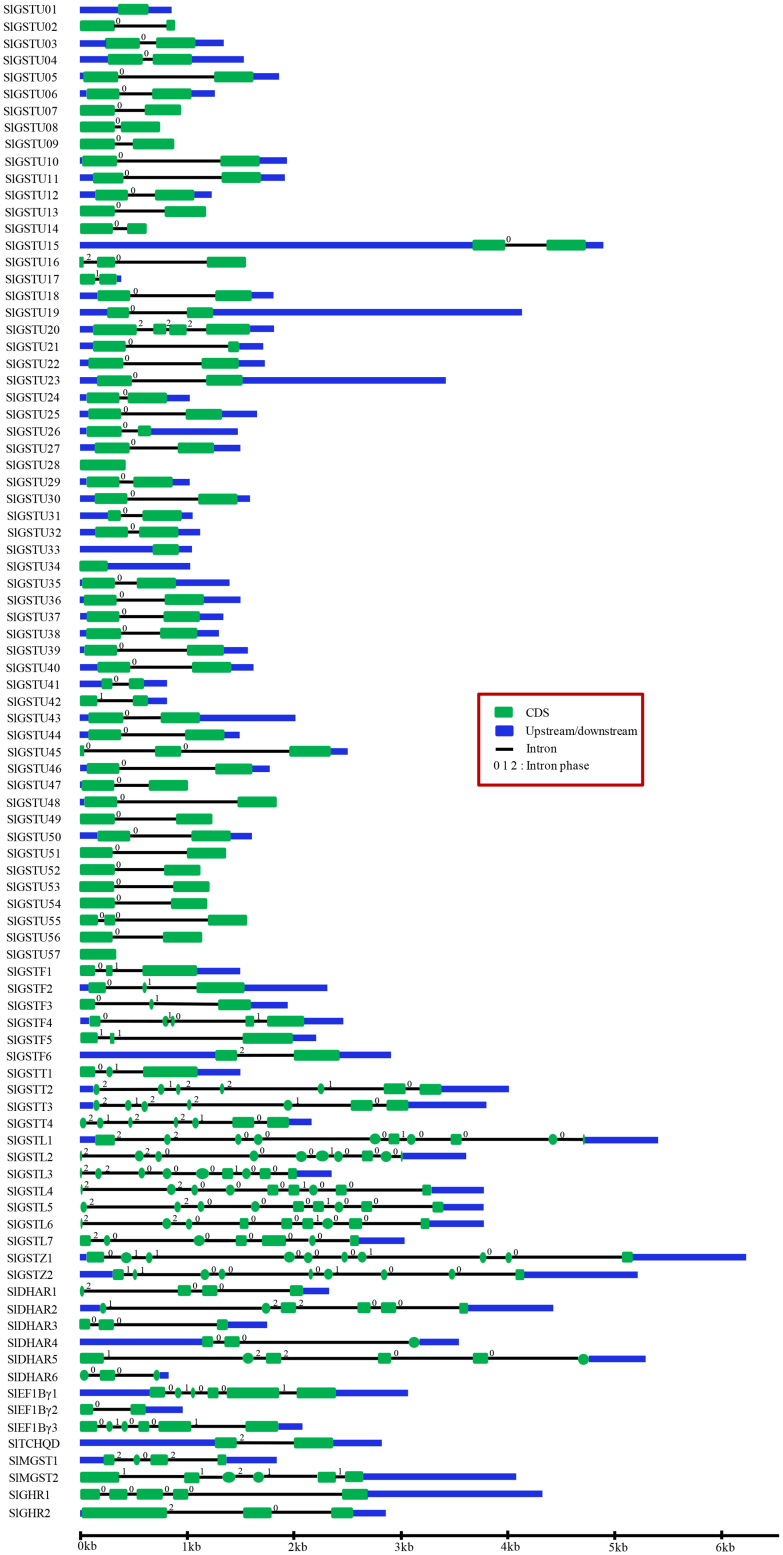
Exon-intron structures of all *SlGST* genes. Schematic diagram represents the gene structure of all 90 *SlGST* genes identified in this study using Gene Structure Display Server (http://gsds1.cbi.pku.edu.cn/). Exons are shown as green boxes; introns are shown as black lines; and upstream/downstream are shown as blue boxes. 0 indicates an intron located between two consecutive codons, 1 indicates splitting codons between the first and second nucleotides, and 2 indicate an intron inserted into the second base of a codon. The relative size of the full transcript, intron, and exon could be inferred from the scale provided below in kilo base pair, kb.

Structural analysis of *SlGST* genes were featured by comparing exon-intron position and turned out with great variation among themselves (Fig 2). The number of exons varies from 1 to 10 with maximum number of exons in *SlGSTL*1, *SlGSTL*2, and *SlGSTZ*1 (10 exons), and minimum number of exon is found in *SlGSTU*1, *SlGSTU*28, *SlGSTU*33, *SlGSTU*34 and *SlGSTU*57 with only 1 exon in their gene structure. However, 2 exons present in 51 members, followed by 3 exons in 12 members, 4 exons in 3 members, 5 exons in 2 member, 6 exons in 5 members, 7 exons in 4 members and 9 exons in 5 members (Fig 2). Consequently, the number of introns varies from 0 to 9 in the ORFs in different *SlGST* transcripts. *SlGSTU*1, *SlGSTU*28, *SlGSTU*33, *SlGSTU*34, and *SlGSTU*57 are lack of intron in their gene structure. On contrary, only single intron is present in 51 members, followed by 2 introns in 12 members, 3 introns in 3 members, 4 introns in 2 members, 5 introns in 5 members, 6 introns in 4 members, 8 introns in 5 members and 9 introns in 3 members (Fig 2). Intron phase is associated with the conservation of splicing site and related to the evolution of spliceosome machinery [54]. Intron phase 0 shows the highest conservation, while intron phase 2 shows the lowest conservation and phase 1 is intermediate. Tau class members showed the highest conservation with maximum intron phase 0, whereas theta, zeta, lambda, dehydroascorbate reductase classes showed greater intron numbers with mixed conservation of splice site sequence (Fig 2).

### Conserved domain and motif analysis

**Fig 3:**
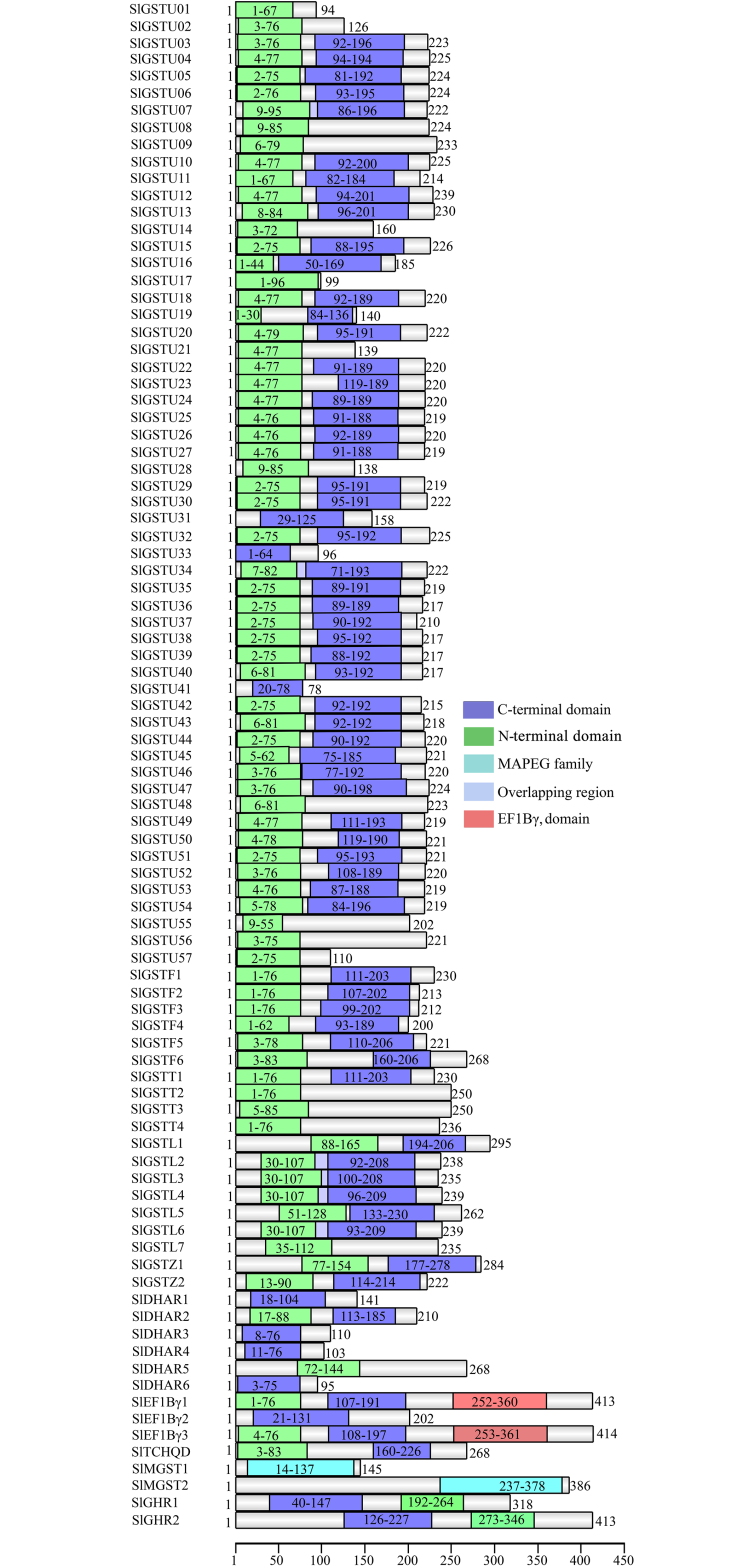
Schematic representation of domain structures of SlGST proteins. All SlGST proteins were analyzed to identify the presence of conserved domains. Different identified domains, such as GST_N domain (PF02798.18), GST_C domain (PF00043.23), microsomal GST domain (PF01124.16), domain overlapping regions and EF1Bγ domain (PF00736) are shown by green, royal blue, anaqua, light blue and red boxes, respectively. Domain position is indicated by the exact amino acid number inside the box. The length of full protein is indicated by an exact amino acid number and the relative position of the GST domains could be interpreted by the scale (amino acid) at the bottom of the figure.

To identify the presence of conserved domains in each SlGST proteins, protein sequences were analyzed through Pfam. This analysis showed that SlGSTU1, SlGSTU8, SlGSTU9, SlGSTU17, SlGSTU21, SlGSTU28, SlGSTU48, SlGSTU55, SlGSTU56, SlGSTU57, SlGSTT1, SlGSTT2, SlGSTT3, SlGSTL7, SlDHAR5 proteins had only N-terminal GST domain; and SlGSTU31, SlGSTU33, SlGSTU41, SlDHAR3, SlDHAR4, SlDHAR6, SlEF1Bγ2 proteins had only C-terminal GST domain in their protein structure (Fig 3). Rest of the SlGST proteins contain both N-terminal and C-terminal GST domain except SlMGST which contain MAPEG domain in its structure. SlEF1B*γ*1 and SlEF1B*γ*3 have an additional EF1Bγ domain (PF00736) in their protein structure. Putative conserved motifs of SlGST proteins were identified using Meme motif search analysis. Ten highly conserved motifs with more than 10 amino acids in length were identified (S2 Table). Motif- 2, 4, 5, 6 and 10 were found to be specific for tau family members only; motif- 7 is present in both tau and DHAR classes; motif-8 is specific for lambda class; motif-9 is present in most of the phi, theta, zeta, EF1Bγ, TCHQD members. Motifs- 1, 3, 8 and 9 belong to the N-terminal GST domain region, while motifs- 2, 4, 5, 6 and 7 are present at the C-terminal GST domain.

### Analysis of evolutionary relationship between GST proteins of tomato, rice, and *Arabidopsis*

**Fig 4:**
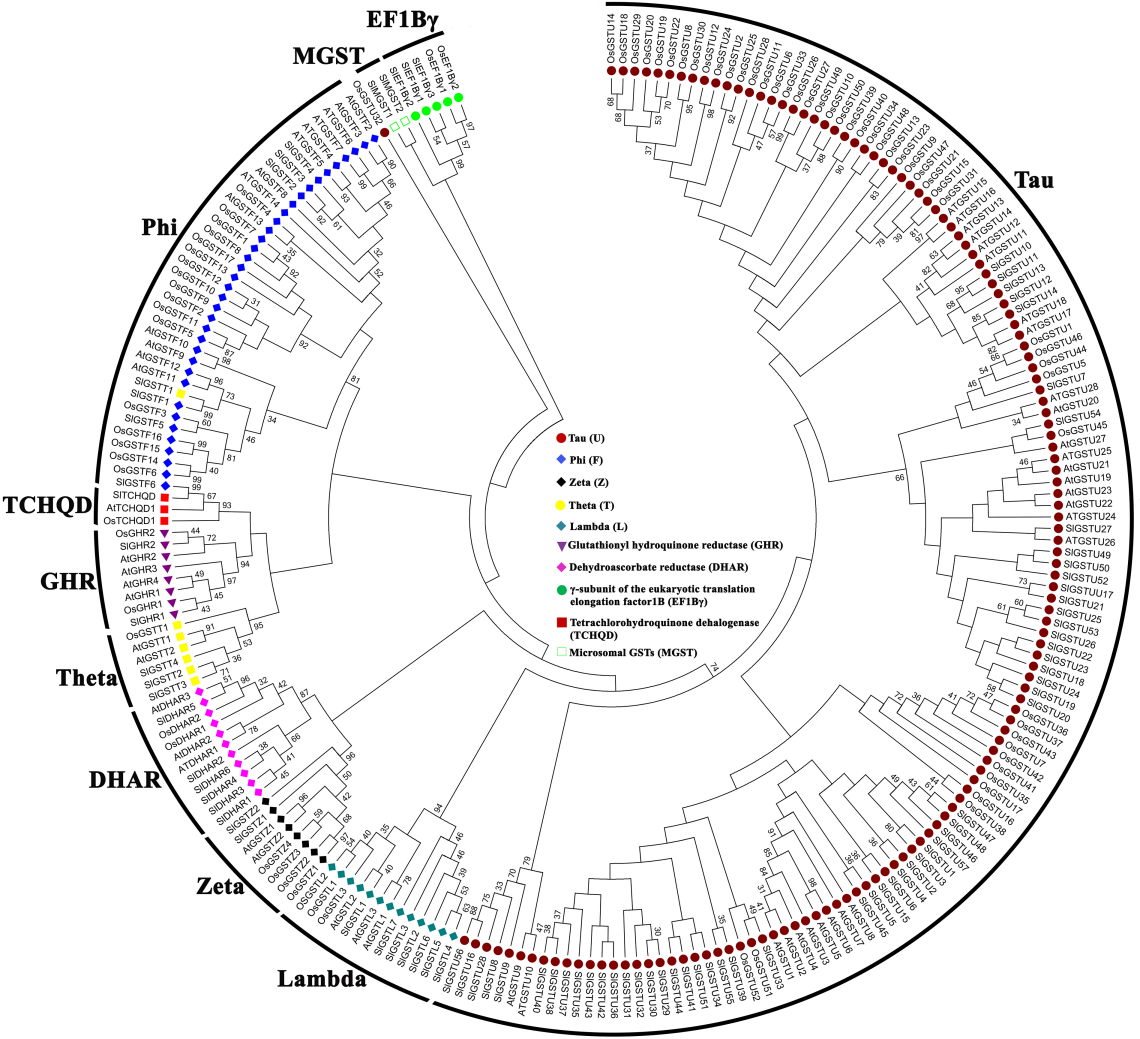
Phylogenetic relationship among the GST proteins of tomato, *Arabidopsis*, and rice. The unrooted phylogenetic tree was constructed from a complete alignment of 228 GST proteins from three plant species using MEGA 6.0 Maximum-likelihood method with 500 replicate bootstrap analysis. Percentage bootstrap scores of greater than 30% were shown in the tree. Each class of soluble GST and microsomal GST is shaded in different colors. Different members of tau, lambda, zeta, DHAR, theta, GHR, TCHQD, phi, MGST, EF1Bγ classes were marked with brown circle, sea green diamond, black diamond, pink diamond, yellow rectangle, orchid triangle, red rectangle, blue diamond, empty rectangle and green circle; respectively. The name of corresponding proteins from tomato, *Arabidopsis* and rice proteins were indicated at the end of each branch.

To explore the expansion of GST family members in tomato vs rice and *Arabidopsis*, an unrooted phylogenetic tree was generated (Fig 4, S1 Text). The phylogenetic tree showed that each class of GST protein family members from these species clustered together to form separate clade except SlGSTT1 and OsGSTU32 (Fig 4). This indicates that the separation of GST classes took place before the divergence of monocots and dicots, and individual family members increase later in a species-specific gene expansion manner. Phylogenetic analysis showed that tau is the largest sub-class of plant GST family in tomato, rice and *Arabidopsis* comprised of 57, 52 and 28 members, respectively. The second largest plant GST class is phi containing 6, 17, and 14 members in tomato, rice and *Arabidopsis*, respectively. Consequently, the third largest plant GST class is lambda with 7 SlGST, 3 AtGST and 3 OsGST members. Similarly, tomato has 6 DHAR GST, while *Arabidopsis* has 3 and rice has 2 members. Significantly, the number of members in each classes of GST is higher in tomato as compared to rice and *Arabidopsis*. Tau and lambda are the largest class of GST in tomato, whereas tau and phi are the largest in rice and *Arabidopsis*.

### Expression pattern of different *SlGST* genes at various developmental stages and tissues

**Fig 5:**
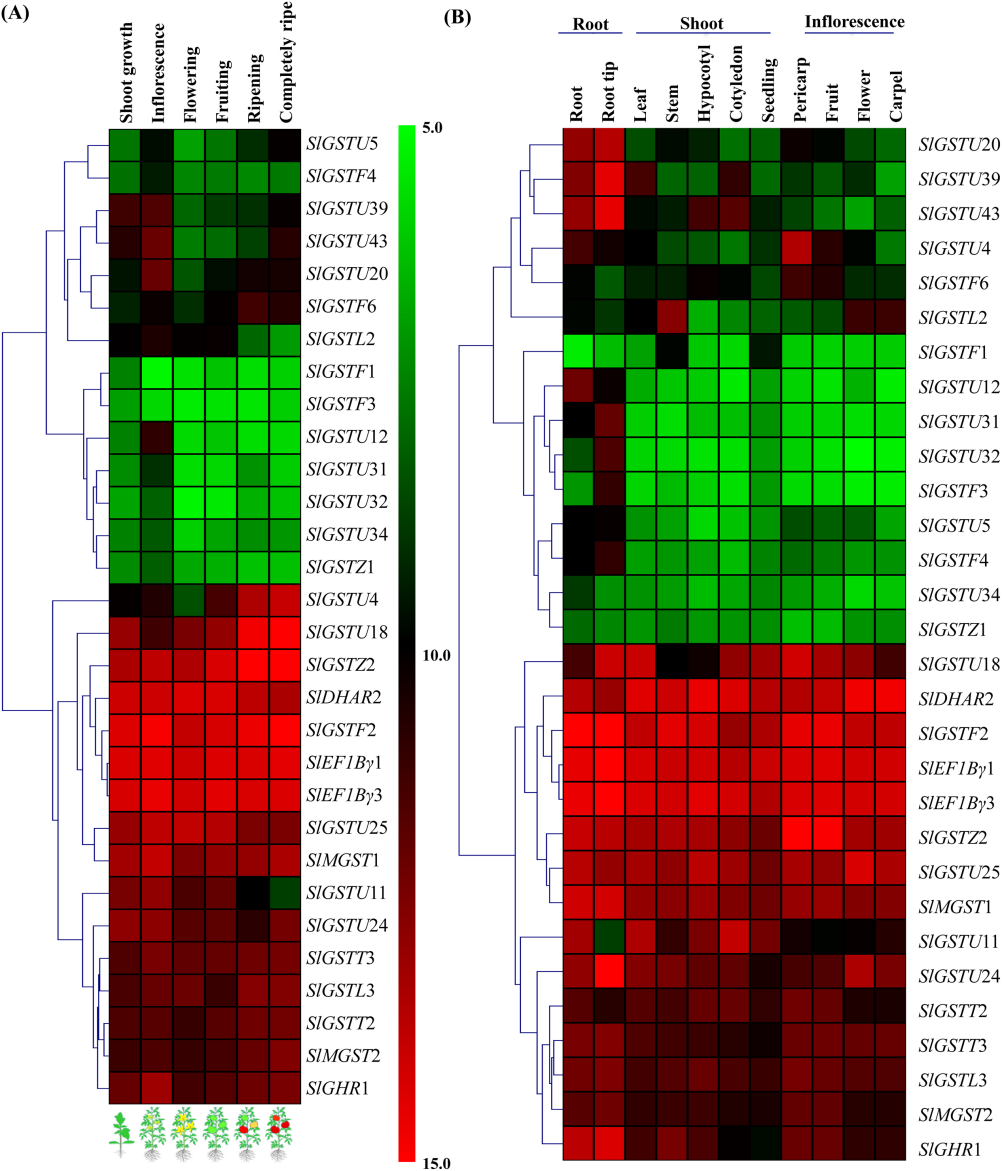
Expression profile of *SlGST* genes at various anatomical tissues and developmental stages. Microarray expression data corresponding to 30 *SlGST* genes was retrieved from genevestigator database for further analysis. Six distinguished developmental stages such as main shoot growth, inflorescence visible, flowering, fruit formation, ripening, fruit ripening complete were used in the study. Eleven anatomical tissues are divided into three major parts- root (root and root tip), shoot (leaf, stem, hypo-cotyledon, cotyledon, seedling), and inflorescence (pericarp, fruit, flower, and carpel). Hierarchical clustering of the expression profile was done with log_2_ transformed average values using MeV software package. The color scale provided by a vertical scale at the middle of two heat map represents the log_2_ intensity value. High level of expression is shown as red color and the low level is present as green color.

Expression of *SlGST* genes was analyzed at different developmental stages and anatomical tissues using microarray data available in genevestigator. All these analyzed 30 *SlGST* genes formed two distinct clades in their expression pattern at different developmental stages (Fig 5). One set of genes displayed very high-level of expression throughout the entire life at different developmental stages, while another set showed a low level of expression. Among them, *SlGSTZ*2, *SlDHAR*2, *SlGSTF*2, *SlEF1Bγ*1, and *SlEF1Bγ*3 maintained the highest level of expression; while SlGSTF1 and SlGSTF3 showed the lowest level of expression (Fig 5A). Interestingly, expression of *SlGSTU*20 and *SlGSTF*6 transcripts were found to increase at the fruit ripening stage indicating their involvement in fruit ripening process (Fig 5A). Similar to developmental stages, expression pattern could be divided into two clear parts at various tissues (Fig 5B). *SlDHAR*2, *SlGSTF*2, *SlEF1Bγ*1, and *SlEF1Bγ*3 transcripts maintained the highest level at all the analyzed tissues; while *SlGSTU*34 and *SlGSTZ*1 partaken the lowest level of expression (Fig 5B). Tissue-specific alteration of transcript abundance was also observed, such as *SlGSTU*20, *SlGSTU*29, and *SlGSTU*43 showed highly root specific expression.

### Expression analysis of *SlGST* genes under different biotic and abiotic stresses

**Fig 6:**
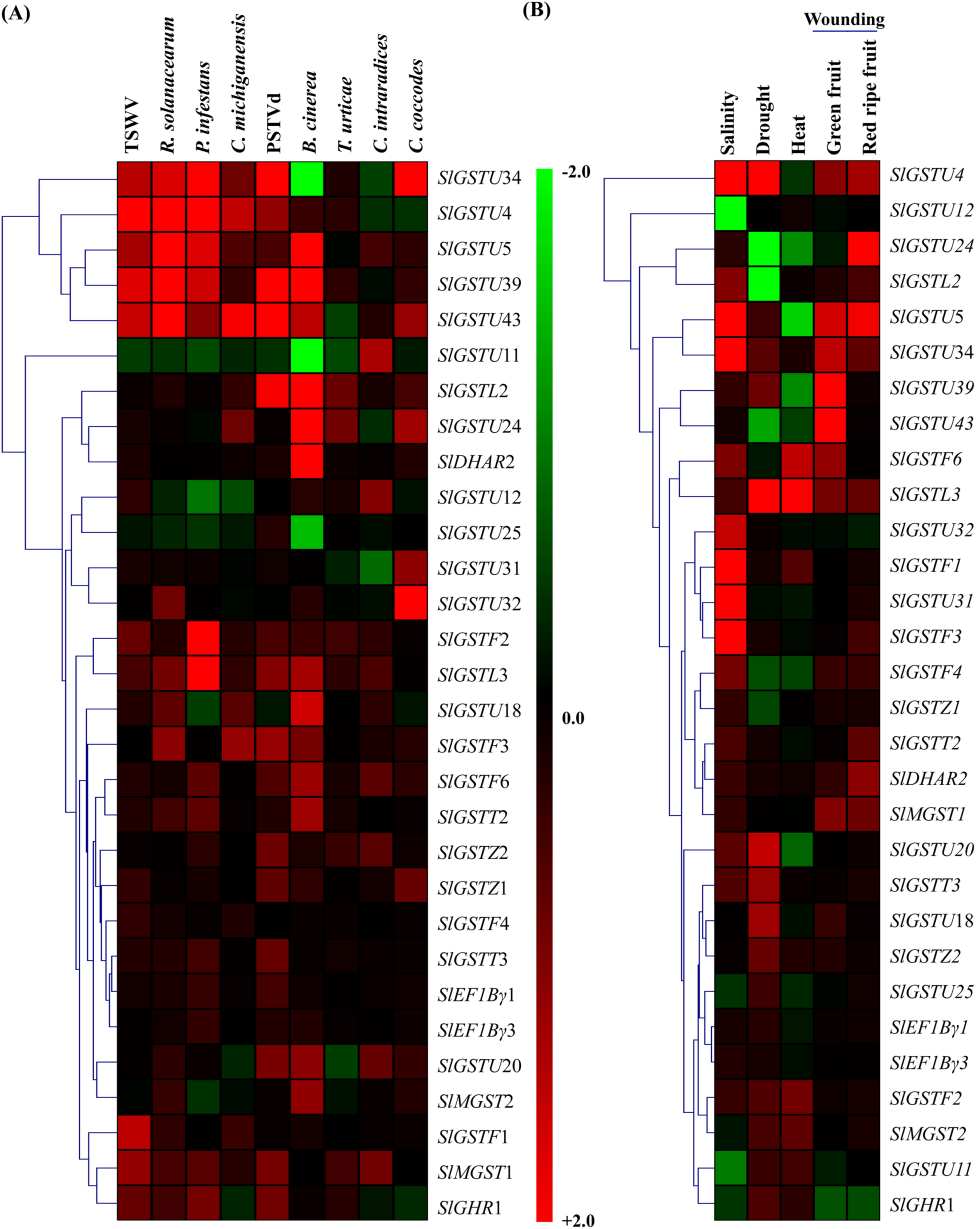
Expression pattern of *SlGST* genes in various biotic and abiotic stresses. Expression data of 30 *SlGST* genes were retrieved from genevestigator at various unfavorable conditions. Transcript abundance of *SlGST* genes was retrieved in response to (A) nine pathogens- TSWV, *R*. *solanacearum*, *P*. *infestans*, *C*. *michiganensis*, PSTVd, *B*. *cinera*, *T*. *urticea*, *C*. *intraradices* and *C*. *coccodes*; and (B) five abiotic stress conditions and relative fold change in expression as compared to control was used to generate heatmap with hierarchical clustering by MeV software package. The color scale, depicted at the middle, represents the intensity of alterations. Up-regulation and down-regulation are shown by red and green color, respectively.

Relative transcript abundance dataset of the same 30 *SlGST* members were retrieved and analyzed in response to nine pathogens- Tomato spotted wilt virus (TSWV), *R*. *solanacearum*, *P*. *infestans*, *C*. *michiganensis*, Potato spindle tuber viroid (PSTVd), *B*. *cinera*, *T*. *urticea*, *C*. *intraradices* and *C*. *coccodes*; and five abiotic stress conditions- salinity, drought, heat, wounding at green mature fruit stage and red ripe fruit stage (Fig 6). In response to pathogens, most of the analyzed *SlGST* members showed strong up-regulation with few exceptions like *SlGSTU*34, *SlGSTU*11 and *SlGSTU*25 showed significant down-regulation after *B*. *cinera* infection (Fig 6A). A cluster of genes *SlGSTU*34, *SlGSTU*4, *SlGSTU*5, *SlGSTU*39 and *SlGSTU*43 exhibited a strong up-regulation in response to multiple pathogens- TSWV, *R*. *solanacearum*, *P*. *infestans*, *C*. *michiganensis*, and PSTVd infection (Fig 6A). These five members might be the key players to modulate against biotic stress. Most of the *SlGST* transcripts showed strong up-regulation in response to four different abiotic stresses, too with few exceptions (Fig 6B). In response to salt stress *SlGSTU*4, *SlGSTU*5, *SlGSTU*34, *SlGSTF*1, *SlGSTU*31, *SlGSTU*32 and *SlGSTF*3 showed sharp up-regulation; while *SlGSTU*12 and *SlGSTU*11 were drastically down-regulated (Fig 6B). Similarly, drought stress stimulated sharp up-regulation in *SlGSTU*4, *SlGSTL*3 and *SlGSTU*20 transcripts and down-regulation in *SlGSTU*24, *SlGSTL*2 and SlGSTU43 transcripts. In response to wounding at both green and red fruit stages, *SlGSTU*4, *SlGSTU*5, *SlGSTU*34, and *SlGSTL*3 showed up-regulation and the rests remain nearly unchanged (Fig 6B). Among all the 30 analyzed *SlGST* genes *SlGSTU*4, *SlGSTU*5, *SlGSTU*34, *SlGSTF*6 and *SlGSTL*3 are found to be up-regulated maximum in response to four different abiotic stresses. Taken together both biotic and abiotic stresses, *SlGSTU*4, *SlGSTU*5, and *SlGSTU*34 are appeared to be the key stress responsive members.

### Expression of *SlGST* transcripts altered in response to various abiotic stresses

**Fig 7:**
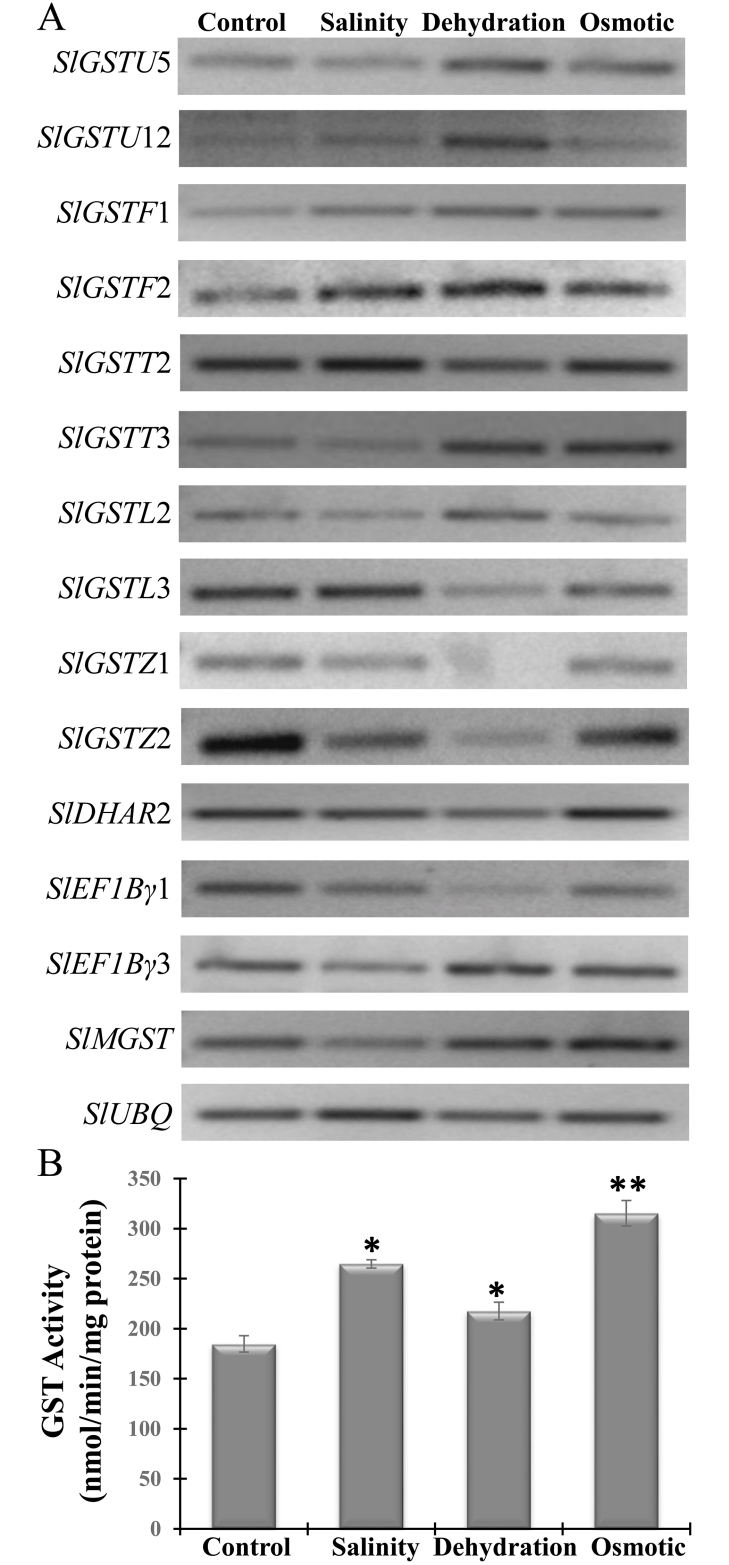
Expression analysis of selected *SlGST* genes and measurement of total GST activity in response to various abiotic stresses. (A) Semiquantitative RT-PCR analysis of fourteen selected *SlGST* genes was performed in response to salinity, dehydration and osmotic stresses. Gene-specific primers with an amplicon size of 100–200 bp were used to analyze their level of expression by PCR as compared to their control level. Ubiquitin (*SlUBQ*) gene used as an internal control to minimize intra-sample variation. (B) Total tomato GST enzyme activity was measured in response to the same three stresses. The activity was represented as nmol/min/mg protein. All the experiments were repeated thrice and represent as the average ±standard deviation (n = 3). *, ** represents the significance level of paired student’s two-tailed t-test with a p-value less than 0.05 and 0.01; respectively.

Expression of *SlGST* genes was further validated by semiquantitative RT-PCR in response to three devastating abiotic stresses- salinity, dehydration and osmotic stress (Fig 7A). Expression of fourteen *SlGST* transcripts was analyzed along with house-keeping gene, *ubiquitin* that acts as an experimental control (Fig 7A). Several *SlGST* transcripts showed dynamic stress specific pattern of expression, indicating towards the specific role of every member in different type of stress. RT-PCR analysis suggested that *SlGSTF*1 and *SlGSTF*2 transcript showed significant up-regulation in response to all three- salinity, dehydration and osmotic stresses, as compared to their respective control (Fig 7A). Similarly, *SlGSTU*5, *SlGSTT*3, and *SlMGST* showed up-regulation in response to dehydration and osmotic stresses, while slightly down-regulated against salinity stress. On contrary, *SlGSTZ*1, and *SlGSTZ*2 showed significant down-regulation in response to all three stresses analyzed in the study. However, expression of some *SlGST* members (*SlDHAR*1 and *SlEF1Bγ*3) showed slight alteration in response to these stresses (Fig 7A). Overall, osmotic stress exhibited a salient positive modulation on *SlGST* gene family. However, down-regulation of *SlGSTU*12 and *SlGSTL*3 transcript was only restricted to salinity and dehydration stress, respectively. It can be speculated that different members might involve in different intercellular mechanism to minimize the stress damage. The expression analysis clearly identified several *SlGST*s were highly up/down-regulated in a stress-specific manner. Further functional analyses on SlGSTs are now necessary to understand the roles of individual members of *SlGST* family.

### Enhanced total GST enzyme activity in response to salinity, dehydration, and osmotic stresses

As most of the analyzed *SlGST* members showed strong up-regulation in response to various biotic and abiotic stresses (Figs 6 and 7A), it could be expected that total tomato GST enzyme activity might increase under unfavorable conditions. To assess the positive relation of transcript abundance with corresponding protein activity, total tomato GST activity was measured in response to the same three abiotic stresses such as salinity, dehydration and osmotic stresses. GST enzyme activity enhanced significantly in response to all three stresses as compared to untreated control sample (Fig 7B). Among three stresses, osmotic stress showed maximum induction of total GST activity that is in harmony with the strong up-regulation of several *SlGST* members at transcript level (Figs 6B and 7A). However, the level of stimulation was found to be almost similar in case of both salinity and dehydration stresses indicating towards their related nature of stress effects.

### Identification of positive modulators for tomato GST activity

**Fig 8:**
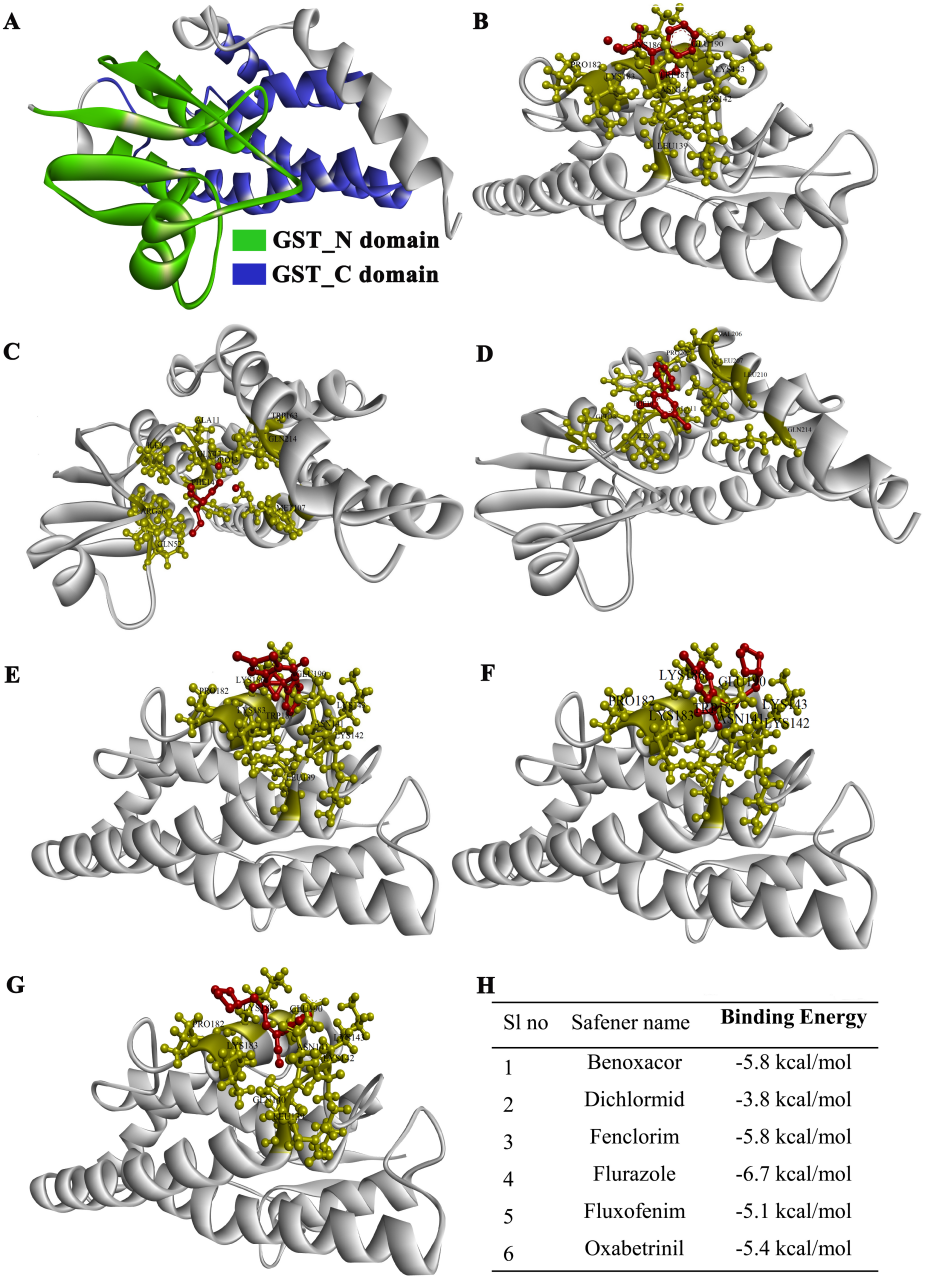
Molecular docking study of SlGSTU5 with safeners. (A) The structure of SlGSTU5 was built using I-TASSER server based on available close similar structure from Protein Data Bank (PDB). GST_N domain and GST_C domain were marked with green and blue color, respectively. (B-G) Diagrams represent the interaction (B) Benoxacor, (C) Dichlormid, (D) Fenclorim, (E) Flurazole, (F) Fluxofenim, and (G) Oxabetrinil with SlGSTU5 protein. Safeners were indicated by dark red color and binding residues of SlGSTU5 were shown by gold color. (H) The corresponding binding energy (kcal/mol) of all these safeners with SlGSTU5 protein.

As GST activity is found to be increased in response to all stresses, enhancement of *in vivo* GST activity would be a good tactic to raise stress resistant plant. For this, a homology-based model of highly stress responsive SlGSTU5 (Fig 8A) was generated using one of the soybean GSTU (4TOP) as a template and exported from I-TASSER with a confidence score (C-score) of 0.99. The generated model has an estimated TM-score and RMSD value of 0.85±0.08 and 3.6±2.5 Å, respectively. Further, 3D model of SlGSTU5 was validated using MolProbity Ramachandran analysis, which showed 90.5% (201/222) of all residues were in favored (98%) regions and 97.7% (217/222) of all residues were in allowed (>99.8%) regions (S1 Fig). To identify the positive modulators of SlGSTU5, 3D model of SlGSTU5 was docked with six well-known safener ligands (S2 Fig) that have been reported previously for the enhancement of GST transcripts in other plant species [18]. The center of the grid map was X (96), Y (44) and Z (74); and the autogrid calculation was set as 59.212× 59.179× 59.227 Å with the active site residues at the center of the grid box. Six different safeners- Fenclorim, Benoxacor, Flurazole, Dichlormid, Oxabetrinil, and Fluxofenim binds differently with SlGSTU5 (Fig 8B–8G). They have a binding energy of -5.8 kcal/mol, -5.8 kcal/mol, -6.7 kcal/mol, -3.8 kcal/mol, -5.4 kcal/mol, -5.1 kcal/mol; respectively (Fig 8H). Residues of SlGSTU5 that are involved in the binding are marked and the resulted interaction was basically through hydrogen bond and hydrophobic interactions (S3 Fig). Out of these six safeners, flurazole binds with the lowest affinity energy (Fig 8H) and could be effectively applied to enhance *in vivo* GST activity of tomato.

### Protein–protein interaction network prediction for SlGST proteins

**Fig 9:**
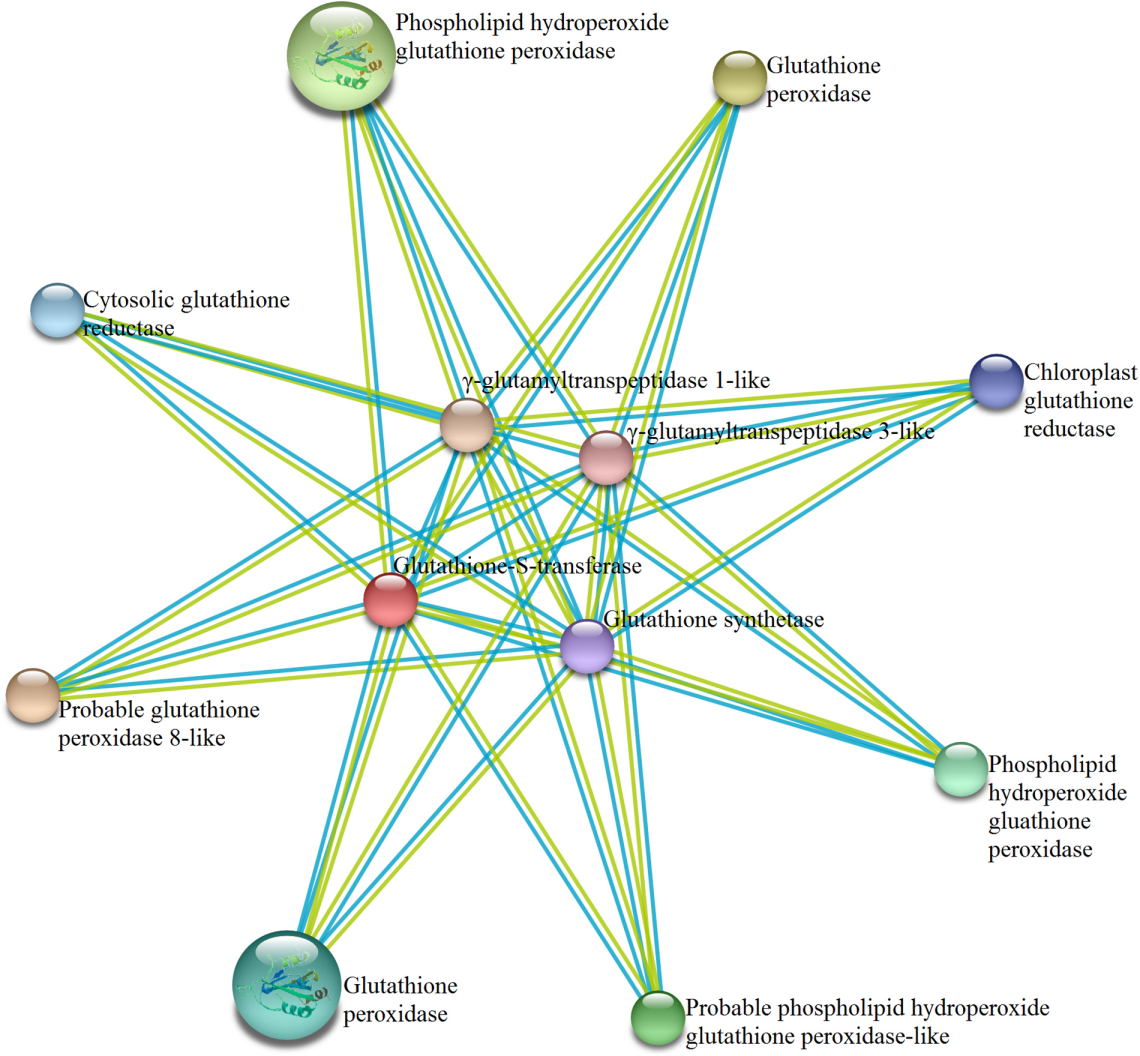
The interaction network of SlGST proteins with other proteins. According to STRING functional protein association networks server, the protein-protein interaction of SlGST was predicted. Here, small nodes in the figure represent unknown 3D structure whereas large nodes indicate the availability of known or predicted 3D structure, and red colored node represent the query proteins. The edge with turquoise and yellow color indicate the prediction was made based on curated databases and text-mining, respectively.

As *SlGST* transcripts and proteomes showed high-stress responsiveness (Fig 7), SlGST proteins might interact with other proteins to modulate the effects of stress. A total of eleven proteins were predicted to interact with SlGST proteins with a high confidence score of more than 0.9. The network map (Fig 9) showed the interaction of GST with other tomato proteins according to STRING database analysis. Tomato GST proteins are predicted to interact with glutathione peroxidase, phospholipid hydroperoxide glutathione peroxidase, phospholipid hydroperoxide glutathione peroxidase-like, glutathione peroxidase 8-like, chloroplast and cytosolic glutathione reductase, glutathione synthase, γ-glutamyl transpeptidase 3-like, γ-glutamyl transpeptidase 1-like proteins (Fig 9). The interaction map contains a total 12 nodes with 66 edges. Further analysis of the interacting members deciphers that all these proteins are involved in glutathione biosynthesis and utilizing pathways. Thus, GSTs might have a dynamic interaction with other glutathione dependent enzymes to regulate cellular normal physiology.

### Identification of cis-regulatory elements in the promoter region of *SlGST* genes

The 5’-upstream promoter (1 kb) region of 30 *SlGST* genes were analyzed using PlantCARE to identify the presence of stress specific cis-elements. Several stress-responsive cis-elements such as abscisic acid responsive element (ABRE), auxin-responsive element (AuxRR-core), fungal elicitor-responsive element (BOX-W1), ethylene responsive element (ERE), gibberellin-responsive element (GARE), heat shock element (HSE), low temperature responsive element (LTR), MYB-binding site (MBS), defense and stress responsive element (TC-rich), wounding and pathogen-responsive elements (W-box motif), salicylic acid-responsive element (TCA), Methyl jasmonate-responsive element (CGTCA box and TGACG motif), element conferring high transcription level (5’ UTR Py-rich stretch) were found to be present in the promoter of *SlGST* genes (Table 3). Maximum numbers of 10 cis-elements were located in the promoter of *SlGSTF1* and a minimum number of 1 cis-element present in *SlGSTU1*. However, defense and stress responsive element (TC-rich) element is found to be present maximum 34 times, followed by ABRE motifs with 23 times and HSE motifs with 21 times (Table 3). The presence of these highly stress-inducible elements in the *SlGST* promoter sequences could be directly correlated with the stress-inducible up-regulation of *SlGST* transcripts (Figs 6 and 7A) and total SlGST enzyme activity (Fig 7B).

**Table 3:** Numbers of known stress-related cis-regulatory elements present in the upstream region of *SlGST* genes.

Sl no	Motifs Gene name	ABRE	AuxRR-core	Box-W1	ERE	GARE	HSE	LTR	MBS	TC rich repeat	TCAelement	TGACG motif	5’-UTR py-rich stretch	Total
		TACGTG	GGTCCAT	TTGACC	ATTTCAAA	AAACAGA	AAAAAATTTC	CCGAAA	TAACTG	ATTCTCTAAC	GAGAAGAATA	TGACG	TTTCTTCTCT	
1	*SlGSTU*4	2	-	1	-	-	1	-	2	1	1	-	-	**8**
2	*SlGSTU*5	-	-	1	1	-	2	1	-	-	-	-	-	**5**
3	*SlGSTU*11	-	-	-	-	-	-	-	1	-	-	-	-	**1**
4	*SlGSTU*12	1	-	-	-	3	-	-	-	-	-	-	-	**4**
5	*SlGSTU*17	-	-	-	-	-	1	-	-	1	3	-	-	**5**
6	*SlGSTU*19	1	-	-	-	1	1	-	-	1	-	2	1	**7**
7	*SlGSTU*23	2	-	-	1	2	1	-	-	2	-	1	-	**9**
8	*SlGSTU*24	2	1	-	-	-	-	-	-	2	-	2	-	**7**
9	*SlGSTU*30	1	-	1	-	-	2	-	-	2	1	-	1	**8**
10	*SlGSTU*31	1	-	-	-	-	1	-	-	1	2	1	-	**6**
11	*SlGSTU*32	1	-	1	1	-	-	1	2	-	-	-	-	**6**
12	*SlGSTU*37	-	-	2	1	-	-	-	1	2	-	-	1	**7**
13	*SlGSTU*40	-	-	-	-	-	2	-	1	3	-	-	-	**6**
14	*SlGSTF*1	1	-	1	-	2	-	1	1	3	1	-	-	**10**
15	*SlGSTF*2	-	-	-	-	-	1	-	-	1	-	1	1	**4**
16	*SlGSTF*3	2	-	-	-	-	-	-	-	-	1	-	-	**3**
17	*SlGSTF*4	-	-	-	-	-	-	-	2	3	1	-	-	**6**
18	*SlGSTF*6	-	-	-	-	-	1	-	-	-	-	-	3	**4**
19	*SlGSTT*2	1	-	-	-	2	-	-	1	3	1	-	-	**9**
20	*SlGSTT*3	-	-	-	-	-	-	-	1	-	-	2	-	**3**
21	*SlGSTL*2	-	-	1	1	-	1	-	-	-	-	2	-	**5**
22	*SlGSTL*3	1	-	-	-	-	-	1	1	1	-	1	1	**6**
23	*SlGSTZ*1	1	-	-	-	1	2	1	-	-	-	-	1	**6**
24	*SlGSTZ*2	-	-	-	2	1	1	-	1	1	-	-	1	**7**
25	*SlDHAR*2	-	-	-	-	-	2	-	-	2	-	-	-	**4**
26	*SlEF1Bγ*1	2	-	-	-	-	1	-	1	1	1	1	-	**7**
27	*SlEF1Bγ*3	2	-	-	-	-	1	-	1	1	1	1	-	**7**
28	*SlMGST*1	-	-	1	-	-	-	-	-	1	2	-	-	**4**
29	*SlGSTT*2	2	-	-	-	-	-	-	-	-	-	-	-	**2**
30	*SlGHR*1	-	-	-	-	2	-	1	-	2	2	1	**1**	**9**
**Total**		**23**	**1**	**9**	**7**	**14**	**21**	**6**	**16**	**34**	**17**	**15**	**11**	**174**

## Discussion

An *in silico* approach was taken to evaluate the functional diversification of *GST* gene family members in tomato and identified a total of 90 *SlGST* genes (Table 1). The number of *GST* genes in tomato is higher than other plants such as *Arabidopsis*, rice, barley, sweet orange, larch, cotton and *C*. *rubella* by 1.6 times, 1.1 times, 1.03 times, 3.8 times, 3.2 times, 1.5 times, and 1.8 times; respectively [8, 9, 19–22]. Species-specific segmental duplications event might be the possible reason behind the higher GST members in tomato as compared to other plants (Table 2). Segmental duplication plays a significant role in the generation of gene families, often results in chromosomal rearrangement and can cause genome instability [55, 56]. The ratio of Synonymous substitution rate (Ks) and nonsynonymous substitution rate (Ka) is less than 1 that indicating the purifying selection of *SlGST* genes pairs during the evolution (Table 2). Identified SlGSTs could be divided into ten classes, where tau and lambda classes were most numerous with 57 and 7 members, respectively (Table 1). However, other eight classes have less than seven members. Usually, tau and phi classes are more prominent in other plants but the lambda class is observed as an exception for tomato GST family. GSTs play important catalytic and regulatory functions for plant growth, development, and tolerance against various biotic and abiotic stresses [57, 58]. It has been reported previously that over-expression of a rice tau GST enhanced tolerance in *Arabidopsis* against salinity and oxidative stresses [59]. Similarly, over-expression of a lambda class rice GST, OsGSTL2, into *Arabidopsis* showed tolerance against heavy metals and various abiotic stresses [60]. These studies revealed the significant contribution of GST proteins in the plant stress modulating pathways.

Catalytic function of GST mainly controlled by the residue in the N-terminal domain [61]. Domain analysis showed that eighty-one SlGST has the highly conserved N-terminal GST domain out of total 90 members (Fig 3). It has been believed that presence of introns in eukaryotic transcripts provide evolutionary conservation by increasing protein diversity through exon shuffling and alternative splicing [54]. Gene structure study showed the presence of ‘0’ intron phase in the gene structure of maximum tau class members which also indicates towards the higher level of conservancy at splicing sites throughout the evolution. Phylogenetic analysis confirmed the high level of similarities among the different classes of plant GSTs in three plant species such as tomato, *Arabidopsis*, and rice (Fig 4). This indicated the ancient evolution of these classes before the split of monocots-dicots.

GST provides physiological flexibility and resistance against various biotic and abiotic stresses [62]. Plant GSTs are reported to involve a number of biotic and abiotic stress responses by conjugating GSH with different targets, including phytohormones, that in turns regulate the homeostasis of phytohormones and GSH within the cells or tissues [63]. Gene expression patterns of *SlGST* could provide important information about their physiological function. Under normal condition, tissue-specific expression patterns of 30 *SlGST* genes represented their imperative role in the growth and development by maintaining high-level constitutive expression (Fig 5). Gene expression analysis showed that a cluster of *SlGSTU*24, *SlGSTF*2, *SlGSTZ*2, *SlDHAR*2, *SlEF1Bγ*1, *SlEF1Bγ*3, and *SlMGST* genes expressed constitutively at all the analyzed tissues and developmental stages of tomato (Fig 5). A cluster of genes *SlGSTU*4, *SlGSTU*5, *SlGSTU*32, *SlGSTU*37 and *SlGSTU*40 showed up-regulation in response to different biotic stresses such as TSWV, *R*. *solanacearum*, *P*. *infestans*, *C*. *michiganensis*, PSTVd (Fig 6A). These genes cluster also showed up-regulation in response to various abiotic stresses- salinity, drought, heat and wounding (Fig 6B). Rest of the members of *SlGST* families responded differentially depending on the mode of stress treatment. However, a prominent dynamic pattern of expression was observed for several *SlGST* transcripts where they showed stress-specific alteration (Fig 7A). Probable reason behind this altered expression might be the presence of various stress-responsive cis-acting regulatory elements in the promoter region of *SlGST* genes (Table 3). Moreover, total tomato GST enzyme activity was also found to be significantly enhanced in response to salinity, dehydration and osmotic stresses. Thus, the up-regulation of *SlGST* transcripts is directly correlated with their corresponding enzyme activity enhancement. SlGST proteins are predicted to interact with other proteins with high confidence limit (Fig 9). Theta and zeta classes of GST have been reported to have glutathione peroxidase activity [64] and thus reduce cytotoxic hydroperoxides resulted from oxidative stress. Glutathione reductase maintained the levels of reduced glutathione by catalyzing the reduction of glutathione disulfide (GSSG) to the sulfhydryl form glutathione [65] and provides a substrate for GST. Glutathione synthase catalyzes the biosynthesis of glutathione (GSH) which is the major component of GST activity [1]. γ-glutamyl transpeptidase (γGT) is involved in the glutathione metabolism by catalyzing the transfer of γ-glutamyl group of GSH to an acceptor molecule [66]. Both GST and γGT are involved in the cellular detoxification process through conjugation reaction. Thus, GSTs are predicted to interact with other cellular proteins of similar function or involved in the same pathways.

Expression of plant defense and detoxification-related genes, such as GSTs and P450s, have reported being induced by safeners [18]. Molecular docking study suggested that flurazole could bind with SlGSTU5 with lowest affinity energy of -6.7 kcal/mol (Fig 8H). Flurazole has been used as a protectant to increase crop tolerance against chloroacetanilide and thiocarbamate herbicides in maize [67]. Model substrate 1-chloro-2,4-dinitrobenzene binds with different safeners such as benoxacor, fenclorim and flurazole to enhance GST activity by 3 to 5 folds [68]. GST activity was found to increase by 8 to 13 folds using p-Nitrobenzyl chloride in presence of safeners [68]. Similarly, 1.5 to 2.5 times enhancement in GST activity was observed in corn using flurazole, dichlormid and cyometrinil safeners [69]. Wheat treated with naphthalic anhydride (safener) showed enhanced activity of TaGST2-3 and detoxified the effect of herbicide (Fluorodine) [70]. Although safeners induced GST activity has been reported in *Arabidopsis*, maize, and wheat; but, there is no such report in tomato till date. Thus, flurazole might be a good candidate to test in tomato to increase GST activity at different stresses and herbicide treatment.

## Conclusions

Taken together, we have accomplished a comprehensive genome-wide analysis of tomato *GST* gene (*SlGSTs*) family and postulated detailed information about them. Our analysis identified a total of 90 GST members in tomato, the largest GST gene family in any organism to date. Comparing to other plant species, *SlGST* family can be assigned into ten phylogenetically conserved classes. The presence of predicted conserved motifs and domains, chromosomal and subcellular localization and their sequence homology with other identified GSTs from other organisms provided insight into their structure and putative functions. Analysis of expression levels in response to three different abiotic stresses, we executed a first step towards the identification of stress responsive *SlGST* transcripts. Results of the present study identified flurazole as potential GST inducer that could be beneficial for crop development and stress modulation. Availability of these informations might encourage researchers for further functional validation.

## Supporting information

S1 Table Primers used in the study. (DOCX)

S2 Table Detailed information of putative conserved motifs in the SlGST proteins. (DOCX)

S1 Text GST protein sequences of tomato, *Arabidopsis* and rice used for phylogenetic analysis. (DOCX)

S1 Data Expression data of *SlGST* gene family at various developmental, anatomical and environmental stress conditions. (XLSX)

S1 Fig MolProbity Ramachandran analysis for the validation of 3D homology model of SlGSTU5. (TIF)

S2 Fig Chemical structures of six herbicide safeners used in the study. (TIF)

S3 Fig Diagram of hydrogen bonding and hydrophobic interactions with six safeners. (TIF)
