# Supplementary material for: Genome-wide identification and expression analysis of glutathione S-transferase gene family in tomato: Gaining an insight to their physiological and stress-specific roles
Source: PLoS One. 2017 Nov 2;12(11):e0187504. doi: 10.1371/journal.pone.0187504 (PMC5667761; doi:10.1371/journal.pone.0187504)
Supplement: S1 Table — (DOCX) [file pone.0187504.s001.docx]

| **S1 Table.** Primers used in the study for semiquantitative RT-PCR | | | |
| --- | --- | --- | --- |
| Sl No. | Gene Name | Primer sequence (5’ to 3’) | Product Size |
| 1 | SlGSTU-5_FOR | ACAAAAAGAGAGCTTGAGCAGC | 169 |
| 2 | SlGSTU-5_REV | TCCTGGCAGGCATTTTGTCT |  |
| 3 | SlGSTU-12_FOR | TCCCGCCATTCTTCCTTCTG | 168 |
| 4 | SlGSTU- 12_REV | TCCAGCAGCAAAGTCCCTTC |  |
| 5 | SlGSTF-1_FOR | AAGCCACCTCCCTAGCCTTA | 169 |
| 6 | SlGSTF-1_REV | TGGGCCGGGTAACATCTCTA |  |
| 7 | SlGSTF-2_FOR | TGGACATTGCAACAATCAATCACA | 155 |
| 8 | SlGSTF-2_REV | AAGCGAATTTTGCTGCCGAG |  |
| 9 | SlGSTT-2_FOR | CGTGAGCGGATAATAGGCCC | 140 |
| 10 | SlGSTT-2_REV | ACAGCATGTCTTTGCTTGTGG |  |
| 11 | SlGSTT-3_FOR | CGATCCATCTCTTATTGCTCGGA | 199 |
| 12 | SlGSTT-3_REV | CCCACTGTGCTTCAACTTTCG |  |
| 13 | SlGSTL-2_FOR | CATCCGCGAGGCCTAAACTA | 170 |
| 14 | SlGSTL-2_REV | ACTAGCCCATGTAACTAGATCAAA |  |
| 15 | SlGSTL3_FOR | AATCGCCAGCCCTGTTTGAT | 199 |
| 16 | SlGSTL3_REV | TTGTGTTCCAGAGAGGGCAC |  |
| 17 | SlGSTZ1_FOR | GCTCTCAATCTTCAGGCAGC | 178 |
| 18 | SlGSTZ1_REV | TTCCAGCAGAACCTGTCAGC |  |
| 19 | SlGSTZ2_FOR | CATGGCCGACTTGTTTCTGG | 163 |
| 20 | SlGSTZ2_REV | ATGATGGATGGCATCAGGCT |  |
| 21 | SlDHAR2_FOR | AAGTACGTCATCGCAGGGTG | 101 |
| 22 | SlDHAR2_REV | CTGCACTCGTCGAACTGCTA |  |
| 23 | SlEF1Bg1_FOR | TGAAGAGGTTTTGCCTCTCCG | 139 |
| 24 | SlEF1Bg1_REV | AGCTGCAATGAGTGCCTTTG |  |
| 25 | SlEF1Bg3_FOR | TGTTGCAGATTTTGCACTCAAC | 178 |
| 26 | SlEF1Bg3_REV | GGTGTTTCAAGCACAGGAACC |  |
| 27 | SlMGST_FOR | GCATACTGTTTTCAGTGTTTGCC | 127 |
| 28 | SlMGST_REV | ACCTAGTTAAGATCAAACGATTCCA |  |
| 29 | *Sl_Ubiquitin_FOR* | CACCAAGCCAAAGAAGATCA | 115 |
| 30 | *Sl_Ubiquitin_REV* | TCAGCATTAGGGCACTCCTT |  |
