# Supplementary material for: Genome-wide identification and expression analysis of glutathione S-transferase gene family in tomato: Gaining an insight to their physiological and stress-specific roles
Source: PLoS One. 2017 Nov 2;12(11):e0187504. doi: 10.1371/journal.pone.0187504 (PMC5667761; doi:10.1371/journal.pone.0187504)
Supplement: S2 Table — (DOCX) [file pone.0187504.s002.docx]

S2 Table: Detailed information of putative conserved motifs in the SlGST proteins.

| No. | Motif | Sites | Width | E-value |
| --- | --- | --- | --- | --- |
| 1 | 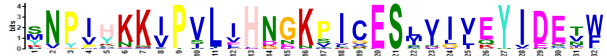  YNHVHKKFPVLVHNDKPICESLVILEYIDETW | 58 | 32 | 3.1e-1265 |
| 3 | 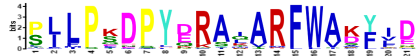  HLLPQDPYEKAMARFWAKFVD | 54 | 21 | 1.4e-698 |
| 3 | 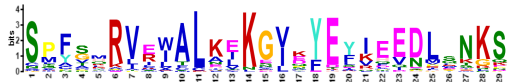  SPFALKVHWALKLKGIEYEYQEEDLS | 63 | 29 | 1.2e-787 |
| 4 | 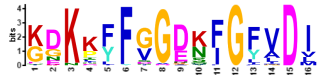  KEKKFFGGESIGYVDL | 44 | 16 | 5.4e-351 |
| 5 | 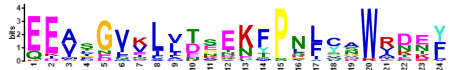  QEAIGMELLTEQKFPKLSKWIDEF | 35 | 24 | 2.2e-269 |
| 6 | 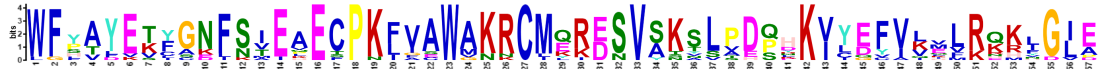  WFHAYEVFGDFKVEQECPKFGCWMKRCLERESVSSVLPDPEKIYQCVVMLRKMHGIE | 14 | 57 | 1.2e-367 |
| 7 | 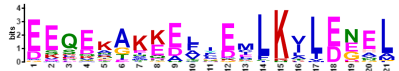  EEREKGCDETFEVLKYLDNEL | 44 | 21 | 3.6e-266 |
| 8 | 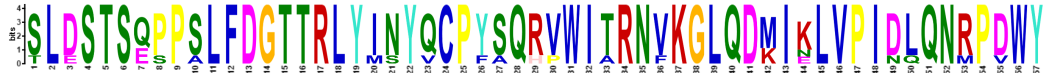  SLDSTSQPPSLFDGTTRLYINYQCPYSQRVWITRNVKGLQDMIKLVPIDLQNRPDWY | 6 | 57 | 9.9e-216 |
| 9 | 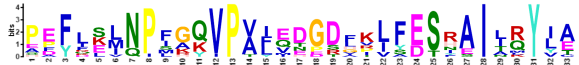  EPFISLNPFGQVPAFEDGDLKLFESRAITQYIA | 15 | 33 | 1.6e-159 |
| 10 | 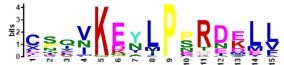  APAIKQHLPPRDKLV | 30 | 15 | 1.1e-114 |
