# Supplementary material for: Genome-wide identification and expression analysis of glutathione S-transferase gene family in tomato: Gaining an insight to their physiological and stress-specific roles
Source: PLoS One. 2017 Nov 2;12(11):e0187504. doi: 10.1371/journal.pone.0187504 (PMC5667761; doi:10.1371/journal.pone.0187504)
Supplement: S1 Text — (DOCX) [file pone.0187504.s003.docx]

**S1 text. GST protein sequences of Tomato, *Arabidopsis* and Rice used for phylogenetic analysis**

>SlGSTU1

MQNLEFIEEQLKGKKFFGGESIGYVDLALGWMAYLLDVFEEVLDLKLFDADKFPLLSGWTKNFCDAPAIKQHLPPRDKLVTKFQLFHEKFQTAN*

>SlGSTU2

MADEVKLYRTWSSRFSLRIIWALHIKGIEYEAIFEDLSHKSPQLLKYNHVHKKFPVLVHNDKPICESLVILEYIDETWKETSHLLPQDPYEKAMARFWAKFVDDKFKNLNVLNHIIYGPKQNFIIN*

>SlGSTU3

MADEVKLYRTWSSPFGLRIVWALHIKGIEYEAIFEDLSQKSPQLLQYNPVHKKIPVLVHKGKPICESLVILEYIDETWKETAPLLPQDPYEKAMARFWAKFVEDKLLPSVWSIFTEQGYDAKKEAFVPAVQNLEIIEEQLKEKKFFGGESIGYVDLVLGWMAYLLDVFEEVLDLKLFDADKFPLLSGWMKNFCDAPAIKQHLPPRDKLVTKFQLLHEKFQTAN*

>SlGSTU4

MASEKVKLLGYWASPFALKVHWALKLKGIEYEYQEEDLSNKSPLLLQYNPVHKKIPVLVHNGKPIAESLVILEYIEETWKHNPLLPEDPYERAKARFWAKFVDDKCVPGIFGTFAKVGVEQQKIAKEARENLKILEDELGKKHFFGDAKIGFMDVTSAWIICWAQIVEEVVDIRLIDAEEMPSLVSWFQNVLEAAPILKECTPPKDKLLEHNKGFHEMLVASAPS*

>SlGSTU5

MGEVKVHGIFAGPFNKRVELALKLKGVKYEYIEEDRSNKSDELVKYNPIYKQVPVLVHNGKPICESIIILEYIDDTWENNTIPLLPKHPYQRSMARFLAKLIDEKLMGAMYKVCYGKGEEREKGCDETFEVLKYLDNELQNKKFFGGDSIGFVDIVASYIALWFGAIQEAIGMELLTEQKFPKLSKWIDEFLCCRIVKENLPNREVLVPLYKAQFAAATQKASS*

>SlGSTU6

MEDVKLLGTKESIFTQRIIWALKLKGICYEFIEQDFSSRSSPLLVKLNPVYNKVPVIVHDGKSLAESLVILEYIEETWPLINPLFPLDPFQRASTRFWARFVDGKFYEAAKKAFFSSGETKAEGVESVVEGLHLLEGQIIGKKFFGGEKIGYLDIITGWIAYWFQYIEEIGEFKAMDSTKYPCLHAWINNFIQLPIIKQSLPTPDVVKSVFRGFKDAAALADAN*

>SlGSTU7

MSKDDELRLLDFWASPFCMRVKIALSEKGVAYESQQEDLFGGKSDMLLKSNPIYEKVPVLLDNGKPIVESNNIVYYIEDKYPSTNPLLPSCAYGRSRARFWADFIDKKIFEGGMCIWKSKGEELEIAKKDFIEILKKLEGAMGDKDFFGGDNFGYVDVIAIAMTSWFHAYEVFGDFKVEQECPKFGCWMK RCLERESVSSVLPDPEKIYQCVVMLRKMHGIE*

>SlGSTU8

MGEENKVTLHGMWLSPYVKRVELALKVKGIPFEYIEEDLSNKSPLILKYNPIHKKVPILVHNGKPVNESFVIVEYIDETWKNGPQLLPEDPYERSKVHFWAAYIQQVMESMLNIFTAEDQKQACNEFHQKFRLLEDGMKNFFPTIENRNIGLIDIWIVVAFGMCKAQEEAFGVNFLDPEKVPLIHSRVNS LLELPLLRET VPDHDKAVSFLRALKETSTK GQAH*

>SlGSTU9

MEEENDRVTLHGMWISTYAKKVELALKIKGIAFDYVEEDLSNKSSLLLKYNPIHKKVPLLLHRGKPLSESLVILEYIDETWNNLQPLLLPEDPYERATVRLWASYCLQISDTMKKAFISARDVEGGAFDELFEILKVMEEGMKDFFPGGRSKICAENLGLLDIIIVCSLATYKAAEEVVGTKILDPEKNPFVYSWVTTLLELPLVKETLPPHDKLVSRLDFIKKNGFRFQ SNI*

>SlGSTU10

MAKNNLKILGAWPSPYVMRPRIALNIKCLAYDFLEEQFGTKSELLLKSNPIYKKIPVLIHDGKPICESLIIVQYIDENWTNFGHSILPSHPYDRAIARFWAFYIDDKWFPALCGVAAAQDEDAKKAAMETVIEGLVLLEDVFKINSKGKKFFGGDKIGYLDIALGCFLGWLKVNEKLNNVNLLDESRTPSLYQWAKDFCVDSVVKDVMPETDQLVQAAKIIWAKI*

>SlGSTU11

MRPRIALNVKSVCYDFLEEQLSSKSDLLLISNPVYKKIPVLIHDGKSICESLNIVQYIDEKWTNSGPSILPLDPYDRAIARFWACYIDDKWFPLFRSFAVAQGEDAIKTALEPVFDGLVLLEDAFKNCSKGKKFFGGDKIGYVDIALGCFLGWMRVIEKMNNVTLLDEAKTPGLYNWAEDFCADSSVKDVMPETNKLAEAAKNLIPKIRANASS*

>SlGSTU12

MATNSVKLLGTWACPYVYRVEIALKMKSIEYEFIQERVFNKSELLLKSNPVYKKIPVLFHDEKPICESLVILQYIDEAWLNGPAILPSDPYDRAIARFWAAYIDEKWYPLVADYRNVEGKEAKAAMVEKISEGTLLLEEAFINMSKGKSYFGGDSIGYVDIVFGSLLGWVKVIEIVDELKILDETKTPSLAEWDEKFCSHNVVKDIIPETEKLVEIYHKYVELKKANLS*

>SlGSTU13

MATSIIKVLGTPASPFANRVSIALNVKSVDYEFVQEDMSNKSELLLKSNPVYKKIPVLILGENIICESLVIVQYIDETWTNGPSVLPSNPLDRAITRFWVAYIDCKWLPLMSDLGKAQGEEAILEVQEKLQQALVPLEEAFVKCSKGKSFFGGENIGYIDIALWCILGWIKAIKIMLGIEIFNVTKAPELVNWGNRFLEDKCVKGAMLEPEKLVEIVKLHLAKKEANNAN*

>SlGSTU14

MNIFKVNGWTCTPPLQLSLKIKSIEYEFIQEHILNKSELLLKSNPVNKKIPILFHDEKPICESLVILQNIDEPWLNGPSILSSDPYNRSIARFWAAYIDDKEYRNAEGKEAKAVVVDKMSEGNMLLEETFIKISKGKSFFSGDSIGYVDIVLGSLLGWVR*

>SlGSTU15

MGQVKLIGSSGSLFCTRVEWALKLKGVDYEYIQEDLLNKSELLIKSNPVHKKIPVLLHDDKPVVESLLILEYIDETWKGYPLLPQDPHERATARFWAKFVDDKCVIGSWEAMAMQDEGEAKTKAIESIQELYAFIEKQIEGKKFFGGEQIGYLDLVMGWKTLWLSAMEEVGNVKLLDPEKFPSLHQWAENFKQIPIINECMPQQETLVNYFQVGLNYLRSLAANKP*

>SlGSTU16

MKEENNVTLHGMWAKSTNTVHNGNQICESSVIIEYIDETWKNESPLFPQDPYQRIKVRFWASYIHQVYDCMLKVFRGKEALEGFYAKLSVLEDGINNFSLGITSNMNNIGMLDIMIVITLGAYRVQEEVFGFKLLEEENTSLLYSWVTTLIELPIVKGITPPHEKVVSFLQYLKNKVFKAPPHAS*

>SlGSTUU17

MTGRRVWSGKGEDQEEAKELIEIFKTLEGELGDKTYFGGDEKLGFVDVTCANFSIEAECPKLVAWAKRCMEIENVSNSLTHPHKIYGYVLELKHKVGLA*

>SlGSTU18

MANDEVILLDFWPSMYGMRLRIALAEKEIKYEYRDEDLRNKSPLLLQMNPIHKKIPVLIHNGKPICESIIGVEYIDEVWKDKAPLLPSDPYERAQARFWADYIDKKLYATGSKIYTATGDEQEAGKKDFVEILKVLEGALGEKPYYGGDNFGFGDIALIGFYCWFHAYEVYGNFSIEAECPNLVAWAKRCMQRDSVAKTLPDQHKIIEFVKILREKLGLE*

>SlGSTU19

MNPIHKKIPVLIHNGKSICESIIGVEYIDEVWKDKAPLLPSDPYERAQARFWADYIDKKLYRSARKIWGTKGEEQEAGKKDFIEVKVLEGALGEKPYFGGDNFGFVDIALIGFYSWFHSYETYGNFSTEAECPKFVYHQF*

>SlGSTU20

MANDELILLDFWASMFGMRLRIALAEKGIKYEYKEEEGLISNKSALLLEMNPIHKKVPVLIHNGKPICESIIGVEYIEEVWKDKAPLLPSDPYERAQARFWVDYIDKKLYVSARKIWGTKGEEQEAGKKDFIEVLKVLEGELGEKPYFGGDNFGFVDIALIGFYSWFYAYETYGNFSAEAECPKFVAWAKRCMQRDSVAKSLPDQHKVLEFIQMLRRKFGIE

>SlGSTU21

MANDEVILLDFWPSMFGMRLRIALAEKEVKYEYKEEDVWNKSPLLLEMNPIYKKVPVLIHNGKPICESIIGVEYIEEVWKDKAPLLPFDPYERAQARFWADYINKKCETYGNFSLEAECPKLVAWAKRCMQRDCGQVFA*

>SlGSTU22

MANDEVIVLGFWPSMFGLRLRIALAEKEVKFEYREEDLKNKSPLLLQMNPIHRKIPVLIHNGKPICESIIGVEYIEEVWKDKAPLLPSDPYERTQARFWADYIDKKFYWPARKLWTTKGEEQEIAKKDFIECLKVLEGVLGDKPYFGGDNFGFVDIALIGFYCWFSAYETYGNFSTEAEFPKFFAWAKRCMQRDSVAKSSPDQHKVLEFVKVVRQRLGIE

>SlGSTU23

MANNEVILLDFWPSMFGLRLRIALAEKEVKYEYREEDLPNKSPLLLQMNPIHKKIPVLIHNGKPICESIIGVEYIDEVWKDKAPLLPSDPYERAQARFWADYIDKKFYWASRKLWTTKGEELDAAKEEFIVCLKVLEGALGDKPYFGGDNFGFVDIALIGFYCWFSAYETYGNFSTEAESPKFVAWAKRCMQRDSVAKSSPDQHKVLEFVKVVRQRLGIE

>SlGSTU24

MANDEVILLDFWPSMFGMRLRIALAEKEIKYEYRDEDLRNKSPLLLQMNPIHKKIPVLIHNGKPICESIIGVEYIDEVWKDKAPFLPSDPYERAQARFWADYIDKKLYDSGRKLWTTKGEEQETAKKDFIECLKVLEGALGEKPYFGGDNFGCVDIALIGYYSWFYAYESYANISVEAECPKFVAWAKNCMLRDSVAKSLPDQHKVCEFVKVLRQKFGIE

>SlGSTU25

MADEVVLLGTYVSMFAVRVKIALAEKGIQYEYKEENLVNKSPLLLQMNPIHKKIPVLIHNGKPICESLIIVEYIDEVWNDKSPLLPSDPYKRAQARFWADYVDKKIYDGGKKIWTTKVEEQEAANKEFIECLKVLEGELGDKPYFDGESFGFVDLALIPYYSWFPAYEKFGKFSIEPECPKFVAWANRCMQKENVSKYLSDPDKIYDFVVMLRQRIGIA

>SlGSTU26

MGDEVVLLDTFVSVFGMRVRIALAEKGIQYEYKEEDLMNKSQLLLQMNPIHKKIPVLIHNGKPICESLIIVEYIDEVWKDKSTPLMPSDPYKRAHARFWADYIGKKIYDGGMKIWSSKVEEHKTANKDFIECLKVLEGELGDKPYFDGKNFGLVDMAFIPYYSWFPVYKKLSNLNIEAECPKFVAWAKRCMQKESVSKTLVDPDKIYEFIVLFRQKIGVA

>SlGSTU27

MGDEVVLLDLWVSPFGMRVRIALKEKGINYESKEENLSNKSSLLLKMNPIHKQIPVLIHNGKPICESLIIVQYIDEVWKDKAPLLPSDPYERAHAKFWADYVDKKIYSTGRLVWTTKGEAQEAAKKELIHHFKLLEKELGDKTFFGGDQFGLVDIALIPFYSWFYALETCGNFSMIHECPKLVEWAKRCMERESVSTSLPDQYKVYDFILELKKKLDLD

>SlGSTU28

MEEENKVTLHGMWTSPYVKRVERALKVKGIHYEYVEEDLMNKSELLLTYNPIHKKVPILVHNGNPICESSVIIEYIDETWKNESPLFPQDPYQRAKVRFWASYIHQVLLLYSTFLLSSQIHIKQTNPYTIFLIDLYIS

>SlGSTU29

MVDVKLLGLWYSPASHKVEWALKLKGVKYEFIEENLQNKSPLLLESNPVHKKIPILIHNGKPICESMIILEYIDETFEGPSILPKDPYDRALARFWAKFLDDKVGAMVNTFLLKGEEQEKGKKEVCEMLNVLDNELKGKKFFVGDKFGYADMAANFVGYWLGVFQEASGVVLVTSEKFSNFCVWRDEYVNCSQVKEYLPPRNDLLAFVEARTQASASKA

>SlGSTU30

MADVKLLGLWYSFFSHRVEWALKIKGVKYEIIEEDLQNKSPLLLQSNPIHKKIPVLIHNGKSICESMIILEYIDETFEGPYILPKDPYDRALARFWAKFLDDKVGAVVSTFIRKGEEREKGKEEACEMLKVLDNELKDKKFFVGDKFGFADIAANLVGYWLGIFQEASGVELVTSEKYPNFCAWRDEYMNCSQVKEYLPPRNDELLAFFQGCAAAASASTQN

>SlGSTU31

MVILEYIDETFEGPSILPKDPYDRALARFWAKFLDDKVVTVVNAFLGKGEENEKAKEEVYEMLKILDNELKNKKFFVGDKFGIADIVANLVGLWLGVFQEGSGVELVTSEKFPNFCSWRDEYVNCSQVKEYLPRRDDLLAFFQAFTRAQAAASASTQK

>SlGSTU32

MAQVKLLGFWYSPFTHRVEWALKIKGVKYEYIEEDRYNKSPLLLESNPIYKKVPVLIHNGKPICDSIVILEYIDEIFEGPSILPKDPHERALARFWAKFLDDKVGAVVNTFLRKGEEQEKGKKEVCEMLKVLDNELKDKKLFVGDKLGFADMVANLVGLWMSVFEEASEVVLATNENFPNFCAWRNTYISCNQVKEYLPLRIDELLAFYQDRVRALATTLATPQK

>SlGSTU33

MLKVLDNDFKDKKLFVGDKFGFVDIVANLVELWMGVFQEATGVVLATNENFPNFCARRDTYMNCSQVKEYLPSRIDELLV

FYQAYIRHSSYNFCFS*

>SlGSTU34

MVDEVKLLGVSGSSYSRRVEWALRVKGVKYEFIEEDLQNKSPLLLESNPVLKKIPVLIHNGKSICESMVIVEYIDETFEGPSILPKDPYDRAIARFWATFLDGMCLDAVRKGLWSKREEKEKNIQEEAYEMLKIVDNELKDKKFFSGDKIGFVDVAANYIPFWVEIVEEATGNVLITSEKFPNLCAWIDKYLKCSEVQENLPDRDMMLSFFKAKALAEIGAK

>SlGSTU35

MADVKLLGLWYSPYSHRVEWALKIKGVEYEFIEEDLRNKSPLLLESNPIYKKIPVLIHNGKPICESMVIVEYIDETFEGPSILPKDPYDRAIARFWAKFFDEKGSSVGRSFFLKGEEQEKAKEELHEMLKVVDNELKDKKYFVVDKFGFVDIVANVVALWLGVLEEASGVVLVTNEKYPNFYAWRDEYINCSENKKYLPSRNELLAKFKARILAASVAE

>SlGSTU36

MEEVKLLGLWYSPFCHRVEWALKVKGVKFEFIEENLQNKSPLLLESNPIHKKIPVLIHNGKSICESMVIVEYIDETFEGPSILPKDPYDRVIARFWVKFFEDKGSAVGTSFFHKSEKAKEEVCEMLKILDNELKDKKFFVGDKFGFADIAANFLALWMGILEEATGIILVTKEKYPNFYAWRDEYINGNKEYLPSRDELLAFFKARFQAAATPPYSN

>SlGSTU37

MGDVKLLGLWYSPFSHRVEWALKFKGVQYEFIEQDLQNKSPILLESNPIYKKVPVLIHNGKPICESIVILEYIDEVFEGPSILPKDPYNRALARFWVKFFEDKGPSMRKSILLKGEEQEKAKEEVFEMLRILDNELKGKKFFVGDKFGFVDIVANAGALWLGVLEEVSGVVLVTKEKFPNFCVWRDEYCTQNKEYLPSRDELLIRFKTYI

>SlGSTU38

MADIKLLGLWYSPFSKRVEWALKTKGVEYEYIEDDLQNKSLLLLQSNPIHKKVPVLIHNGKPICESSVILEYIDETFEGPSILPKDPYDRALARFWAKFFEDKWPSMMKSLFFKGEEQEKGKEEVNEMLKILDNELKDKKFFVGNNFGFVDVVANAVALWFGVLEEVIGVVSVTSEKFPNFCDWRDEYYIQNKEYLPSRDELFAHYQAYIQRVAASK

>SlGSTU39

MAEVKLLGLSYSPFNHRVEWALKIKGVKYEFIEEDLQNKSSLLLESNPIHKKIPVLIHNGKPICESMVILEYIDEAFEGPSILPKDPYDRALARFWAKYVDDKGSAVWKSFFFKGEEQEKAKEEAYEMLKILDNEFKDKKYFVGDKFGFADIVANGAALYLGILEEVSGVVLATSEKFPNFCAWRDEYCIQNKEYFPSRDELLIRYRAYIQPVDASK

>SlGSTU40

MADVKLIGLWYSPFSRRVEWALKIKGVEYEYIEDDLHNKSLLLLQSNPIHKAVPVLIHNGKPLCESSVILEYIDETFEGPSILPKEPYDRSLARFWAKFFDDKGLAIRKSIFFKGEEQEKAKEEVYDMLKVLDNELKNKKIFVGEKFGFVDIVANAAALWLGVLEEASGVVLVTREKYPNFCDWRDEYCTQNKKYLPPRDELLAHYQVYIQRVTTSK

>SlGSTU41

MVIVEYIDKTFEGPSIIPKDPYDCAIARFWAKFLDDKMPPVGKSFFLKGEEQERAKEEAYEILKILDNELKGQEVLCW*

>SlGSTU42

MGDVKLLGLWYSPFSHRVEWALKIKGVQYEFIEQDLQNKSPLLLESNPIHKKIPVLIHNGKSICESMVIVEYIDETFEGPSILPEDPYDRALARFWVKFLEDQIAAVGKSIFLKGEEQEREKKAACEMLKILENELKDKKFFVGDKFGLADIAANVLAIWLGVFEEASGVALVTSENYPNLYGWRNEYCNQNKEYLPSRDELLIHFQPRFPAKAK

>SlGSTU43

MAGVKLLGISLSPFSRRVEWALKIKGVEYEFVEEDLHNKSPVLLELNPIHKKIPVLIHNGKPICESMVIVEYIDETFEGPSILPKDPYDRAIARFWAKFFDDKCMPVMGKAIFGSGEESNKAKEELGDLIKILENELKDKNFFVGDKFGFADMAGNLMAYWMGIVEEASGNIFVTSEKFPIFCNWRNEYVNCSTIKEYLPPRDEILAHFKARFAAAQK

>SlGSTU44

MGKIKLLGVSLSPFTHRVEWALKIKGVEYELIVEDPQNKSPLLLEYNPIHKKIPVLIHNGKPICESMVIVEYIDETFEGPSILPKDPYDRATARFWAKFLDDKCLPTMGKALLGNEEEKEKAKEECGELLKILDNELKDKEFFVGDKIGFVDIAANALAFWMGIIEEASGVILVKNEKFPNYYTWRDNYINCSQVKKYLPSRDELFSHFQSRFHSASTTK

>SlGSTU45

MLGMKQEVYTQGIEYEFIEAQRPIKKCPNIIKYNPIYKKVPVFLHKGNPIPESLVILEYIDENWKDGTSLLPKDPYQRAIARFWAKFIDEKCLPEILKLCYDSNYEVKVKAMGELQELLKLLENELMKDNNKIFFGGENKVGYMEIVSILITYWLGVMQEALGVDILNKKEFPNICGWADKVISFSFMKENLPPREKLLAIYKEYAQPLVPPNNEINHTHK

>SlGSTU46

MEEQVKLFGAFPSPFSHRIIWALKHKNISYEYIEEDLSNKSQHLLTYNPIYKMIPILVHNEKPIVESTIILEYIEETWPQNPLFPKDPYEKAKARFWIKFGEDKNSEFHQIFHKIGEEQVKATENAKKILKIIEEQGLGDKKFFSGDTIGLIDIAFGWLAFWLEVIQEAAGVKVYEPNNFPHLQSWINNFKQVAIIKENIPNRNAMLDYFKLRRDMIVAL

>SlGSTU47

MDQDLKLHGSWASPYSLRIIWALKLKGLLYEYIEEDLANKSDLLLKYNPIFKKIPILVHDGKPICESMIILEYLDQIWPNQYPLLPIDSYQRALARFWVNYFEQKSVSLWMIFRSKGEEQEKAVKDSLEMLKIIEENAFKNQKNNIFFIGGKIGIVDISFGWICHWLKIIEDVGGVKLIEENSFPNLQNWMKKFKEVPLIKESLPNHQKLFLPFKLIRDMLLAS

>SlGSTU48

MDEVKLHGTSYNLFTYRVIWALKLKGIPFEYIEEEHSNNGSLIMKYNPVFKRFPILFHGEKVISESMVIIEYIEDTWPQNPLLPIDPLDRSIARFWVKFAGDKGACVGTMYYTSGEKQEKAIKETMEMLKIIEEQAFIEDEENIFFGGEKIGIVDLAFGVIPHWLEIIEDIIGVKLLEPNLFPNLLNWVQNFKEEQIIKENLPNYEEMFVFLKNPKKMKLSSS

>SlGSTU49

MANEEVILLDFWPSMYGMRVRVALAEKCVNFEYKEQNMIEKSPILLEMNPIYKKIPVLIHNGKPICESLNVVQYIDEVWKNKVIFLPSDPYEKYQAMFWADYVEKVFDTGRKLWMEKGGEKQTRKGNYIDTLRMLEGIIGDKLYFGGEKFGYLDICLIGICSWFYTYEKFGEFSTEVETPKIIAWMKRCMKRESVYKNVVEPLKVYDFALQLRKHYGIE

>SlGSTU50

MENDEVILLDFWPSMFGMRVRVALAEKAIEYEYKEEDLFTSKSPLLVKMNPIHKKIPVLIHNGKPVCESFVVVEYIDEVWKDKAPLLPSHPYDRSQARFWASYTDKLYDFGRRIWTVKREEFAEGKKDFIDPLKLLEEAALGDKPYFGGESFGFVDIALIGFYSWFYTYETICNFSIEAECPKIAAWGKRCMKRESVSKSLADSRKIYEVVIEFRKKNGLE

>SlGSTU51

MSRVKLLGVYGSPASQRVEWALKIKGVKYEFITEDLQNKSPLLLKSNPVYKKIPVLLHNDKPIAESLVIIEYIDEAFEGPSILPKDPYDRAIARFWVKFLDEKCLPAVWKALWSQGDEQEKDKEEAYEVLKVIDNELKDKKFFGGDNIGFVDVVANFVGFWIGIVEEATGVVLVTSENFPNFCAWRDEYLNCDRVKENMPSREMLLGYFKSRVQAVAAISK

>SlGSTU52

MEEEVILLDFWCSMYGMRARIALEEKGVKYEYKEEDLKNKSPLLLQMNPIHKKIPVLIHNGKSICESLVIIQYIDDVWKDIGPLLIPKDPYDKAQAWFWSDYMDNTVHEYARKTWATKGEEQEQAIKDFLGGLKLLEGVLGDKPYFGGENFGFLDVSLIGYYSWFLAYETFGKFNVELECPKLISWVKRCMERESVSKALPDSKKVCEFVLHLRNKIGLE

>SlGSTU53

MSNEVVLLSAYVSMFGMRVRIALHEKGIQYEYKEEDLSNKSELLLQMNPIHKKIPVLIHNGKPICESLIIVEYIDEVWKDKSPLMPFNPYKRAQARFWADFIDKKVYDSGKRIWATKGEDQEAAKKEFIEYLKLLEGELGDKTYFNGENFGFVDLALIPFYSWFPTFEKFGNFNIEKECPKFVAWANKCIYKDSVSKSLAESNKVYEYVLKMKQHLGLP

>SlGSTU54

MTQEEEVVLLDYWASPFGTMARIALVEKGVNFIHKFEDLSNKSPLLLEMNPVHHKIPVLVHKGKSICESNIIIQYIDEIWKNNSPLLPYEPYQRAKARFLVDFINKKVHGSSVKVWMGQIEEQENGKKELVECSKFLEEELGDKLYFGGDVFGFVDIALVPFYNWFIVFKTFANFNTIEIQCPKLVMWGERCLNRDSVSKSLPTSNQVYQAYLDFKKGW

>SlGSTU55

MAELTLLGLCYNPFSHRVEWALKIKGVKYEFIEEDLRNKSLLLLKSNPIYKKIPVLIHNGKCICESMVILEYIDEAFEGPSILPKDPYDQALARFWAKYVDNKVYFFSFL

>SlGSTU56

MEEENKVTLHGMWANPYVKRVELALKVKGIPYEYVEEYLMNKSELLLTYNPIHKNTWKNESPLFPQDQYQRAKVRFWASYIHQVYDCMLKVFRGKEALKRFYAKLSVLEDGINNFSLGITSNMNNIGMLDIMIVITLGAYKVQEEVFGFKLLEEENTPLLYSWVTTLIDLPIVKGITPPHDKVVSFLQYLKNKVFKAPPHAS

>SlGSTU57

MEIVKLIGTPFSFFTYRVIWALKLKGINYEYIDEDMSKKSSLLVKYNPIHKKVPVLIHGDKIICESMVIVEYINETWKLNPLLSTDSYERATSRFWAKYIEEKSHSSWNVFCYTGEKQQNAIKESLEMFKTIEENALGENNILFGGENIGFVDIAFGGYSLWMEIIEEIVGIKLLNPHNFPRINNWIKKFKEVQTIKDNLPNRDEMFVYMKNARGRMLASP

>SlGSTF1

MVVKVYGSAMAACPQRVMVCLIELGVDYELIHVDLDSLQQKKPDFLLLQPFGQVPVIEEGDFRLFESRAIIRYYAAKYEDKGKKLTGTTLEEKALVDQWLEVESNNYNDLVYNMVLQLLVFPKMGHKSDLIVVQKCANNLEKVFDIYEQRLSKSKYLAGDFFSLADLSHLPSLRFLMNEGGFAHLVTQRKYLHDWYLDISSRPSWSKVLDFMNLKKLEMLPGPPKEEVKV*

>SlGSTF2

MAIKVHGPMMSPAVMRVVATLKEKDLDFELVPVNMQAGDHKKEPFISLNPFGQVPAFEDGDLKLFESRAITQYIAHTYADKGNQLLPNDPKKMAVMSVWMEVEAQKFDPIGSKLGFEIVIKPMLGMVTDDAVVAENEEKLGKLLDVYESRLKESKYLGGESFTLADLHHAPSLHYLSGSKVKSLFDARPHVSAWVADILARPAWSKTIELSKQ*

>SlGSTF3

MAIKVHGPMLSPAVVRVVAMLKEKNLDFELVHVDLQNGDQKKEPFISLNPFGQVPAFEDGDLKLFESRAITQYIAHTYADKGNQLLPNDPKKMAIMYVWIEVEAQRFEPVVSKLCYEIVIKPLLDMVTDDAIVAENEEKLSKLLDVYESRLKDSKYLGGDSFTLADLNHAPALHYLMGTKVKSLFNARPHVGAWVANILARPAWAKSLELTK*

>SlGSTF4

MRVISCLIEKDLDFEFVFVDMAKEEHKRHPFLSLNPFAQVPAFEDGDLKLFESRAITQYIAQVYASNGIQLILQDPMKMAIMSVWMEVEGQKFEPPASKLTWELVIKPMIGLGSTDDVIVKESEEQLSKVLDIYETRLTESKYLGGDSFTLVDLHHIPNIYHLMNTKAKALFDSRPRVSVWCADILARPAWVKGLEKMQK*

>SlGSTF5

MATPVKVYGPTLSTAVSRVLACLLEKNVQFHLIPVNMAKGEHKKPAYLKIQPFGQVPAYQDEDITLFESRSINRYICDKYGSQGNKGLYGTNPLEKASIDQWIEAEGQSFNPPSSVLVFQLAFAPRMKLKQDENLIRQNEEKLKKVLDVYEKRLGDSQYLAGDEFTLADLSHLPNIQYLVNGTDRAELITSRENVGRWWGEISNRESWKKVVEMQTSPPPS*

>SlGSTF6

MQLYHHPFSLNSQKVRLTLEEKGIDYTSHHVNPLTGKNMDAFFFSMNPSAKVPVFQNGSHIIYDTIEIIQYIERIAEKVSSGGNNLNLSSREVIGWMHKIQEWDSMYFTLFHVPEKYRLCVSKFLRRVIIARMAESPDLASAYHCKLRQAYDTDDKLKNADVLRRSENHLVRLLDEVELKLGETSYLAGEEFSLADVMLIPLLARIELLNLENEYINSRPNIADYWVLVKQRPSYKKVIGKYFDGWRRRKTLLKTWCFIRVRSVLRKY*

>SlGSTT1

MVVKVYGSAMAACPQRVMVCLIELGVDYELIHVDLDSLQQKKPDFLLLQPFGQVPVIEEGDFRLFESRAIIRYYAAKYEDKGKKLTGTTLEEKALVDQWLEVESNNYNDLVYNMVLQLLVFPKMGHKSDLIVVQKCANNLEKVFDIYEQRLSKSKYLAGDFFSLADLSHLPSLRFLMNEGGFAHLVTQRKYLHDWYLDISSRPSWSKVLDFMNLKKLEMLPGPPKEEVKV*

>SlGSTT2

MTLKLYVDRMSQPSRAVIIFCKLNGIDFEEIHINLSKRQQLSPEFKEINPMKQVPAIIDGRFKLFESHAILRYLACAFPGIADHWYPADLYKRAKVDSVLDWHHSNLRRGAAGYIFNTVLAPAFGLPLNPQAAAEAEKVLLASLAKVESVWLQRKGRFLLGSGQPSIADLSLVCEIMELEILDEKDRERIIGPYKRVLKWIDDTKNAMEPHFQEVHVILFKAKEKFHKQRHAVGSSIPQSSRKPDLHSKM*

>SlGSTT3

MTLKLYVDRMSQACREVIIFCKLNGIDFEEVHIDLSKRQQLSPEYREINPIRQIPAIMDGRFKLSESHAILKYLACAFPRIADHWYPADLYKRAKVESVLDWHRTNFPRGPGSYTFYSVLAPTVGLPLNTKAAARTEKMFIACLATIESVWLQKKGRFLLGSDQPSIADLSLACEIMQLEILDEKDRERILGPFKRVLKWLDDTKNAMAPHFEEVQSTLAGYKEKVQKQRNTLGSKITQSGRKPVLQSNM*

>SlGSTT4

MSLKVYVDRLSQPSRAILIFCKLNGIEFEEVNIDLAKGQHRTPEYQEVNIMKQVPAIVHDTFKLFESHAILRYLASAFPETADHWYPKDLQKRANVECVLDWHHANLRRGSAGYVFNTILAPAFGLPLNPQAAAEGKNLLSASLATIDTYWLQKDGSFLLGNSQPSLADLSLVCEIMQLQFLDEKDREGLLSPHKNVLKWIDDVKSATAPYFDEIHATLFKVSEIFQKQRAGGASS*

>SlGSTL1

MAASSIGHQIHINVNSPILLPLRTNFSSLSFTFSNARYPLKWNHIGCPKICALPAVSIIASGSSREMLPPALDSSSEPPAIFDGTPKLYISYSCPYAQRTWIARNCKALQEEIKLVPIDLKNRPDWYKEKVYPANKVPSLEHNNEVKGESMDLIRYIDSNFEGPSLFPDDPSKREFAEELFSYFDSFYKAVISSLKEDKINDAIAAFDSIETALSKFVDGSFFLGSLSLVDIAYAPFIERFQPFLLEVKNYDITTGRTKLAAWIKEMNQIEGYTVTKRDPKEHLENYKRRFLSQL*

>SlGSTL2

MAALSVQEVLPATLESTSEPPSLFDGTTRLYINYQCPYSQRVWITRNVKGLQDKINLVPIDLQNMPDWYKEKVYPQNKVPSLEHNNKMIGESLDLVKYVDSNFEGPSLLPDDPEKRKFAEELIAYSDIFVPEVYKSFFRDAQTLAGAQFDYLEKALDKFDDGPFFLGQFSQVDIAYVPFIERFQIFMEKGINYDITSARPKLAKLIEEMNKLDGYKQTKVLDPEKLVEYYKNLFLKKA*

>SlGSTL3

MATPSVQEIRPASLDSTSESPALFDGTTRLYISYVCPFAQRPWIARNFKGLQDKIELVPIDLQNRPVWYKEKVYPQNKVPSLEHNNKVIGESLDLVKYIDSNFEGPFLLPDDPEKQKFAEELIAYSDTFLKEIYANFKGDIEKHAGPQFDYLEKALDKFDDGPFFLGQFSQVDIVYAPFVERFQIFLKEGLNYDITSGRPKLAKWTEELNKLDSYIQTKADPKEVVDLYKKKYLA*

>SlGSTL4

MASPSVQDLLPPSLDSTSQPPSLFDGTTRLYINYQCPYSQRVWITRNVKGLQDMIKLVPIDLQNRPDWYKENVYPKNKVPSLEHNNKVTGESLVLVKYVDCNFEGPSFLPDDQEKRKFAEELIAYSDTTFVPEVYRSFAKDARTLAGAQFDYLEKALHKFDDGPFFLGQFSQVDIIYAPFVERFHVFMPEGFNYDITTGRPKLAKWTEEMNNLDGYKQTKVLEQEKMIEYYKNRFLPKA*

>SlGSTL5

MLLTQPVLLLSPLPFKKKQLSMASPSVQDLLPPSLDSTSQPPSLFDGTTRLYINYQCPYSQRVWITRNVKGLQDMIKLVPIDLQNRPDWYKEKVYPKNKVPSLEHNNKVTGESLVLVKYVDCNFEGPSFLPDDQEKRKFVEELIAYSDTTFVPEVYKSFAKDARTQAGVQFDYLEKALHKFDDGPFFLGQLSQVDIIYAPFVERFHVFMPEGFNYDITTGRPKLAKWIEEMNNLDGYKQTKVLEQEKMVGYYKNRFLLVPTS*

>SlGSTL6

MASSSVQDLLPPSLDSTSQPPSLFDGTTRLYINYQCPYSQRVWITRNVKGLQDMIKLVPIDLQNRPDWYKEKVYPKNKVPSLEHNNKVIGESFVLVKYVDYNFEGPSFMPDDQEKQKFAEELIAYSDTTFVPEVYRSFAKDARKLAGAQFDYLEKALHKFDDGPFFLGQFSQVDIIYAPFIERFHVFMPEGFNYDITTGRPKLAKWIEEMNNLDGYKQTKVLEQEKMVEYYKNRFLPKA*

>SlGSTL7

MGIFFYSDRSVKHLLPPSLDSTSQPPSLFDGTTRLYMNYQCPYSQHVWITRNVKGLQDMIKLVPINQQNRPDWYKEKVYPKNKVPSLKHNNKVIRESLVLVKYVDCNFEGPSFMPDKYTDLSQMMHGNWLVRIDHVFVETHHVLFVKCFLCKLTVLTLVLGAQFDYLEKALHKFDDGPFFLGQFSQGFNYDITTERPKLAKWIEEMNNLDGYKQTKVLEQEKMVEYYKNRFLPKA*

>SlGSTZ1

MKINPPMLLQISRSYIKLVKADNFQLWFGRHFSNTVTPTTNSIVDVSLAVPISGSSMESDNSMKKATDSTWVSKIVLYSFWQSSCSWRVRFALNLKGLSYEYRAVNLGKGEQFTSEFDKLNPLHYVPVLVDGDVVISDSYAILLYLEEKYHQRPLLPIKPQLRALNLQAASIVSSNMQPLHMLSVLRYMEERVGPEEKQLWAKFHIQKGFGALEKLLTGSAGKYATGDEVYMADVFLAPQIAVATKRFDIDMSEFPTLRKIYDSCEALPEFQASLPERQPDASP*

>SlGSTZ2

MAGSGEESKKLQLYSYWRSSCAFRVRIALNLKGLDYEYKAVNLLKGEQRDPEYLKLNPLGYVPTLVDGDAVIADSFAILMYLEEKYPQRALLPQDCQKRAINYQAANIVSANIQPLQNLAVLKYIQEKIGPDETTPWVQGHITKGFEALEKLLKDYAGKYATGDEVYMADLFLAPQIHAAIKRFEVDMNQFPTLLRVFEAYQELPAFQDAMPEKQPDAIHHL*

>SlDHAR1

MAVLRFPSLFYLGLLLRGSKIFSTFVSFMKSKDSSDGTEQALLDELKALEEHLKVHGPYVDGKNVCSVHMILAPKLYHLEVALGHFKKWSVPESLSHVRNYMNDFLGSGANFPVGNVSCFLFYSRHVAAFVIAEMKIVLFI*

>SlDHAR2

MVVEVCVKAAVGAPDVLGDCPFSQRVLLTLEEKKVTYKKHLINVSDKPKWFLEVNPEGKVPVINFGDKWIPDSDVIVGIIEEKYPNPSLIAPPEFASVGSKIFPTFVSFLKSKDSSDSTEQALLDELKALEEHLKAHGPYINGQNVCSVDMSLAPKLYHLEVALGHFKKWSVPESLSHVRNYMKLLFERESFQKTKAEEKYVIAGWAPKV*

>SlDHAR3

MESKDSSDCTEQALFDELKALEEHLKAHGPYVNGQNVCSVDMSLAPKLYHLKVALGHFKKWSVTESLTHVRNYMKDFVGNMSCFLFYSRDVAASVVVEMKIVFIYLTHWP*

>SlDHAR4

MKSKDSSDCTEHALFDELKALEEHLKAHGPYVNGQNVCSVDMSLAPKLCHLEVALGNFKKWSVTESLSHVRNYMKNDIKSAFWSMLLCYIEQHVSHSLFSSMQ*

>SlDHAR5

MSTAKITPSAASFATSIKHLAGIQLPRCQSTIFTSNSTKFRAPRRGFTVSMAASIETPLEVCVKQSITTPNKLGDCPFTQRVLLTLEEKHLPYDMKFVDLSNKPDWFLKISPEGKVPLIKLDEKWVPDSDVISQALEEKFPKPPLTTPPEKASVGSKIFPKFVAFLKSKDSGDGTEQALLDELTAFNDYLKENGPFINGNEVSAADLSLGPKLYHLEIALGNYKNWSIPDSLSYMKSYMKSIFSRESFINTRALKEDVIEGWRPKVMG*

>SlDHAR6

MKSKDSSDCTVQALFDELKALEEHLKAHGPYVNGQNVYSVDMSLAPKLYHLEVALEHFKKWSVTECLSHVRNYMKLDPFLLPVVQGSILVIQLNT

>SlEF1Bγ1

MALILHSTDNNKNASKALIAAEYTGVKVDLAKDFQMGVSNKTPEFLEMNPIGKVPVLQTPDGPVFESNAIARYVTKTKPNNPLFGSSLIEYAQIEQWNDFSATEIDANIARWLYPRLGYAVYISQAEEGAVAALKRALGALNTHLASNTYLVGHFITLADIIMVCNLSIGFRMILTKSFTKEFPHVERYFWTVVNQPNFVKILGEVKQAESIPAVQSKPAQPEKLKAKEEPKKEVKKEEPSPVEEEAAPKPKAKNPLDLLPPSKMILDDWKRLYSNTKTNFREVAVKGFWDMYDPEGYSLWFCDYKYNDENTVSFVTLNKVGGFLQRMELVRKYAFGKMLIVGSEAPFKVQGLWLFRGKEIPKFVMEEVYDMELYEWKEVDINDEAQKERVSQMIEDHEPFEGQALLDAKCFK*

>SlEF1Bγ2

MDLFLKAMLSHSTVRPCLIFFHLSSHHIAKYSFDTDFTNSRSYQINAQQSSFGSSLFEYSEEAAVSALKRALGTLNTHLASTKYLVEHLITLADNIVVCNLSIGFRMIMTKSFTKEIPRVERYFWTVVNQQNFSKILGKVKQAKSILAVQSKKPTQLEKTKANEEPTKEVNKEEPSLVEKETTPKPKAKNPLDLLPPSKISG*

>SlEF1Bγ3

MLQILHSTNNNKNASKALIAAEYTGVKVEVPKDFQMGVSNNTPEFLKMNPIGKVPVLETPDGPVFESNAIARYVTKLKPNNPLFGSSLIEYSQIEQWNDFSATEVDANIGRWLYPRLGFRVYIPAAEEAAVAALKRALGALNTHLASNTYLVGHSITLADIIMVCNLSIGFRMIMTKSFTKEFPHVERYFWTVVNQPNFCKILREVKQAESIPAVQSKKPAQPEKPKAKEELKKEVKKEEPSPVEEEAAPKPKAKNPLDLLPPSKMILDDWKRLYSNTKTNFREVAVKGFWDMYDPEGYSLWFCDYKYNDENTVSFVTLNKVGGFLQRMDLVRKYAFGKMLIVGSEAPFKVQGLWLFRGKEIPMFVMEEVYDMELYEWKEVDINDEAQKERVSQMIEDHEPFEGEALLDAKCFK*

>SlTCHQD

MQLYHHPFSLNSQKVRLTLEEKGIDYTSHHVNPLTGKNMDAFFFSMNPSAKVPVFQNGSHIIYDTIEIIQYIERIAEKVSSGGNNLNLSSREVIGWMHKIQEWDSMYFTLFHVPEKYRLCVSKFLRRVIIARMAESPDLASAYHCKLRQAYDTDDKLKNADVLRRSENHLVRLLDEVELKLGETSYLAGEEFSLADVMLIPLLARIELLNLENEYINSRPNIADYWVLVKQRPSYKKVIGKYFDGWRRRKTLLKTWCFIRVRSVLRKY*

>SlMGST1

MAGVEFLPKEYGYVILALVVYCFFNFWMSFQVGKARKQYKVPYPTMYATEAENKNANSFNCVQRGHQNSLEMMPTFFMLMIVGGIRHPLICASLGAVYIVSRYFYFTGYSTGDPQNRLTLGKYNFLAIMALMICAASCGVNFLMS*

>SlMGST2

MTSWAEIFPEPTAPGAQFKLLTWQSLKPSLNPKLSCLQIATKAHRIKVCIFQIFGRGKRIAGMRRVSRIAALYRAVDGAAAMEVPQHRMSTAAQFSTSSNKSSTRSNWLFNNLLTDLSARTSAHAVAGTMLFSVAATTLTEEVHAKEVVPPELRPKDLVLYQYEACPFCNKVKAFLDYYDLPYKIIEVNPISKKELKWSDYKKVPVVLVDGEQMVNSSDIIDKLYEKVRSGDSTFDADEESKWRKWVDDHLVHMLSPNIYRNTSEALESFDYITSHGNFSFTERITAKYAGAAAMYFVSKKLKKKYNITDERAALYEAAETWVDALKGRDFLGGSKPNLADLAVYGVLRPIRYLKSGRDMVENTRIGDWYSRMESEVGVSARIQA*

>SlGHR1

MSATGAFERTASTFRNIVSREPGSVFPVESGRYHLYISYACPWASRCLAYLKIKGLDQAIDFTSVKPVWERTKDSDEHTGWVFASSSTEEAGADLDPLNGAKSIRELYELASTNYSGKYTVPVLWDKKLKTIVNNESAEIIRMFNSEFNDIAENAALDLYPPHLQSLINEANEWIYDGINNGVYQCGFAKKQEPYDEAVQKVYKALDKCEEILSKQRYICGDQVTEADIRLFVTLIRFDEVYAVYFKCNKKLLREYPNLFNYTKDIFQIPGMSSTVNMEHIKKHYYRSHPSINPFGIIPQGPNIDYSSPHDREKFSK*

>SlGHR2

MYSAQVSSFISIPFPPSKSKTHKLKYPKILHTKLCNSTPKMSLNQNSNTNLINTITKLLWGPSLPPQLLISTVRSTWSATWQLMMSQLAPSDPTGSYTRPTSQFRLYSNPELKFSPKDLHLYVGLPCPWAHRTLIVRALKGLEDSVPVSIASPGIDGSWEFRVFSDPDKDKLVPGLDKANGCKTLREVYKLRRGGYSGRSTVPMLWDMGKKEVLCNESYDIIEFFNSGLNEISGNPELDLSPPALKVDIRKWNDIIYPNVNNGVYRCGFAQSQEAYNKAAEGLFRTLEMLEDHLAGSRYLCGDVLTLADVCLFTTLIRFDVVYNVLFKCTKKKLIEFTNLHGYLRDIYQIPKVAETCNMGQIMEGYYKILFPLNPGGINPIMPSGCEDEVLSKPHNRDCLSLETKVVQHSVS*

>ATGSTF7

MAGIKVFGHPASTATRRVLIALHEKNLDFEFVHIELKDGEHKKEPFIFRNPFGKVPAFEDGDFKLFESRAITQYIAHFYSDKGNQLVSLGSKDIAGIAMGIEIESHEFDPVGSKLVWEQVLKPLYGMTTDKTVVEEEEAKLAKVLDVYEHRLGESKYLASDKFTLVDLHTIPVIQYLLGTPTKKLFDERPHVSAWVADITSRPSAKKVL

>ATGSTF6

MAGIKVFGHPASTATRRVLIALHEKNVDFEFVHVELKDGEHKKEPFILRNPFGKVPAFEDGDFKIFESRAITQYIAHEFSDKGNNLLSTGKDMAIIAMGIEIESHEFDPVGSKLVWEQVLKPLYGMTTDKTVVEEEEAKLAKVLDVYEHRLGESKYLASDHFTLVDLHTIPVIQYLLGTPTKKLFDERPHVSAWVADITSRPSAQKVL

>ATGSTF5

MGINASHVPETCYHHCNQTFESSRQCFKWCQELARKDEYKIYGYPYSTNTRRVLAVLHEKGLSYDPITVNLIAGDQKKPSFLAINPFGQVPVFLDGGLKLTESRAISEYIATVHKSRGTQLLNYKSYKTMGTQRMWMAIESFEFDPLTSTLTWEQSIKPMYGLKTDYKVVNETEAKLEKVLDIYEERLKNSSFLASNSFTMADLYHLPNIQYLMDTHTKRMFVNRPSVRRWVAEITARPAWKRACDVKAWYHKKKN

>ATGSTF4

MDCLQMVFKLFPNWKREAEVKKLVAGYKVHGDPFSTNTRRVLAVLHEKRLSYEPITVKLQTGEHKTEPFLSLNPFGQVPVFEDGSVKLYESRAITQYIAYVHSSRGTQLLNLRSHETMATLTMWMEIEAHQFDPPASKLTWEQVIKPIYGLETDQTIVKENEAILEKVLNIYEKRLEESRFLACNSFTLVDLHHLPNIQYLLGTPTKKLFEKRSKVRKWVDEITSREAWKMACDQEKSWFNKPRN

>ATGSTU18

MATEDVKLIGSWASVYVMRARIALHLKSISYEFLQETYGSKSELLLKSNPVHKKMPVLIHADKPVCESNIIVHYIDEAWNSSGPSILPSHPYDRAIARFWAAYIDDQWFISVRSILTAQGDEEKKAAIAQVEERTKLLEKAFNDCSQGKPFFNGDHIGYLDIALGSFLGWWRVVELDANHKFLDETKTPSLVKWAERFCDDPAVKPIMPEITKLAEFARKLFPKRQA

>ATGSTU17

MASSDVKLIGAWASPFVMRPRIALNLKSVPYEFLQETFGSKSELLLKSNPVHKKIPVLLHADKPVSESNIIVEYIDDTWSSSGPSILPSDPYDRAMARFWAAYIDEKWFVALRGFLKAGGEEEKKAVIAQLEEGNAFLEKAFIDCSKGKPFFNGDNIGYLDIALGCFLAWLRVTELAVSYKILDEAKTPSLSKWAENFCNDPAVKPVMPETAKLAEFAKKIFPKPQA

>ATGSTU24

MADEVILLDFWASMFGMRTRIALAEKRVKYDHREEDLWNKSSLLLEMNPVHKKIPVLIHNGKPVCESLIQIEYIDETWPDNNPLLPSDPYKRAHAKFWADFIDKKVNVTARRIWAVKGEEQEAAKELIEILKTLESELGDKKYFGDETFGYVDIALIGFHSWFAVYEKFGNVSIESECSKLVAWAKRCLERESVAKALPESEKVITFISERRKKLGLE

>ATGSTU25

MADEVILLDFWPSMFGMRTRIALEEKNVKFDYREQDLWNKSPILLEMNPVHKKIPVLIHNGNPVCESLIQIEYIDEVWPSKTPLLPSDPYQRAQAKFWGDFIDKKVYASARLIWGAKGEEHEAGKKEFIEILKTLESELGDKTYFGGETFGYVDIALIGFYSWFEAYEKFGSFSIEAECPKLIAWGKRCVERESVAKSLPDSEKIIKFVPELRKKLGIEIE

>ATGSTU26

MANDQVILLDYWPSMFGMRTKMALAEKGVKYEYKETDPWVKTPLLIEMNPIHKKIPVLIHNGKPICESLIQLEYIDEVWSDASPILPSDPYQKSRARFWAEFIDKKFYDPSWKVWATMGEEHAAVKKELLEHFKTLETELGDKPYYGGEVFGYLDIALMGYYSWFKAMEKFGEFSIETEFPILTTWTKRCLERESVVKALADSDRIIEYVYVLRKKFGAA

>ATDHAR1

MALEICVKAAVGAPDHLGDCPFSQRALLTLEEKSLTYKIHLINLSDKPQWFLDISPQGKVPVLKIDDKWVTDSDVIVGILEEKYPDPPLKTPAEFASVGSNIFGTFGTFLKSKDSNDGSEHALLVELEALENHLKSHDGPFIAGERVSAVDLSLAPKLYHLQVALGHFKSWSVPESFPHVHNYMKTLFSLDSFEKTKTEEKYVISGWAPKVNP

>ATGSTU13

MAQNDTVKLIGSWSSPYSLRARVALHLKSVKYEYLDEPDVLKEKSELLLKSNPIHKKVPVLLHGDLSISESLNVVQYVDEAWPSVPSILPSDAYDRASARFWAQYIDDKCFAAVDAVVGAKDDEGKMAAVGKLMECLAILEETFQKSSKGLGFFGGETIGYLDIACSALLGPISVIEAFSGVKFLRQETTPGLIKWAERFRAHEAVKPYMPTVEEVVAFAKQKFNVQ

>ATGSTU14

MAQNDTVKLIGCSDDPFSIRPRVALHLKSIKYEYLEEPDDDLGEKSQLLLKSNPIHKKTPVLIHGDLAICESLNIVQYLDEAWPSDPSILPSNAYDRASARFWAQYIDDKCFEAANALTGANNDEERIAATGKLTECLAILEETFQKSSKGLGFFGGETIGYLDIACAALLGPISVIEMFSADKFVREETTPGLIQWAVRFRAHEAVRPYMPTVEEVTELVKQRIEEGFKRNFKSNVSTSEYE

>ATGSTF14

MADSKMKLHCGFIWGNSAALFCINEKGLDFELVFVDWLAGEAKTKTFLSTLNPFGEVPVLEDGDLKLFEPKAITRYLAEQYKDVGTNLLPDDPKKRAIMSMWMEVDSNQFLPIASTLIKELIINPYQGLATDDTAVQENKEKLSEVLNIYETRLGESPYLAGESFSLADLHHLAPIDYLLNTDEEELKNLIYSRPNVAAWVEKMKMRPAWLKTVVMKNHIVDLMKQRRLPIKLDSSCHESTVVAQKNAIAIENK

>ATGSTU28

MGKENSKVVVLDFWASPYAMRTKVALREKGVEFEVQEEDLWNKSELLLKSNPVHKKVPVLIHNNTPISESLIQVQYIDETWTDAASFLPSDPQSRATARFWADYADKTISFEGGRKIWGNKKGEEQEKGKKEFLESLKVLEAELGDKSYFGGETFGYVDITLVPFYSWFYALEKCGDFSVEAECPKIVAWGKRCVERNSVAATLPESEKVYQQVLKLRQIFGVE

>ATGSTU15

MGEREEVKLLGTWYSPVVIRAKIALRLKSVDYDYVEEDLFGSKSELLLKSNPIFKKVPVLIHNTKPVCVSLNIVEYIDETWNSSGSSILPSHPYDRALARFWSVFVDDKWLPTLMAAVVAKSEEAKAKGMEEVEEGLLQLEAAFIALSKGKSFFGGETIGFIDICLGSFLVLLKAREKLKNEKILDELKTPSLYRWANQFLSNEMVKNVVPDIDKVAKFIEEFEDRAQYIRCF

>ATGSTU16

MGEKEEVKLLGVWYSPYAIRPKIALRLKSVDYDYVEENLFGSKSELLLKSNPVHKKVPVLLHNNKPIVESLNIVEYIDETWNSSAPSILPSHPYDRALARFWSDFVDNKWFPALRMAAITKSEDAKAKAMEEVEEGLLQLEDAFVSISKGKPFFGGEAIGFMDICFGSFVVLLKAREKFKAEKLLDESKTPSLCKWADRFLSDETVKNVAPEIEKVAEFLQELEVRAQSAASRS

>ATGSTU12

MLKNKKSDNSLSRDTLQIKKRKKTTMAQNGSNTTVKLIGTWASPFAIRAQVALHLKSVEHEYVEETDVLKGKSDLLIKSNPIHKKVPVLIHGDVSICESLNIVQYVDESWPSDLSILPTLPSERAFARFWAHFVDGKLFESIDAVAGAKDDAARMTLAGNLMENLAALEEAFQKSSKGGDFFGGGNIGFVDITVGAIVGPISVIEAFSGVKFLRPDTTPGLIQWAEKFRAHEAVKPYMPTVAEFIEFAKKKFSV

>ATGSTU11

MGLMNRSKNDEYVKLLGAWPSPFVLRTRIALNLKNVAYEYLEEEDTLSSESVLNYNPVHKQIPILIHGNKPIRESLNIVMYVDETWLSGPPILPSDPFDRAVARFWDVYIDEHCFTSINGVAVAKGEENINAAIAKLEQCMALLEETFQECSKGRGFFGGENIGFIDIGFGSMLGPLTVLEKFTGVKFIHPENTPGLFHWADRFYAHEAVKPVMPDIEKLVQFARLKFNTSIFK

>ATGSTU10

MEEKKSKVILHGTWISTYSKRVEIALKLKGVLYEYLEEDLQNKSESLIQLNPVHKKIPVLVHDGKPVAESLVILEYIDETWTNSPRFFPEDPYERAQVRFWVSYINQQVFEVMGQVMSQEGEAQAKSVEEARKRFKVLDEGLKKHFPNKNIRRNDDVGLLEITIIATLGGYKAHREAIGVDIIGPVNTPTLYNWIERLQDLSVIKEVEVPHDTLVTFIQKYRQKCLQQAANA

>AtDHAR2

MALDICVKVAVGAPDVLGDCPFSQRVLLTLEEKKLPYKTHLINVSDKPQWFLDISPEGKVPVVKLDGKWVADSDVIVGLLEEKYPEPSLKTPPEFASVGSKIFGAFVTFLKSKDANDGSEKALVDELEALENHLKTHSGPFVAGEKITAVDLSLAPKLYHLEVALGHYKNWSVPESLTSVRNYAKALFSRESFENTKAKKEIVVAGWESKVNA

>AtTCHQD1

MQLYHHPYSIDSQRVRLALEEKGIDYTSYHVNPITGKHMDPSFFRMNPNAKLPVFRNGSHIILDTIEIIEYLERIAEVSSGIEDATFNREVVEWMRKIREWESKLFTLAHIPDNRRLYVSKFLRMVVIARMAESPDLASAYHRKLREAYDTEDKLKDPGALRRSKDHLLRLLDEVETKLEGTTYLAGNEFSMADVMLIPVLARLSLLDLEEEYISSRKNLAEYWALVRRRPSYKKVIGRYFNGWRKYATLVKTWMFVRVRSLLRKY

>AtGSTU23

MEEEIILLDYWASMYGMRTRIALEEKKVKYEYREEDLSNKSPLLLQMNPIHKKIPVLIHEGKPICESIIQVQYIDELWPDTNPILPSDPYQRAQARFWADYIDKKTYVPCKALWSESGEKQEAAKIEFIEVLKTLDSELGDKYYFGGNEFGLVDIAFIGFYSWFRTYEEVANLSIVLEFPKLMAWAQRCLKRESVAKALPDSDKVLKSVSDHRKIILGID

>AtGSTU22

MADEVILLDFWPSPFGVRARIALREKGVEFEYREENLRDKSPLLLQMNPVHKKIPVLIHNGKPVCESMNVVQYIDEVWSDKNPILPSDPYQRAQARFWVDFVDTKLFEPADKIWQTKGEEQETAKKEYIEALKILETELGDKPYFGGDTFGFVDIAMTGYYSWFEASEKLANFSIEPECPTLMASAKRCLQRESVVQSLHDSEKILAFAYKIRKIYCV

>AtGSTU21

MAAEVILLGFWPSMFGMRTMIALEEKGVKYEYREEDVINNKSPLLLEMNPIHKTIPVLIHNGKPVLESLIQIQYIDEVWSDNNSFLPSDPYHRAQALFWADFIDKKEQLYVCGRKTWATKGEELEAANKEFIEILKTLQCELGEKPYFGGDKFGFVDIVLIGFYSWFPAYQKFGNFSIEPECLKLIAWGKRCMQRESVAKALPDSEKVVGYVLQLKKLYGIE

>AtGSTU20

MANLPILLDYWPSMFGMRARVALREKGVEFEYREEDFSNKSPLLLQSNPIHKKIPVLVHNGKPVCESLNVVQYVDEAWPEKNPFFPSDPYGRAQARFWADFVDKKFTDAQFKVWGKKGEEQEAGKKEFIEAVKILESELGDKPYFGGDSFGYVDISLITFSSWFQAYEKFGNFSIESESPKLIAWAKRCMEKESVSKSLPDSEKIVAYAAEYRKNNL

>AtGSTU19

MANEVILLDFWPSMFGMRTRIALREKGVEFEYREEDLRNKSPLLLQMNPIHKKIPVLIHNGKPVNESIIQVQYIDEVWSHKNPILPSDPYLRAQARFWADFIDKKLYDAQRKVWATKGEEQEAGKKDFIEILKTLESELGDKPYFSGDDFGYVDIALIGFYTWFPAYEKFANFSIESEVPKLIAWVKKCLQRESVAKSLPDPEKVTEFVSELRKKFVPE

>AtGSTZ2

MSYVTDFYQAKLKLYSYWRSSCAHRVRIALTLKGLDYEYIPVNLLKGDQSDSDFKKINPMGTVPALVDGDVVINDSFAIIMYLDDKYPEPPLLPSDYHKRAVNYQATSIVMSGIQPHQNMALFRYLEDKINAEEKTAWITNAITKGFTALEKLLVSCAGKYATGDEVYLADLFLAPQIHAAFNRFHINMEPFPTLARFYESYNELPAFQNAVPEKQPDTPSTI

>AtGSTZ1

MANSGEEKLKLYSYWRSSCAHRVRIALALKGLDYEYIPVNLLKGDQFDSVYRFDLQDFKKINPMGTVPALVDGDVVINDSFAIIMYLDEKYPEPPLLPRDLHKRAVNYQAMSIVLSGIQPHQNLAVIRYIEEKINVEEKTAWVNNAITKGFTALEKLLVNCAGKHATGDEIYLADLFLAPQIHGAINRFQINMEPYPTLAKCYESYNELPAFQNALPEKQPDAPSSTI

>AtGSTF3

MAGIKVFGHPASTSTRRVLIALHEKNLDFELVHVELKDGEHKKEPFLSRNPFGQVPAFEDGDLKLFESRAITQYIAHRYENQGTNLLPADSKNIAQYAIMSIGIQVEAHQFDPVASKLAWEQVFKFNYGLNTDQAVVAEEEAKLAKVLDVYEARLKEFKYLAGETFTLTDLHHIPVIQYLLGTPTKKLFTERPRVNEWVAEITKRPASEKVL

>AtGSTU7

MAERSNSEEVKLLGMWASPFSRRIEIALTLKGVSYEFLEQDITNKSSLLLQLNPVHKMIPVLVHNGKPISESLVILEYIDETWRDNPILPQDPYERTMARFWSKFVDEQIYVTAMKVVGKTGKERDAVVEATRDLLMFLEKELVGKDFLGGKSLGFVDIVATLVAFWLMRTEEIVGVKVVPVEKFPEIHRWVKNLLGNDVIKKCIPPEDEHLKYIRARMEKLNIKSA

>AtGSTU6

MGKNEEVKLLGIWASPFSRRIEMALKLKGVPYEYLEEDLENKSSLLLALSPIHKKIPVLVHNGKTIIESHVILEYIDETWKHNPILPQDPFQRSKARVLAKLVDEKIVNVGFASLAKTEKGREVLIEQTRELIMCLEKELAGKDYFGGKTVGFLDFVAGSMIPFCLERAWEGMGVEMITEKKFPEYNKWVKKLKEVEIVVDCIPLREKHIEHMNNMAEKIRSA

>AtGSTU5

MAEKEEVKLLGIWASPFSRRVEMALKLKGIPYEYVEEILENKSPLLLALNPIHKKVPVLVHNGKTILESHVILEYIDETWPQNPILPQDPYERSKARFFAKLVDEQIMNVGFISMARADEKGREVLAEQVRELIMYLEKELVGKDYFGGKTVGFLDFVAGSLIPFCLERGWEGIGLEVITEEKFPEFKRWVRNLEKVEIVKDCVPPREEHVEHMNYMAERVRSS

>AtGSTU4

MAEKEEDVKLLGFWASPFTRRVEMAFKLKGVPYEYLEQDIVNKSPLLLQINPVYKKVPVLVYKGKILSESHVILEYIDQIWKNNPILPQDPYEKAMALFWAKFVDEQVGPVAFMSVAKAEKGVEVAIKEAQELFMFLEKEVTGKDFFGGKTIGFLDLVAGSMIPFCLARGWEGMGIDMIPEEKFPELNRWIKNLKEIEIVRECIPPREEQIEHMKKVVERIKSA

>AtGSTU3

MAEKEEGVKLIGSWASPFSRRVEMALKLKGVPYDYLDEDYLVVKSPLLLQLNPVYKKVPVLVHNGKILPESQLILEYIDQTWTNNPILPQSPYDKAMARFWAKFVDEQVTMIGLRSLVKSEKRIDVAIEEVQELIMLLENQITGKKLFGGETIGFLDMVVGSMIPFCLARAWEGMGIDMIPEEKFPELNRWIKNLKEIEIVRECIPDREKHIEHMMKIVGRIKAV

>AtGSTU2

MAKKEESVKLLGFWISPFSRRVEMALKLKGVPYEYLEEDLPKKSTLLLELNPVHKKVPVLVHNDKLLSESHVILEYIDQTWNNNPILPHDPYEKAMVRFWAKFVDEQILPVGFMPLVKAEKGIDVAIEEIREMLMFLEKEVTGKDFFGGKTIGFLDMVAGSMIPFCLARAWECLGIDMTPEDTFPELNRWIKNLNEVEIVRECIPPKEKHIERMKKIIERAKSTF

>AtGSTU1

MAEKEESVKLLGFWASPFSRRVEMALKLKGVPYEYLEEDLPNKTPLLLELNPLHKKVPVLVHNDKILLESHLILEYIDQTWKNSPILPQDPYEKAMARFWAKFIDDQILTLGFRSLVKAEKGREVAIEETRELLMFLEKEVTGKDFFGGKTIGFLDMIAGSMIPFCLARLWKGIGIDMIPEEKFPELNRWIKNLEEVEAVRGCIPPREKQIERMTKIAETIKSA

>AtGSTF9

MVLKVYGPHFASPKRALVTLIEKGVAFETIPVDLMKGEHKQPAYLALQPFGTVPAVVDGDYKIFESRAVMRYVAEKYRSQGPDLLGKTVEDRGQVEQWLDVEATTYHPPLLNLTLHIMFASVMGFPSDEKLIKESEEKLAGVLDVYEAHLSKSKYLAGDFVSLADLAHLPFTDYLVGPIGKAYMIKDRKHVSAWWDDISSRPAWKETVAKYSFPA

>AtGSTF10

MVLTIYAPLFASSKRAVVTLVEKGVSFETVNVDLMKGEQRQPEYLAIQPFGKIPVLVDGDYKIFESRAIMRYIAEKYRSQGPDLLGKTIEERGQVEQWLDVEATSYHPPLLALTLNIVFAPLMGFPADEKVIKESEEKLAEVLDVYEAQLSKNEYLAGDFVSLADLAHLPFTEYLVGPIGKAHLIKDRKHVSAWWDKISSRAAWKEVSAKYSLPV

>AtGSTF8

MGAIQARLPLFLSPPSIKHHTFLHSSSSNSNFKIRSNKSSSSSSSSIIMASIKVHGVPMSTATMRVLATLYEKDLQFELIPVDMRAGAHKQEAHLALNPFGQIPALEDGDLTLFESRAITQYLAEEYSEKGEKLISQDCKKVKATTNVWLQVEGQQFDPNASKLAFERVFKGMFGMTTDPAAVQELEGKLQKVLDVYEARLAKSEFLAGDSFTLADLHHLPAIHYLLGTDSKVLFDSRPKVSEWIKKISARPAWAKVIDLQKQ

>AtGSTF11

MVVKVYGQIKAANPQRVLLCFLEKDIEFEVIHVDLDKLEQKKPQHLLRQPFGQVPAIEDGYLKLFESRAIARYYATKYADQGTDLLGKTLEGRAIVDQWVEVENNYFYAVALPLVMNVVFKPKSGKPCDVALVEELKVKFDKVLDVYENRLATNRYLGGDEFTLADLSHMPGMRYIMNETSLSGLVTSRENLNRWWNEISARPAWKKLMELAAY

>AtGSTU8

MNQEEHVKLLGLWGSPFSKRVEMVLKLKGIPYEYIEEDVYGNRSPMLLKYNPIHKKVPVLIHNGRSIAESLVIVEYIEDTWKTTHTILPQDPYERAMARFWAKYVDEKVMLAVKKACWGPESEREKEVKEAYEGLKCLEKELGDKLFFGGETIGFVDIAADFIGYWLGIFQEASGVTIMTAEEFPKLQRWSEDFVGNNFIKEVLPPKEKLVAVLKAMFGSVTSN

>AtGSTU27

MSEEEVVVLNFWPSMFGARVIMALEEKEIKFEYKEEDVFGQKTDLLLQSNPVNKKIPVLIHNGKPVCESNIIVEYIDEVWKDDKTLRLLPSDPYQKSQCRFWADLIDKKVFDAGRRTWTKRGKEQEEAKQEFIEILKVLERELGDKVYFGGNDNVSMVDLVLISYYPWFHTWETIGGFSVEDHTPKLMDWIRKCLTRPAISKSLPDPLKIFDRVTQIIKVHEFFYGY

>AtGSTL2

MSVGLKVSAFLHPTLALSSRDVSLSSSSSSLYLDRKILRPGSGRRWCKSRRTEPILAVVESSRVPELDSSSEPVQVFDGSTRLYISYTCPFAQRAWIARNYKGLQNKIELVPIDLKNRPAWYKEKVYSANKVPALEHNNRVLGESLDLIKYIDTNFEGPSLTPDGLEKQVVADELLSYTDSFSKAVRSTLNGTDTNAADVAFDYIEQALSKFNEGPFFLGQFSLVDVAYAPFIERFRLILSDVMNVDITSGRPNLALWIQEMNKIEAYTETRQDPQELVERYKRRVQAEARL

>AtGSTF13

MAMKLYGDEMSACVARVLLCLHEKNTEFELVPVNLFACHHKLPSFLSMNPFGKVPALQDDDLTLFESRAITAYIAEKHRDKGTDLTRHEDPKEAAIVKLWSEVEAHHFNPAISAVIHQLIVVPLQGESPNAAIVEENLENLGKILDVYEERLGKTKYLAGDTYTLADLHHVPYTYYFMKTIHAGLINDRPNVKAWWEDLCSRPAFLKVSPGLTVAPTTN

>AtGSTF2

MAGIKVFGHPASIATRRVLIALHEKNLDFELVHVELKDGEHKKEPFLSRNPFGQVPAFEDGDLKLFESRAITQYIAHRYENQGTNLLQTDSKNISQYAIMAIGMQVEDHQFDPVASKLAFEQIFKSIYGLTTDEAVVAEEEAKLAKVLDVYEARLKEFKYLAGETFTLTDLHHIPAIQYLLGTPTKKLFTERPRVNEWVAEITKRPASEKVQ

>AtGSTL1

MALSPPKIFVEDRQVPLDATSDPPALFDGTTRLYISYTCPFAQRVWITRNLKGLQDEIKLVPIDLPNRPAWLKEKVNPANKVPALEHNGKITGESLDLIKYVDSNFDGPSLYPEDSAKREFGEELLKYVDETFVKTVFGSFKGDPVKETASAFDHVENALKKFDDGPFFLGELSLVDIAYIPFIERFQVFLDEVFKYEIIIGRPNLAAWIEQMNKMVAYTQTKTDSEYVVNYFKRFM

>AtGSTL3

MAPSFIFVEDRPAPLDATSDPPSLFDGTTRLYTSYVCPFAQRVWITRNFKGLQEKIKLVPLDLGNRPAWYKEKVYPENKVPALEHNGKIIGESLDLIKYLDNTFEGPSLYPEDHAKREFGDELLKYTDTFVKTMYVSLKGDPSKETAPVLDYLENALYKFDDGPFFLGQLSLVDIAYIPFIERFQTVLNELFKCDITAERPKLSAWIEEINKSDGYAQTKMDPKEIVEVFKKKFM

>AtDHAR3

MISLRFQPSTTAGVLSASVSRAGFIKRCGSTKPGRVGRFVTMATAASPLEICVKASITTPNKLGDCPFCQKVLLTMEEKNVPYDMKMVDLSNKPEWFLKISPEGKVPVVKFDEKWVPDSDVITQALEEKYPEPPLATPPEKASVGSKIFSTFVGFLKSKDSGDGTEQVLLDELTTFNDYIKDNGPFINGEKISAADLSLAPKLYHMKIALGHYKNWSVPDSLPFVKSYMENVFSRESFTNTRAETEDVIAGWRPKVMG

>AtGSTF12

MVVKLYGQVTAACPQRVLLCFLEKGIEFEIIHIDLDTFEQKKPEHLLRQPFGQVPAIEDGDFKLFESRAIARYYATKFADQGTNLLGKSLEHRAIVDQWADVETYYFNVLAQPLVINLIIKPRLGEKCDVVLVEDLKVKLGVVLDIYNNRLSSNRFLAGEEFTMADLTHMPAMGYLMSITDINQMVKARGSFNRWWEEISDRPSWKKLMVLAGH

>AtGSTT1

MMKLKVYADRMSQPSRAVIIFCKVNGIQFDEVLISLAKRQQLSPEFKDINPLGKVPAIVDGRLKLFESHAILIYLSSAFPSVADHWYPNDLSKRAKIHSVLDWHHTNLRRGAAGYVLNSVLGPALGLPLNPKAAAEAEQLLTKSLSTLETFWLKGNAKFLLGSNQPSIADLSLVCELMQLQVLDDKDRLRLLSTHKKVEQWIENTKKATMPHFDETHEILFKVKEGFQKRREMGTLSKPGLQSKI

>AtGSTT2

MKLKVYADRMSQPSRAVLIFCKVNEIQFDEILISLGKRQQLSPEFKEINPMGKVPAIVDGRLKLFESHAILIYLSSAYASVVDHWYPNDLSKRAKIHSVLDWHHTNLRPGASGYVLNSVLAPALGLPLNPKAAAEAENILTNSLSTLETFWLKGSAKFLLGGKQPSIADLSLVCELMQLQVLDDKDRLRLLSPHKKVEQWIESTRKATMPHSDEVHEVLFRAKDRFQKQREMATASKPGPQSKIIQFSSIGGTSDGPNLVQDTTDRKARRRKWSPPDDVILISAWLNTSKDRKVVVYDEQQAHTFWKRIGAHVSNSASLANLPKREWNHCRQRWRKINDYVCKFVGCYDQALNQRASGQSEDDVFQVAYQLYYNNYMSNFKLEHAWRELRHNKKWCSTYTSENSKGGGSSKRTKLNGGGVYSSSCNPESVPIALDGEEQVMDRPLGVKSSKQKEKKVATKTMLEEREADSRSRLENLWVLDEEEQVMDLPLGVKSSKQKERKVATKTMIEEREAANFRSRLGNLWLLKEKEEREADSRSRLENLWALKEKDIEEQKKLTRMEVLKSLLGRRTGETSEKEETLKNKLIDEML

>AtGSTU9

MDEEVENKVILHGSFASPYSKRIELALRLKSIPYQFVQEDLQNKSQTLLRYNPVHKKIPVLVHNGKPISESLFIIEYIDETWSNGPHILPEDPYRRSKVRFWANYIQLHLYDLVIKVVKSEGEEQKKALTEVKEKLSVIEKEGLKEIFSDTDGEPTVTNETMSLVDIVMCTLLSPYKAHEEVLGLKIIDPEIVPGVYGWI

>AtGHR1

MSYSTIISNTSFLSLASKFTTRGSRLQCTVSMARSAVDETSDSGAFQRTASTFRNFVSKDSNSQFPAESGRYHLYISYACPWASRCLSYLKIKGLDDAISFSSVKPIWGRTKETDEHMGWVFPGSDTEVPGADPDHLNGAKSVRELYEIASPNYTGKYTVPVLWDKKLKTVVNNESAEIIRMFNTEFNHIAGNPDLDLYPSHLQAKIDETNEWIYNGINNGVYRCGFAKKQGPYEEAVEQVYEALDRCEEILGKHRYICGNTLTETDIRLFVTLIRFDEVSSYFQSKKKKYTICERISNVETLIQVYAVHFKCNKKLIREYPNLFNYTKDIFQIPGMSSTVNMNHIKQHYYGSHPSINPFGIIPHGPNIDYTSPHDRHRFSK

>AtGHR2

MANCFAPQLTFPSFSPRHFSPRMSHQSPKPSTSTTTSIFTSATKLLWGPSLPPGLLISTARTAWTTVWQLMMTQLAPSDSSGSYTRPTSKFRLDPTQFTSAASSELHLYVGLPCPWAHRTLIVRALKGLNDAVPVSIASPGQDGSWEFKNNNIPIKDKDKLIPSLDKANRCRNLKEVYKSRSGGYDGRCTVPMLWDLRKKDVVCNESYDIIEFFNSGLNKLARNDNLDLSPPELKEMIQGWNQIVYPKVNNGVYRCGFAQSQEAYDGAVNELFSTLDEIEDHLGSNRYLCGERLTLADVCLFTTLIRFDSVYNILFKCTKKKLVEYPNLYGYLREIYQIPGVAATCDISAIMDGYYKTLFPLNASGIQPAISSSGDQDSLWRPHNRDLVGKAIEAQLSV

>AtGHR3

MATPMENENPNFARTATSFRNFVSKDPDSQFPAESGRYHLYISYACPWASRCLAILKLKGLDKAISFSSVQPLWRNTKENDEHMGWVFPDSDTEVLGAERDHINGAKSVRELYDIASSNYTGKYTVPVLWDKKLKTIVNNESSEILRMFNTEFNHVAENPSLDLYPPNLRAIIDETNEWIHDGINNGVYKCGFATNQETYDVEVKRLYEALDRCEDILRKQRFLCGNTLTESDIRLFVTVIRFDEAYAVIFKCDKRLVREYYHLFNYTKDIYQIAGMSSTVKMDHIKQNYYGSFPSINPLEIIAHGPNIDYSLPHDRHRFSLESDYTRLELFESASFVCELKLIEIFDSL

>AtGHR4

MARSGVDETSESGAFVRTASTFRNFVSQDPDSQFPAESGRYHLYISYACPWACRCLSYLKIKGLDEAITFSSVHAIWGRTKETDDHRGWVFPDSDTELPGAEPDYLNGAKSVRELYEIASPNYEGKYTVPVLWDKKLKTVVNNESSEIIRMFNTEFNGIAKTPSLDLYPSHLRDVINETNGWVFNGINNGVYKCGFARKQEPYNEAVNQLYEAVDRCEEVLGKQRYICGNTFTEADIRLFVTLIRFDEVYAVHFKCNKRLLREYPNIFNYIKDIYQIHGMSSTVNMEHIKQHYYGSHPTINPFGIIPHGPNIDYSSPHDRDRFSS

>OsGSTF1

MTPVKVFGPAQSTNVARVLLCLEEVGAEYEVVNVDFTVMEHKSPEHLKRNPFGQIPAFQDGDLYLFESRAIGKYILRKYKTREADLLREGNLREAAMVDVWTEVETHQYNSAISPIVYECIINPAMRGIPTNQKVVDESAEKLKKVLEVYEARLSQSTYLAGDFVSFADLNHFPYTFYFMGTPYASLFDSYPHVKAWWERLMARPSVKKLAAVMAPQGA*

>OsGSTF2

MAPMKLYGSTLSWNVTRCVAVLEEAGAEYEIVPLDFSKGEHKAPDHLARNPFGQVPALQDGDLFLWESRAICKYVCRKNKPELLKDGDLKESAMVDVWLEVESNQYTPALNPILFQCLIRPMMFGAPPDEKVVEENLEKLKKVLEVYEARLTKCKYLAGDYISVADLSHVAGTVCLGATPHASVLDAYPHVKAWWTDLMARPSSQKVASLMKPPA*

>OsGSTF3

MAAPVTVYGPMISPAVARVAACLLEKDVPFQVEPVDMSKGEHKSPSFLKLQPFGQVPAFKDSLTTVFESRAICRYICDQYADSGNKTLMGRKEDGAVGRAAIEKWIEAEGQSFNPPSLAMAFQLAFAPFMGRATDMAVVEQNEAKLVKVLDVYEQWLGENQYFAGDEFSLADLVHMPNTDLLVRKTNKAGLFTERKNLARWWDEVSARPSWKKVVELQNVPRPS*

>OsGSTF4

MAGEGRKLRVYGMALSANVVRVATVLNEKGLDFDLVPVDLRTAAHKQPHFLALNPFGQIPVLQDGDEVLYESRAINRYIATKYKAEGADLLPAEASPAKLEVWLEVESHHFYPAISGLVFQLLIKPLLGGATDTAAVDEHAAALAQVLDVYDAHLAGSRYLAGNRFSLADANHMSYLLFLSKTPMAELVASRPHVKAWWDDISSRPAWKKTAAAIPFPPAA*

>OsGSTF5

MYQQSAGQIQLQEGPMAPMKVYGWVVSPWMARVLVALEEAGAEYEVVPMSRSGGDHRRPEHLARNPFGEIPVLEDGDLTLYQSRAIARYIFRKYKPEFLGLGEGGSLEESAMVDVWLDVEAHQHEAAVRPILWHCIINKFEGRDRDQGVVDESVRKLEKVLGVYEARLSGSRYLAGDRISLADLSHFSNMRYFMATEYAGVVDAYPHVKAWWEALLARPTVQKVMAGMPPDFGFGSGNIP*

>OsGSTF6

MPGAVKVFGSPSSAEVARVLACLFEKDVEFQLIRVDSFRGSKRMPQYLKLQPHGEALTFEDGNVTLVESRKIIRHIADKYKNQGNPDLIGMGALERSSIEQWLQTEAQSFDVPSADVVYSLAYLPAATTQPNKGAAAADGGRCEEEKNDDGGRDRQYSSQRQGGAGAGGGRDGQMAAAHRQKVEEMKQLFEKSSKELSKVLDIYEQRLEEAEYLAGDKFTLADLSHLPNADRLAADPRTLRMLQSRRNVSRWWADVSGRESWKQVKSLNRPPSAEAPF*

>OsGSTF7

MSPVKVFGRAISTNVSRVLVCLEEVGADYELVTVDFLAGEQNSPEHVERNPFGKIPALQDGDLVLFESRAIAKYILRKYKSSKVDLLRESDIREAALVDVWTEVEAHQYYPALSPIVFECIIFPIMRGVPTNQQVVHESLEKLKKVLETYEARLSGSRYLAGDFLSFADLNHFPFTFYFMATPCASLFDAYPHVKAWWEGLMSRPSIKKISANMPTKF*

>OsGSTF8

MAPVKVFGPAMSTNVARVLVCLEEVGVEYELVNIDFKAMEHKSPEHLKRNPFGQMPAFQDGDLLLFESRAVGRYILRKYKTSEANLLREGNLTEAAMVDIGIEVEIHQYYPVISSIVYECLFNPAMYGVPTNQKVVDNSLEKLKKVLEVYEARLSQNTYLAGNFLSFVDLSHFPFTFYFMATPYASLLDKYPHVKAWWDGLAARPSIKKVTAAMVLPLKA*

>OsGSTF9

MAPVKVFGPAKSTAVARVLVCLEEVGAEYELVGIHIPAGEQKSPAHLARNPFGQVPAFQDGDLILFDLLKESNLSQSAIMVDVWLEVESQTFDTAMSAITFQCLTIPTFMGGIADDKIVEENLGKLKKALEVYEARSCRFRYLAGDFISLADLSHFPMTHYLLATPHASVLDAYPHVKSWINDLMKRPAVKRVRELMEA*

>OsGSTF10

MAPAKVYGPAMSTNVMRILVCLEEVGAEYEVVPVDMSTGEHKRPPHISRNPFGQVPAFEDGDLTLFESRAISKYILRKHGSDLLRESNLSESAMVDVWLEVESSHFDGAMSPIIFQCFIVPMFMGGATDIGVVNESLEKLKKALEVYEAQLSKSKYLAGDFISLADISHFPTVYYLLASAHASVLEAYPRVKAWIDDVMQRPSVKKVTEALKMPSA*

>OsGSTF11

MEKTCQAYLEQTLCWTSSLLIDFFQLLTPARREAGVEYEVVPLSLTNGDHRRPEHLARNPFGQIPVLEDGDLTLYQSHAIARYVLGKHKPELLGLGEGGSVEESAMVDMWLEVETHQYEAAVKPIVWHCLVHQHVGLVRDQGVVDESVEKLRAVLEVYEARLSSSSAGRYSYLAGGGSGDRVSLADLSHVPLMHYFTATEYGGVLGEYPRVKAWWEALLARPSVKKVIAGMPTDFGFGSGNLP*

>OsGSTF12

MAMKVYGLPMSTNVARVLVCLEEAGEQYEVVPIDFSIAEHKSPEHTSRNPFGQVPALQDGDLILFESRAISKYVLRKNNSELLKEHNLSDAAKVDVWLEAESHHFDEPMSVVIYQCLILPVYFGGQTDAKVVEENLEKLKKTFQVYEERLCKFRYLAGDFLSLADLSHFPTAYYLLATPHAAMLDEFPLVKAWIDGMLARPSVKKVIEMMKATA*

>OsGSTF13

MSPVKVFGSAPFTNVARVLLCLEEVGADYEIVDVDFGDREHKGPDHLARNPFGQVPAFQDGDLMLFESRAICRYILRKHRATDEANLLREGDPSESAVVDAWLDVEALRYEPSVHAVFVQRRVVPALGGEPDERVIAESVARLRETLAVYEARLEATRGYLAGGEVSLADLSHFPYTRYFMEMPYEVPVFGAYPRVTAWWERLLTRPSVRKVAAMMSGGEG*

>OsGSTF14

MAPASVKVFGSPTSAEVARVLMCLFEKDVEFQLVRVDAYRGTQRMPQYLKLQPLGEALTFEDDNLTLSESRGILRHIAHKYARQGNPDLIGTGALERASIEQWLQTEAQSFDVPSAEMVYSLAFLPPNMPKQNDNGNGNGNGYGNSNGREVQVANASSKRVVAGATDGKTAASGANGNKQQQKEEEMRKVFEKSKKDLEKLLDIYEQRLEEAAYLAGDKFTIADLSHLPNADRLASDPRSRRMFEARKNVSRWWNNISSRESWEYVKSLQRPPSAAHAGNAQQQQQQQSPSAGNNYQHQQGQGQGQQHYRNEQVENYNN*

>OsGSTF15

MAAGLQVFGQPASTDVARVLTCLFEKNLEFELIRIDTFKKEHKLPEFIKLRDPTGQVTFKHGDKTLVDSRAICRYLSTQFPDDGNRTIYGTGSLERASIEQWLQAEAQSFDAPSSELVFHLAFAPQLNIPADEARIAENERKLQQMLNVYDEILAKNKYLAGDEFTLADLSHLPNSHYIVNARSPRGKKLFTSKKHVARWYEEISNRASWKQVVKMQSEHPGAFE*

>OsGSTF16

MAAGLQVFGQPASTDVARVLTCLFEKDLEFELVCIDTFKREHKLPEFIKLRDPNGQVTFKHGDKTIVDSRAICRYVCTQFPEGNKTLYGTGSLERASIEQWLQAEAQNFSPPSSALVFHLAFAPHLNIPQDHAVIAENEKKLQQVLNVYDEILSKNEYLAGDEFTLADLSHLPNSHYIVSSERGRKLFTGRKNVARWYDQISKRETWKQVVKMQREHPGAFE*

>OsGSTF17

MVAKVYGVAASPYVATVLVCLEEAGASYELVAVDMAAGENRSRHHLARSPFGKIPAFEDGEVTLFESRAIQRYVLRNYNKPDLLREGNLEESAMVDMWMEVEAHHYDPAIFHIIRECVIKPMIGGGARDQAIVDENVEKLRKVLEVYERRLSESEYLAGDFVSVADLNHFPYTYYLMTTEYATLVESCTNVKAVEIMGI*

>OsGSTT1

MQPLLKVYADRRSQPSRAIIIFCRVNRIDFEEVTVDLFKREHLSPEFKKINPMGQVPAIVDGRFRLFESHAILRYLATVFPGVADHWYPADLFTRAKLEAILDWHHSNLRRGAATFILNTVLAPSLGLPSSPQAAKEAEKVLFRSLGLIESMWLKGNAKFLLGNPQLSIADLSLVCEIMQLEVLGDSERDRILGPHEKIRSWVQNVKKATSPHFDEVHELIFKMKERMAAKRQSEPSKDLKTASKL*

>OsGSTU1

MAEEKELVLLDFWVSPFGQRCRIAMAEKGLEFEYREEDLGNKSDLLLRSNPVHRKIPVLLHAGRPVSESLVILQYLDDAFPGTPHLLPPANSGDADAAYARATARFWADYVDRKLYDCGSRLWRLKGEPQAAAGREMAEILRTLEAELGDREFFGGGGGGRLGFVDVALVPFTAWFYSYERCGGFSVEEVAPRLAAWARRCGRIDSVVKHLPSPEKVYDFVGVLKKKYGVE*

>OsGSTU2

MAAEGELKLLGTWASPYVSRVKLALHLKGLSYEYVVEEDHFNNKSELLLSSNPVHKKVPVMIHNGKPICESLIIMEYLDEAFPDTAAPLLPADLHDRAVARFWAAYIDDKLVPSWKQAFSGKTGEEKAEGMRHMLAAVDALEAAMEWSYKGKPFFGGDAVGFLDVALGGLLSWLHGTEELCGAKILDAAKTPLLSAWARRFGEMDAAKVALPDVCKLVEFAKMKRVQLEAAMAATTVSRN*

>OsGSTU5

MADEVVLLDLWVSPFGQRCRIALAEKGVEYEYSEQSLADKSDLLLRSNPVHKKVPVLLHAGRPVCESLVILEYIDETWPPEPEKKKESPRLLPSDPYARARARFWADYVDKKLFDCQTRLWKLRAGDAAHEQAKRDMAEALGTLEAELGEGDYFGGEAFGYLDVVLVPFVAWFHAYERLAGFAVAEICPRLVAWGERCKGRDSVAKTLTDPEKVYEFALYLKAKFGAK*

>OsGSTU6

MAAAGDGAGGGGEQLTVLGAWGSPFLVRVRLALNLKGLSYEYVEVDLAGKSDLLLAANPVHAKVPVLLHAGRPVCESMLIVEYLDEAFPSSSSSAAAATTILPAADDPYARAVARFWAAFVDGELLSGWMGIYDGGKTGEERAAALARTRAALDALEGALRERAGGRWFGGERVGLVDVALGGFVPAMLASEPTTGVRIVDADRTPLLAAWVERFCALEEAKAAMPPLERLIAAGKKRYADLQAAAAASE*

>OsGSTU7

MMNSSEPVKLIGAFGSPFVHRVEVALRLKGVPYELILEDMGNKSELLLAHNPVHKMVPVLLHGDRSAICESLVIVEYVDEAFDGPPLLPADPLRRAMARFWVHFLDENCLKPLRPALFGEGEEQKKSMEETREGLTVAEAELRGKRFSGGSSIGLADIAGGGVLAHWVGVLQEVAGVSVLSDGDGEYPALRRWAKDYVANESVMECLPDRDRLLSYFTGIKQKCVSVAKSTLPKYSLLP*

>OsGSTU8

MAGHDELKLLGHWSSAYVTRVKLALHLKGVSYEYVEEDLRNKSDLLLASNPVHKTVPVLIHNGNPIRESQIIVQYIDEVFSGAGDSILPADPYERAVARFWAAYIDDKLLAPWKKVFRAKTEEERAAWMKQMFVAVDVLEGGLKECSKGKGCFFGGDSVGYVDVVLGGAVSFVHANDMITGGKLFDAARTPLLAAWLERFGELDAAKAVLQDVDRAVEHTKVRYARNAATAAND*

>OsGSTU9

MAGAGRDELKLLGMWASPYVSRAKLALQLKGVSYEYIEEDLGNKSDLFLRSNPVHKTVPVLIHNGNPICESSIIVQYIDESFPSSAASLLPADPYDRAVARFWAAYIDDKVRARVNCNLTLVYFHRCDAAHRRVDCAASGAVENGVQGEDGGGEGRAHEADARGGGRAGGRTEGVLQGEGMLLRRRQRRLRRRRAGWPRVVGARQRQALRRQALRRRQGAAAGGVAGALRRAGCRQGRPAGRRQGGRVRQEVPAKGFRHGCRSTSSKLGRHCDVQEKY*

>OsGSTU10

MAGDGELKLLGMWTSAFVLRVRFVLNLKSLPYEFVEENLGDKSDLLLASNPVNKTVPVLLHAGRPVNESQVILQYIDEAWPDRPPAVLPSDPYERAVARFWAAYVDDKVRLAWLGILFRSETEEERAAAVAQADAALETLEGALRECSGGKPFFGGDGVGLVDVVLGGYLGWFTAIKKLIGRRMIDPARTPALAAWEDLFRATDAARGVLPDDADKMLEFRQTALALGASKKITL*

>OsGSTU11

MAAGAGGDGGGELKLLGTWASPFVQRVRLALNLKGLAYEFIEEEIGGGKSELLLASNPVHKKVPVLLHRSNPICESQVIVQYLDDAFPGGAAGGDLLPSDPHARAVARFWAAYIDAEFFAPWNRSFYTASEEEKTAEMGRAAAALATIERAFAELSRGKGFFSGEDRPGFVDVVLGGFVGSMRAYGTAVGVEVLDAGRTPLLVAWAERVAALDAARGVIPDVERVVELSRYARKK*

>OsGSTU12

MAGGGDELKLLGMWASPYVLRVKLALSLKGLDYEYVEEDLKNKSELLLSSNPVNKKVPVLIHNGKPVCESQIILQYLDEAFPDAGATLLPADPHERAVARFWAAFCDDTIAKASQQASSGKTEEEKAEGEKKVVEALEKMEVGLSECSKGKPFFGGDTVGYVDIVLGSFLAWVRAGDAMKGVKRFDPATTPLLAAWAERFVELDVAKAAMPEVDKLIELAMARMAGAAAAATN*

>OsGSTU13

MAGKDDDVKVLGVVVSPFAIRVRIALNIKGVSYEYVEEDIFNKSELLLTSNPVHKKVPVLIHGGKPISESLVIVQYVDEVWAAAPSVLPADPYDRAVARFWAAYVDNNMFPGMAGVLFAATEEERAAKAEETLAALAQLEKAFAECAGGKAFFGGDSIGYVDLALGSNLHWFEALRRLFGVALLDAGKTPLLAAWAKRFVEAEAAKGVVPDAGVAVELGKKLQARAAAASTAA*

>OsGSTU14

MAGEGDDQLKLLGLWVSPYTHRVKLALSFKGLSYEYVEEDLSNKSELLLSTNPVHKKVPVLIHNGKPICESQVIVQYLDEEFPNSGVSLLPSDSYDRAIARFWAAYINDKLMPSWLQSSMGKTEEERAEALKQTLEAVANLETAFKECSKGKPFFGGDTVGYLDVSLGAMIGWMRAGEALHGRRTFDATRSPLLNAWMERFAALDAAKAAMPDNNKLVEFVRVRRAAAANN*

>OsGSTU15

MSSTNNSSGEPPPAVRVLGGWASPFTNRVVVALKLKGVEHEMLQETVGKKSELLLRSNPVHKKFPVLLHHSKPLPESLVIVEYIDEVWPASNGGAPAILPRDPHGRAVERFWARYVDDKILPGLRVLRGSVAGDKDQTAGEMSTTLQRLEEAFVKCSQGKEYFGGDSIGYLDIALGSFLGWIKAVEKIAGVELLNETKLPILAVWADRFCAHPAVVDVVPDADKLVEFTVQYGSVLNTVNVLPK*

>OsGSTU16

MEKQENGGEAPELKLFGSWASSYTHRVQLALRLKALEFVYAEEDLGNKSEALLRLNPVHKKVPVLVHRGRPLAESVIILQYLDDAWPESRPLLPSDPFDRALARFWCHFADDKLGPAVGAVFALTGREQEAAVQQVHDNLALLEAELREGAFKGRRFFGGDQVGLLDVVLGCGSYWLAVFEEVTGVRLVDADAFPLFHAWLRDFEAQEEVKETIPSVDRLLEYARGLRQMLLALAAGAGAGAAAAAGSSPVTTAVDAPAPAAPPAAPQAAAVDI*

>OsGSTU17

MAADKGVKVFGMWASPMAIRVEWALRLKGVDYEYVDEDLANKSEALLRHNPVTKKVPVLVHDGKPLAESTVIVEYIDEAWKHGYPIMPSDPFDRAQARFWARFAEEKCNAALYPIFMTTGEEQRKLVHEAQQCLKTLETALEGKKFFGGDAFGYLDIVTGWFAYWLPVIEEACGVEVVTDEALPLMKAWFDRVLAVDAVKAVLPPRDKLVALNKARREQILSA*

>OsGSTU18

MAAGGGGGDELKLLGLWASPYVLRAKFALSFKGLSYENVEEDLHNKSELLLSSNPVHKKVPVLIHNGKPICESQIIVEYVDEAFPDAGESLLPSDPYDRAVARFWAAYINDKFMPAWQKASLGLTEEEKAEAVKQMLAAIENLETAFKELSKGKPFFGGDTAGYLDVTLGAVVGWARAGEVLFGRKLFDATRSPLLAAWMERFVALDAVKAVLPDNAELIEYGKMRMAHYAKLAAALAAANKK*

>OsGSTU19

MAGGGEELKLLGMWASPFALRAKLALSFKGLSYDYVEEDFKNKSDVLLSSNPVHKKVPVLIHKGKPICESQVIVQYIDEVFPDAGVTLLPADPHDRAVARFWAAYIDEKLFSAWILVFRSKTEEEKAEAVKQTFAVVEKLEGALSECSKGKPFFGGDTVGYVDVVLGGFVAWVHAIEEVFGLNQFDAAKTPLLAAWLERFDELDAVKEVMPDIGRLVELAKMRQAQAAGAAAAAAGEAS*

>OsGSTU20

MAGGGDDLKMLGVYVSPFPLRVKLALSFKGLSFEYVEEDLHNKSDLLVSSNPVHKRTPVVIHNGKPISESMVIVQYLDEAFPGAGAALLPSDPLDRAVARFWASYVDDKLFSAWKMVFKGKTEEEKVEGRKQTFAVAETLEGALRECSKGKPFFGGDAVGYVDVALGGFVPWVHAMEELFGLKQFDAAKTPLLAAWLERVGELEAYKAVMPDAGMMIEFKKKQAQEAAAEA*

>OsGSTU21

MSSSSTSGSAEPAAAVRVLGSWTSPFVMRVVVALKLKGVEEYELLQETRGKKSELLLRSNPVHKKIPVLLHHGKPLAESLIIVEYIDEVWPASDGAPAILPRDPYCRAVERFWAQYIDDKFPRGTRVLRGTVAGEEPWLKCPPL

>OsGSTU22

MAGGGDELKLLGMWASPFALRAKLALSFKGLSYDYVEEDFKNKSELLLSSNPVHKKVPVLIHNGKPICESQVIVQYIDEVFPDAGVTLLPADPHDRAVARFWASYIDEKLFGAWIPVFRGKTEEEKAEGVKQTFAVAEKLEGALSECCKGKPFFGGDTVGYVDVVLGGFVAWVHAIEEVFGLNQFDAAKTPLLAAWLERFDELDAAKEAMPDIGRLVELEKMRQAQAQAAVY*

>OsGSTU23

MAGKDDDVKVLGLVMSPFAIRVCIALKLKGVSYEYIEEDLANKSELLLSSNPVHKKIPVLIHGGKPVSESLVIVQYVDEAWAPSPTSPSILPADPYDRAVARFWAAYVDDKMVPGMVGVLRAATEEERAAKADETLAAMAQLEKAFAEVAAKNGKPFFGGDTVGYVDLALGCNLHFLEAIRRLHGVALVDAGKTPLLAAWAERFVEVEAAKGVVPDADDAVEFARKVQARVAAAAASTAAK*

>OsGSTU24

MAGGGDELKLLGMWASPYVLRVKFALSLKGLSYEYVEEDLMNKSDLLLSSNPVNKKVPVLIHNGKPVCESQVILQYLDEAFPGAGATLLPADPHERAVARFWAAFNDDTLVEASQAASWGKTEEERAEGEKKVVEALEKMEVGLRECSKGKPFFGGDTVGYLDVVLGGFLAWVRATDVMRGVKRFDPATTPLLAAWAERFVELDAAKAVMPDMDKMIEFGKVLQARAAAATN*

>OsGSTU25

MAAEDELKLLGFWASPYVCRVKLALHLKGLIYDYVKEDVFTNKSELLLSCNPVHAKVPVLIHNGKPICESQVIVQYIDEVFPDAGVTLLPADPHDRAAARFWAAYIDDKLLPPWVHAYRGKTDEEKAERMKQTLAVVDALETAMEECSKGNAFFGGDTVGYVDVALGGLLSWLHGTEELCGAKILDAAKTPLLSAWARRFGELDAANAALPDVGRLVEFCKMRHVELEAAEAAAARN*

>OsGSTU26

MAGGGDELKLLGTWFSPFVSRVKFVFHLKGLSYENIEEDLKNKSELLLKSNPAIKKVPVLFHNGKPLCESMIIVEYIDETFAGVGPSVVPTDAYERAVARFWVSYIDNKLVAPWFQVFRSKSMEEKAEGLKQIFVAVMVLEEAFKECSKGRPFFGGDNAGIVDIALGSQLGWVRASQALSGIKLFDPAKTPLLAAWAERFLALDAAKASMPEFGRLIEYAKMRQAESDAANAAAN*

>OsGSTU27

MAGGGDELKLLATWFSPFASRVKFVFHLKGLSYENIEEDLKNKSELLLKSNPVIKKVPVLLHNGKPLCESMVIVEYLDETFAAVGPSVVPADPYERAVARFWVSYIDNKLVAPWFQVFRGKTKEEKAEGLKQMFEATAVMEVAFRECSKGRPFFGGDAVGIVDVALGSQLGWLRASETLSGIKLFDPAKTPLLLAWAERFLALDAAKASMPESGRLLAYAKMRQAETDAANASK*

>OsGSTU28

MAGRNNHELKLLGTWPSPFVVRVRLALGLKGLSYEYVEQDIRDKSELLVVSNPVHKKVPVLIHGGKPVCESQIIVQYIDEAFPGAGASLLPSDPHERAVARFWATYIDDEFATKFRAMGEAKEEEEKDEAAAQVFAALETLEEAMKGKVFFGGDSAGYVDVALGGFLGWIKAAEALAGVAFLDGARTPLLAAWAARFSALEAAKEAIPSVERLREFHGAMHAAAATVAGN*

>OsGSTU29

MAGAGDELKLLGMWTSPFALRVKLALSFKGLSYEYAEEDLSNKSELLLSSNPVHKKVPVLIHNGKPICESQVIVQYIDEAFPGAGVPLLPSDPYERAVARFWAAYIDDKLLKSWLQASMCKTEQEKAAAMKETFAAVANLEAAFKECSKGKPFFGGDAVGYVDVTLGAVIGFVRVGEAVHGMRLFDASRSPLLDAWLDRFAALDAAKAVLPDTGRLAEYAKMKQAEWAAAATN*

>OsGSTU30

MAGGGGAGELKLLGHWASAYVTRVKLALHLKGVSYEYVEEDLRNKSDLLLASNPVHKTVPVLIHNGNPIRESQIIVQYIDEAFSGAGDSLLPADPHERAVARFWTAYIEDKLVAPWEKVFRAKTEEERAAWMKQMFVAVEALEGGLKECSKGKGCFFGGDSVGYVDVVLGGGVSFVHANDVITGGKLFDAAKTPLLAEWLGRFGELDAAKAVLQDVDRAVEYTKVLYARNAATTAANN*

>OsGSTU31

MSSTNSSGDPAAVRVVGGWASPFMNRVVVALKLKGVEHEMLQETVGKKSELLLRSNPVHKKIPYIDEVWPASNGAPSILPRDPYGRAVERFWAKYIDDKIPPGIRVLRGSVEEDKDKAAGEMSTALQHLEEAFVKCSQGKQYFGGDNIGYLDIALGSFLGWIRAVEKIAGVELLNETKVPNLAAWADRFCAHPAVVDVVPDADRLVEFTVQHAALLRAVNVPK*

>OsGSTU32

MAHHHFFAPRKQRRSAAQERGKRELDGMDQEVWADMGTAVRVGAPPLLHLCTQLLPHPTAAGKLLLSSSNGMQPPHCTKKKQIAKMSSTNSSGDPAAVRVVGGWASPFMNRVVVALKLKGVEHEMLQETVGKKSELLLRSNPVHKKIPVLLHHGKPIAESLIIV*

>OsGSTU33

MAAAAAGGGGSPDHELKLLGSTNPSPFVTRVELALALRGLTYDLVAVDLDRKTDLLLAANPVHAKVPVLIHRGRPVCESRVILEYIDDAFPFPGGGGAPLLPPADDPLARAAARFWAAHVDDEFVASWRPAYLGSTEGERAEGMARMAAAVGALEGALAAAEGKPFFGGDAPGLVDVTLGSVIPRTRANEALTGTRVLDAARTPLLAAWAERFGELDAARKVLPAVGDVVEYLETRLRRSNVVIARKQ*

>OsGSTU34

MAAAAEEGEGVRLLGGRMSPFTMRARMALALRGVEYELVEEALHPRKSGRLLAANPAYGRIPVLLLPGGRAVCESAVIAQYVDDAWGGAGAGAAILPVDPYERAMHRFWTAYIDDKFWPALDAISLAPTPEARATATASTRAALKLLEEAFAARSNGGAFFSGGGAAASPGLLDVALGCFLPALWACENLNGLRLLDDDATPLLRAWSARLAATPAAMAVMPETEEVVAFTRFLQTKFGVAGSK*

>OsGSTU35

MGERVKLIGAFASAYGHRAEVALRLKGVRYELILEDLRNKSDLLLNHNPVHKLVPVLLHGDRSLSESLVILEYIDESFHGPPILPTDPYDRAVARFWAQFIDQKFGRFNFWIPFVQMEGNMQDCFVREAKENLALLEGQLKGRRFFGGDAIGFLDIAACLIAHWLGAFEEVCGVTLATDEEFPALCEWRRRYVNDEAVKPCLPNRDELVAYYRERKEMIKAAGRQHK*

>OsGSTU36

MADPVKLIGAFGSPFVHRAEVALRLKGVAYEFIHEDLDNKSDLLLAKNPIHKKVPVLLHGDRAICESLVIVEYADEVFDGRPILPTDPYDRAMARFWAHFIEHKCSRSSWLALWLDGEEQEGLLKETKENLALLEAQLHGKRFFAGDSVGYLDIVASGLAHWISVVEEVTGVSLMGGADEDDEYPALRRWAKEYTTDETVMQCLPSREHLAAFFAAKKDKLKMVAKAMLHQ*

>OsGSTU37

MADPVKLIGAFGSPFVHRVEAALQLKGVAYELIHEDLENKSNLLLASNPVHKKVPVLLDGGRAICESLVIVEYVDDAFDGPPILPADPYDRATARFWAQFIDHKCTLPLLLALWLDGEEQKGFLKETKENLSLLEAQLEGKRFFAGDAVGYLDVAAGGMAHWIGVLEEVTGVSVIGSEDDDEYPSLQRWIKEYANIDAVKLSLPDREELVAFYTRNKDKYKMMFRAMVHQ*

>OsGSTU38

MAMTTAAVAAAAQPKEVKLYGAWGSAHAAMARNALELKGVRYEYVEEDLERKSETLLLRLNPAHAGKVPVLVVVDDDGGGGGCPLAESLVILEYVDEVWPQAPRLLPPPSSPRARAAARFWARFFHGEVSPLSRAAAVLAPTPEERAEAVREMKARMAVMEAGFERDFPSSVVGGPFVHGATPGLLDVILGSCAAGTRAISAMAGEEVVEPDALPHVHASMAAFDERVAGFGTSVPHELLLARLLEREERRRAAASASA*

>OsGSTU39

MAGRGGGGELRLLGTWSSPWVIRVRVALGMKGLSYEYTEEDLSSKSDHLLRSNPVHEKVPVLIHGGRPVCESLVVLEYIDETWGATGTPQLLPADPYDRATARFWTNYVNDTFFPSWKVLFRSTAAEQRAEAFKNVVPRVEALERAFGECSKGKAFFGGDDAGLVDVALGSHLVWIKVVDEVAGANLLDEAKFPGLAAWAERFLAVDAVRQVMPDAGDVLKQYKGFLAKWTAGAGSS*

>OsGSTU40

MAGRGGGGGGGELQLLGTWYSPYAMRAKIALGLKGLSYEYIEQDLFGKSELLLKSNPVHKKVPVLIHAGRPVCESRVVLEYVDEAWPGAAPPLLPADPHDRATARFWATYFDSTFFPPWRALMRATTAEQRAEAFMNAVPQVEVLERAFVECSKGKAFFGGDAVGLVDVVVGGFVVWFKVVDEVAGSSLLDEAKFPGLAAWAERFLAVDAVREAMPDAGKLLEHYKGFLAKLASPAGST*

>OsGSTU41

MVKLISAFGSPFGHRAEAALRLKGVQYELLLEDLRSKSDLLLAHNPVHKLVPVLLHSDGRSVAESLVVVQYVDDAFHGPPLLPADPYARAQARFWAQFIDDKFSRPFWLSFWMEDGEKKEAFVREAKENLRPLEAQLDGGNKRFFGGDAIGLVDIAASGLAHWVGVFEEVTGVSLVSEREFPALCRWSQRYVNDGAVRQCLPSRDELVALFTANKEAYTLLAKAKLQK*

>OsGSTU42

MASPAPAPVKVIGTFDSPYSQRAEAALRLKGVPYELILEDLRNKSDLLLTHNPIHKKVPVLLHGDDDHRAAVCESLVIVEYVDEAFPAPLLLLPADPGLRAAARFWARFIDDKCTKPFWLALWSTDDGEVREGFAAEIKENLKLLEAQLKGRRFFGGDAIGYLDLAASGYAYWLEVLEEVAGVSLVTGDEFPDLCRWAKEYAADDRIKACLPDRAKLLEHFTAMKEMFMATARSMAAK*

>OsGSTU43

MANLVKLIGAFGSPFVHRAEVALRLKGVAYEFIHEDLNNKSDLLLAKNPIHKKVPVLLHGDRAVCESLVIVEYIDEAFNGPPLLPADPYHRAMARFWAHFIDHKSTRPSWLALWLEGEEQKGFLKETKENLALLEAQLGGKRFFAGDSIGYLDIAAGGLAHWVGVLEEVTGVSLVAGDDGDDEYPALRRWTNEYTANDAVKLCLPNRERIAAFFTPKDKYKIMARAMLRQQ*

>OsGSTU44

MEGEKKSVVLINCAVSMYGNRVRIALARKGVAYEEKPENLAAKSALLLSSNPVHGQVPVLLVGGKPVCESLVILEFIDEEFAGVGEPLLPAGPYERAQARFWASYIDAKLAPCAGRVWRSPAGAAGAAAVEAARGELVAAMRTLEAELGGRRYFGGGGEALGYVDVALAPFTAWFATYERFGGFSVAAECPELAAWAARCVRENACVAASLPDPEFVYQFACGMRKHFGLDG*

>OsGSTU45

MRARIALHVLQVGFGFVEEDLRIRERSDLVLRMNPVHRSVPILIHRGRPICGSINILQYIDEVWAKRVGTRLLPPDPLKRASARFWADFVDHEVFSTQTRFLKSKGEEKEMAKAELLDQLRRLEGVLGDRSFFSGDEFGFLDIVLIPFSSMFHGYKQHMWVKRCKERESVRQVLPDEGEMYELHKKWYGIE*

>OsGSTU46

MAGGELVLLDFWASPFGQRCRIALAEKKLPYDYSEQELLGAKSDLLLRSNPIHAKVPVLLHGDGDGRAVCESLAILEYLDDAFPDATPRLLPSAADDPYARARARFWADYVDKKVYPVGTRLWKVKGEEGVRAAAGARGELVEALRTLDGELGEKEFFGGEFGFVDVALVPMMPWVYSFARYGGFSVEEECPRVAAWARRCMERDSVAGSLRSPEEIYDFIGLLRKHYGIDD*

>OsGSTU47

MRVMVALRLKGVEYELLQETMGKKSELLLASNPVHKKIPVLLHRGKPISESLVIVQYVDEVWPPPASILPRDDPYAAAIHRFWGQYIDDMFPPRIRILRGTVPGDKDEASDEMTTALLYLEEAFVECSKGKQYFGDDSIGYLDIALGSHLGWIRAVERIAGVELLGGAKVPNLAAWADRFCGHPAVVDVMPDVDILVEFTAKLI*

>OsGSTU48

MAEELKLLGSLSGVSPYVIRAQMALAVKGLAHDYLPEDLTRKSKLLLDSNPVHKSVPVLIHNGKPVCDSLVIVEYVDEAFPGGAAALLPADPYHRAVARFWAAFIDSKVFPPCLAILKTAAAEAEEEKAAKVKETVEALQLVEGAFGECSKRKPFFGGDAVGYLDVVLGCYLCWFEGVSEIAGGVSPPLLDASRTPQLAAWAARFRSAADAVGCSVPRVDKVEAYLNNVLKPKWSAAAAASSH*

>OsGSTU49

MVVGAGGGDELKLLGVWDSPYVNRVQIVLNLKGLSYEYVEEDLMNKSDLLLGSNPVHKKVPVLIHNGKPIAESRVIVEYLDEAFAAGAGGSTGASVLPSDPYERAVARFWAAYVDDKVGSPWYTILFARERGEKVEAAARAISALETVEAGAFRDRSSEGKTTNAAAAPFFGGDSIGFVDVVLGSYLGWFRVIEKMIGVRIMDAARTPRLAAWAERFEAADAVRGVLPDDVDKVIDFLQAFLH*

>OsGSTU50

MAGSGELKLLGVWSSPYAIRVRVVLNLKSLPYEYVEENLGDKSDLLLASNPVHKSVPVLLHAGRPVNESQVIVQYIDEVWPGGAGGRPSVMPSDPYERAVARFWAAYVDDKVRPAWLAILFGSKTEEERAAAVAQAVAALETLEGAFGECSKGKPFFGGDGVGFVDVVLGGYLGWFTAIDKLIGRRLIDPARTPALAAWEERFRATDAAKGVVPDDADKLLEFRQTLLRWSASKAK*

>OsGSTU51

MDHQELEEGAEKMKLLGIWSSPYVVKVIWALRIKHVEYDIEEDLRNKGNLLLECNPVHQKVPVLIYQGKPSDVIIEFIDDVWKDSGQGRIYSTQLSPPIWKWFTTQGKEQEDA*

>OsGSTU52

MWALRIKGVEYDYIEEDLRNKSNLLLECNPVHKKVPVLIYQGKPIAESDVILEFIDDVWKDLRYRILPEDPYECAMARFWSKFGLDKLSPPIWKWFTTQGKEQEDAYEAAMEQLLVLEKVLDEKKFFGGERIGFVDLSLGSLSYVIPIYEDITGVRLITSDKFPWLSAWMEGFLGLPLVKEHLLPLDKLRPRYQAIREAFLSK*

>OsGSTZ1

MAAAEKTKPVLYSEWMSSCSYRVRIALNLKGIDYEYRAVTRGDPDYGKINPIKYVPALVDGDFTISDSLAIILYLEDKYPQHPLLPQDLKKKALNMQIANIVCSSIQPLQCYAVIGLADGKMSANESLQIVQHYTDKGFRAIEKLLEGCRSKYATGDEVQLADVFLAPQIHAGITRFQIDMSKYPILARFYKAYMELPAFQAAVPENQPDAPSS*

>OsGSTZ2

MASSKPILYGAWISSCSHRIRIVLNLKGVDYEYKSVNPRTDPDYEKINPIKYIPALVDGDLVVSDSLAIALYLEDKYPQHALLPKDLKKKALNLQIANIVCSSIQPLQGYAVIGLHEGKLSPDESLQIVQHYIDKGFKAIEKLLEGSNFKYATGDEVQLGDVFLAPQIHAGINRFQIDITKYPNLARLHDTYMEIPAFQAALPKNQPDAPSC*

>OsGSTZ3

MAEAAGAAVAPAKLGLYSYWRSSCSHRVRIALNLKGLEYEYKAVNLLKGEHSDPEFMKVNPMKFVPALVDGDAVIGDSYAIALYLEDKYPEHPLLPQDLKMKALNLQIASIVCSGIQPLHNLTVLVRTDLHSISYCHRFIEKKVGTGESIPWTQQQIDRGFAAAENLVKGCAGKYATGDEVRLADVFLAPQIYAAVTRFQINMLNYPTLARLHEEYMKHPAFQAALPDRQPDAPSST*

>OsGSTZ4

MASSGSPEARQTHGEIAGAAAPERRLKLYSFWRSSCSYRVRIALSLKGLDYEYKPINLLANEQSHPEFEKLNPMKYVPALVDGDDTVVVDSFAILLYLEDTYPQHPLLPQDPKMKALNIQIASIVGSSIQPLQNNSVLDFIEEKLDSQEKVNWIQYHLNRGFTALEKMLKGCTTTYATGDEIQLGDLFLEPQIYGGIKRFGIDMTNYPTLARLHEAYMEHPAFQAALPERQPDAPSSPEI*

>OsDHAR1

MGRHVRMTITLPSPSRIPKPPEHGGTASWAPHVILTPSVSGELPLPINPASPISPVISPPIARRRRNPKSSLPVREKRQVVVAAMGVEVCVKAAVGHPDTLGDCPFSQRVLLTLEEKKVPYEMKLIDVQNKPDWFLKISPEGKVPVFNGGDGKWIPDSDVITQVIEEKYPTPSLVTPPEYASVGSKIFSCFTTFLKSKDPNDGSEKALLTELQALEEHLKAHGPFINGQNISAADLSLAPKLYHLQVALEHFKGWKIPEDLTNVHAYTEALFSRESFIKTKAAKEHLIAGWAPKVNA*

>OsDHAR2

MAVLLRTTTSATTATSGGSSSATALLATTFRRGGRRLLLLPATRGSAPRRAALLTARASAEPLEVCAKASLTVPDRLGDCPFTQRVLLTIEEKHLPYDIKLVDLANKPDWFLKISPEGKVPIVKLEEQWVADSDVITQAIEEKYPEPSLATPPEKASVGSKIFSTFIGFLKSKDPNDGTEQALLSELTSFDSYLKDNGPFINGETISAADLSLAPKLYHMEIALGHYKNWSVPDSLSHVKKYMKTIFSMDSFVKTIALQEDVIAGWRPKVMG*

>OsTCHQD1

MQLYHHPYSLDSQKVRMALEEKGIDYTSYHVNPLTGKNMNVAFFRMNPSAKLPVFQNGAHVIYRAFDIIQYLDRLSVHLSGEIVPVNTEVYQWMQKVDSWNPKMFTLTHTPIKYRTFVSKFIRRVLIARMAEAPDLASMYHAKLREAYETEDKLKDPDIMKQSEEELSKLLDDVEAQLNNGKYLAGDEFSPADSVFIPILARITLLDLDEEYINCRPRLLEYYTLVKQRPSYKVAIGKFFGGWKKYRTLFKTSFFLCVRTLFRKY*

>OsEF1γ1

MALVLHTFDGNKNAFKALIAAEYSGVKVELAKNFQMGVSNKTPEYLKMNPIGKVPILETPDGPVFESNAIARYVTRSKSDNPLYGSSLIEYAHIEQWIDFSATEVDANTGKWLFPRLGFAPYVAVSEEAAIAALKRSLGALNTHLASNTYLVGHSVTLADIVMTCNLYMGFARIMTKNFTSEFPHVERYFWTMVNQPNFKKVMGDVKQADSVPQVQKKAAAPKEQKPKEAKKEAPKEAPKPKAAEKPEEEEEAPKPKPKNPLDLLPPSKMILDEWKRLYSNTKTNFREVAIKGFWDMYDPEGYSLWFCDYKYNDENTVSFVTMNKVGGFLQRMDLCRKYAFGKMLVIGSEPPFKVKGLWLFRGPEIPKFVMDEVYDMELYEWTKVDISDEAQKERVSAMIEDLEPFEGEALLDAKCFK*

>OsEF1γ2

MALVLHCGSGNKNAFKALIAAEYTGVKVELTKNFEMGVSNKTPEFLKMNPLGKIPVLETPEGAVFESNAIARYVARLKDNSSLCGSSLIDYSHIEQWMDFSATEVDANIGRWLYPRLGFGPYVPVLEEFAITSLKRSLGALNTHLASNTYLVGHSVTLADIVMTCNLYYGFVRILIKSFTSEFPHVERYFWTMVNQPNFKKVIGDFKQAESVPPVQKKAAPPKESKAKEAKKEAPKEAPKPKVEASEEEEAPKPKPKNPLDLLPPSKMILDEWKRLYSNTKTNFREIAIKGFWDMYDPEGYSLWFCDYKYNDENTVSFVTMNKVGGFLQRMDLCRKYAFGKMLVIGSTPPFKVKGLWLFRGQDIPKFVMDEVYDMELYEWTKVDLSDEAQKERVNAMIEDQEPFEGEDLLDAKCFK*

>OsGSTL1

MAAAAAPRSSGKEALPAALGSASEPPRLFDGTTRLYICYFCPFAQRAWIIRNFKGLQDKIELVGIDLQDKPAWYKEKVYEQGTVPSLEHNGKIMGESLDLIKYIDSHFEGPALLPEDPEKRQFADELIAYANAFTKALYSPLISKADLSAETVAALDKIEAALSKFGDGPFFLGQFSLVDIAYVTIIERIQIYYSHIRKYEITNGRPNLEKFIEEINRIEAYTQTKNDPLYLLDLAKTHLKVA

>OSGSTL2

MAAAAAAPASSEKEVLPPSLTSSSEPPPLFDGTTRLYVAYHCPYAQRAWIARNYKGLQDKIKIVAIDLADRPAWYKEKVYPENKVPSLEHNNQVKGESLDLVKYIDTNFEGPALLPDDSEKQQFAEELLAYTDAFNKASYSSIVAKGDVCDEAVAALDKIEAALSKFNDGPFFLGQFSLVDIAYVPFIERFQIFFSGIKNYDITKGRPNLQKFIEEVNKIHAYTETKQDPQFLLEHTKKRLGIA

>OsGSTL3

MLSVTSTITLICICQIISTLLMYKKLQWQIVHVNICPYAQRAWIARNYKGLQEKIKLVPMDTNDRPAWYKEVYPKNTLPSLEHNNKIIGESLDLIKYIDINFAGPRLTPDDSEKQRLAEELLAYSDIFNQAVRSALISKDAMTAEAAAALDNIEFSLSKFDDGPFFLGQFSLVDIAYAPFIDGFQTLFAGIKNYDITEGRANIQIFIKELNKIDAYMHTKQDPSEVIALTKKKLGGRIHRWGLSLSSISAEPPALHAEQQPHGFGREAAYCISADQPGRKEGANEDNISRSMGD

>OsGHR1

MLTRLPHHSSPLVFPCRLSAAAAARTLSTATGSNSTTVKMARSALDEVTDAGAFDRSPSTFRSSISRDSSARFPAVPGRYHLYVSYACPWASRCLAYLKLKGLDHAIGFTSVKPIFERTRETDDHLGWVFPATGDEEPGADPDPFNGAKTIRELYEIASPNYIGKPTVPVLWDKQLKTVVNNESSEIIRMLNTEFNEIAKNPDLDLYPAHLQTSVNEINELVYDAINNGVYKCGFAKKQGPYDEAVTRLYEALDKCEEILSRQRYICGNQLTEADVRLFVTLIRFDEVYAVHFKCNKRLLREYPNLFNYTKDIYQIPGISSTVNMEHIRKHYYGSHPSINPYGIIPAGPNIDYNAPHDRERFSA*

>OsGHR2

MPMWSQPPPPPSSLQLRRPPPPLPHRPRRLRSRLSPIAASQDPLTALSRLLWGRALPPSQLVLAVRHGWTAAWGLLMRQLAPSDPATGAFTRTPSRFPAVVGTPSARLHLYVGLPCPWAHRALLVRALLGLERRLPLSVAVPGDDGAWSFTPDSPDALYGKRKLREVYASARRGGFEGRASVPLLWDAERREVVCNESIEITKFLCDLAAADGSAGGLDLWPPELRQDIDRWYSFIYPSVNNGVYRCGFAQSQEAYDAAAGELFAALDRLEDHLSGSRYLCGDTLTLADVCLFTTLVRFDLVYHSLFRCTRRKLVEYASLHAYTRDIYQMPGVAGTCDMAAIADGYFGALFPLNPGGILPLVPASCSPEALLEPHGREALSSSAAADAGGGGNGRQLEATSASN*
