# Supplementary figures and images for: Genome-wide identification and expression analysis of glutathione S-transferase gene family in tomato: Gaining an insight to their physiological and stress-specific roles
Source: PLoS One. 2017 Nov 2;12(11):e0187504. doi: 10.1371/journal.pone.0187504 (PMC5667761; doi:10.1371/journal.pone.0187504)

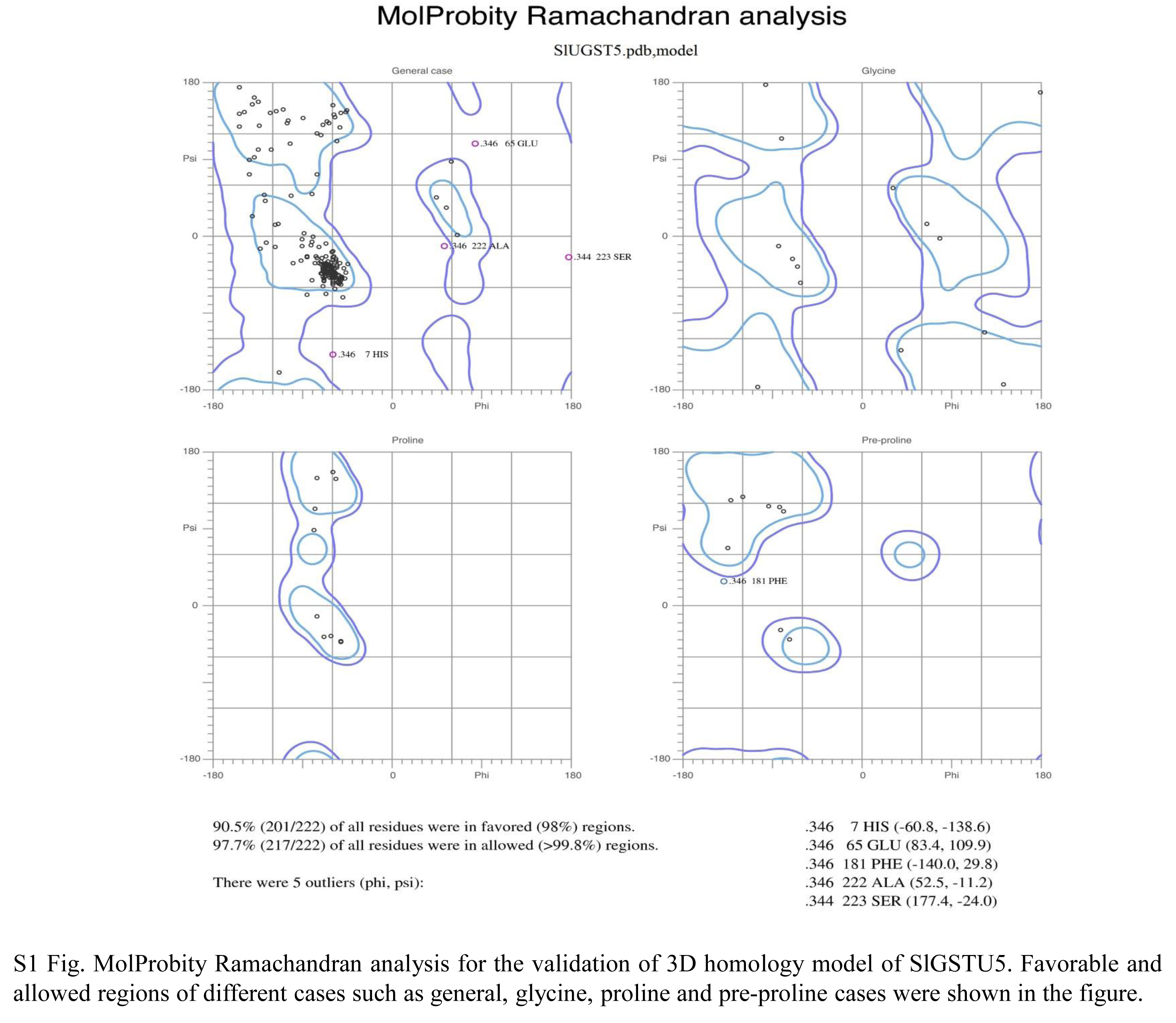

Supplement: S1 Fig — (TIF) [file pone.0187504.s005.tif]

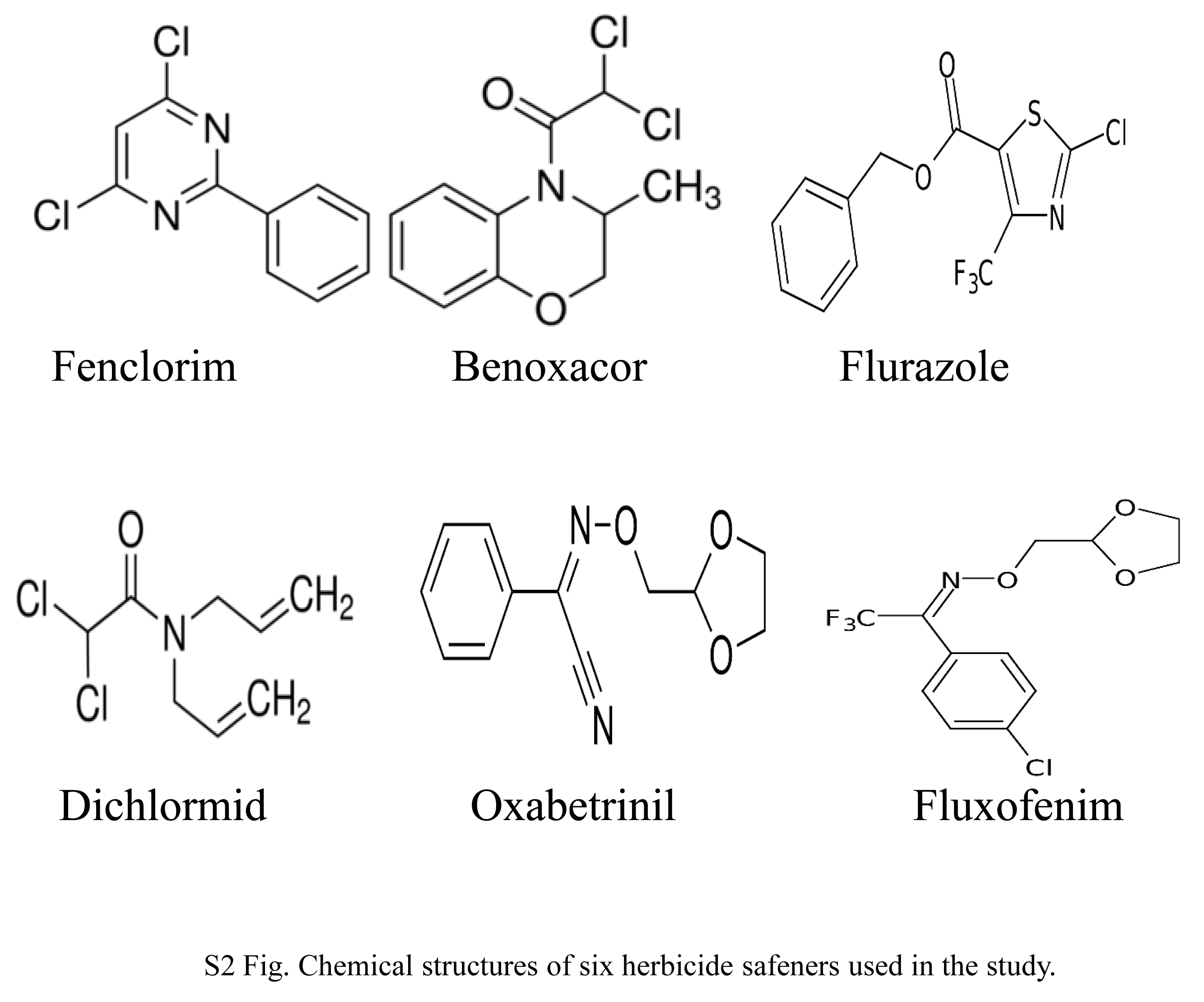

Supplement: S2 Fig — (TIF) [file pone.0187504.s006.tif]

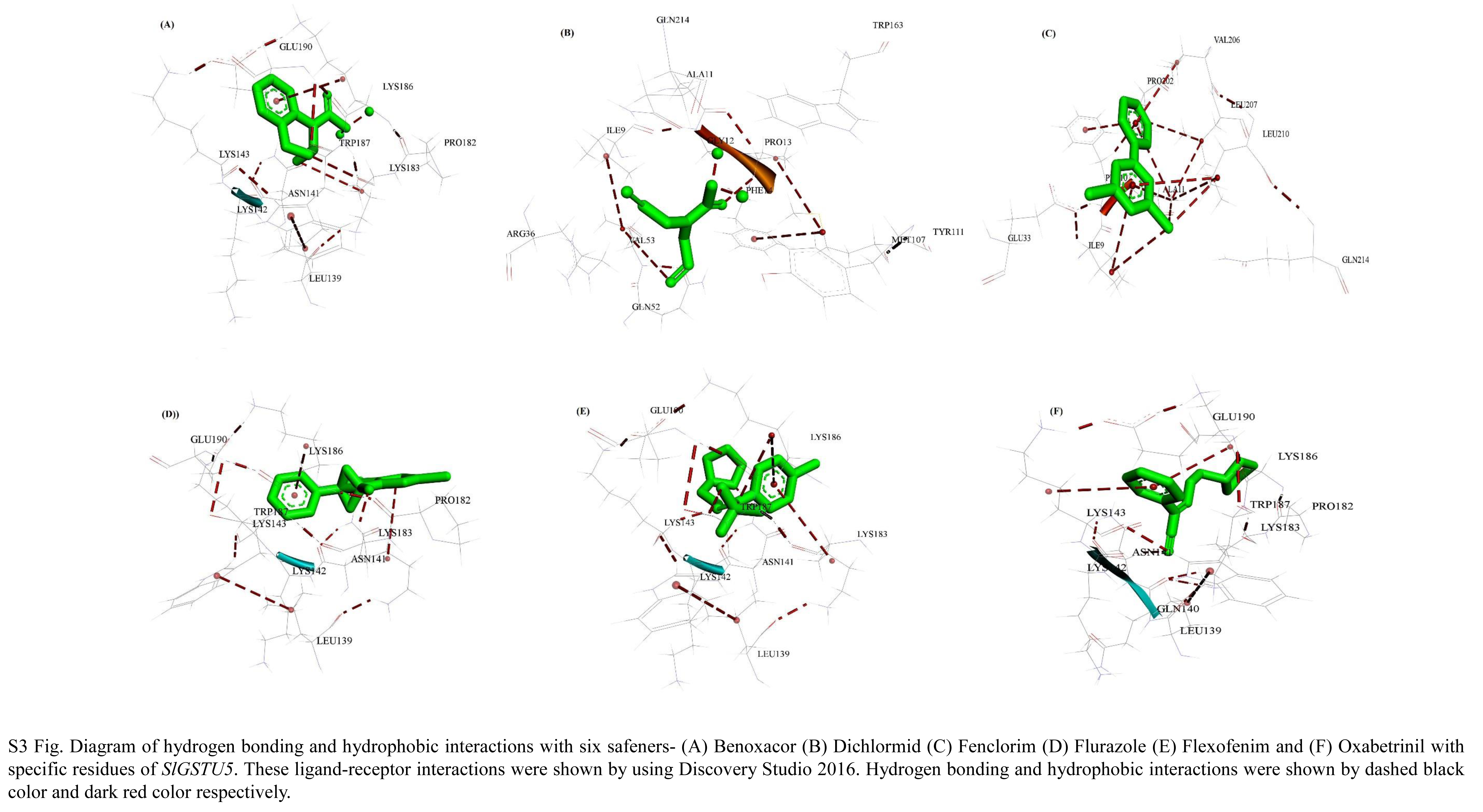

Supplement: S3 Fig — (TIF) [file pone.0187504.s007.tif]
